# Design, synthesis and biological evaluation of novel 1*H*-1,2,4-triazole, benzothiazole and indazole-based derivatives as potent FGFR1 inhibitors *via*fragment-based virtual screening

**DOI:** 10.1080/14756366.2019.1673745

**Published:** 2019-11-04

**Authors:** Jian Liu, Yu Wen, Lina Gao, Liang Gao, Fengjun He, Jingxian Zhou, Junwei Wang, Rupeng Dai, Xiaojing Chen, Di Kang, Lihong Hu

**Affiliations:** Jiangsu Key Laboratory for Functional Substance of Chinese Medicine, Jiangsu Collaborative Innovation Center of Chinese Medicinal Resources Industrialization, Stake Key Laboratory Cultivation Base for TCM Quality and Efficacy, School of Pharmacy, Nanjing University of Chinese Medicine, Nanjing, PR China

**Keywords:** Anticancer, FGFR1, FGFR1 inhibitor, Fragment-based virtual screening

## Abstract

Fibroblast growth-factor receptor (FGFR) is a potential target for cancer therapy. We designed three novel series of FGFR1 inhibitors bearing indazole, benzothiazole, and 1*H*-1,2,4-triazole scaffold *via* fragment-based virtual screening. All the newly synthesised compounds were evaluated *in vitro* for their inhibitory activities against FGFR1. Compound **9d** bearing an indazole scaffold was first identified as a hit compound, with excellent kinase inhibitory activity (IC_50_ = 15.0 nM) and modest anti-proliferative activity (IC_50_ = 785.8 nM). Through two rounds of optimisation, the indazole derivative **9 u** stood out as the most potent FGFR1 inhibitors with the best enzyme inhibitory activity (IC_50_ = 3.3 nM) and cellular activity (IC_50_ = 468.2 nM). Moreover, **9 u** also exhibited good kinase selectivity. In addition, molecular docking study was performed to investigate the binding mode between target compounds and FGFR1.

## Graphical Abstract

**Figure:**
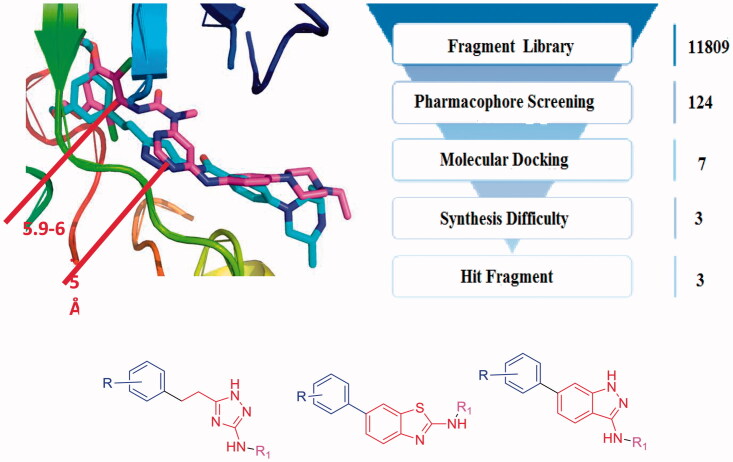

## Introduction

Protein kinases constitute one of the largest protein families in humans^1–5^. The kinase enzymes in this family catalyse phosphorylation of serine, threonine, or tyrosine residues, which regulate the majority of signal transduction pathways in cell, and thus play an important role in cell growth, metabolism, differentiation and apoptosis consequently. Deregulation of protein kinases is implicated in a number of diseases including cancer, diabetes and inflammation. Targeted inhibition of protein kinases has thereby become an attractive therapeutic strategy in treatment of relevant diseases^6^^,^^7^.

Fibroblast growth-factor receptors (FGFRs) form a sub-family of the receptor tyrosine kinase (RTK) superfamily, which consists of four highly conserved functional members (FGFR 1–4)^8^^,^^9^. FGFR signalling is initiated by binding of extracellular FGF ligand which leads to receptor dimerisation and cross-phosphorylation of the kinase domains proceed to phosphorylate intracellular substrates such as FRS2, Gab1, PLCγ, and STAT1. Subsequent downstream signalling is complex and includes activation of the PI3K-Akt and the Ras/Raf/Mek/Erk pathways. In normal cells, FGFR plays fundamental roles in many physiologic processes, including embryogenesis, tissue homeostasis, tissue repairing, wound healing, and inflammation^10–12^. Therefore, the inhibition of FGFR signalling pathway presents a promising option for cancer therapeutics.

**Figure 1.**
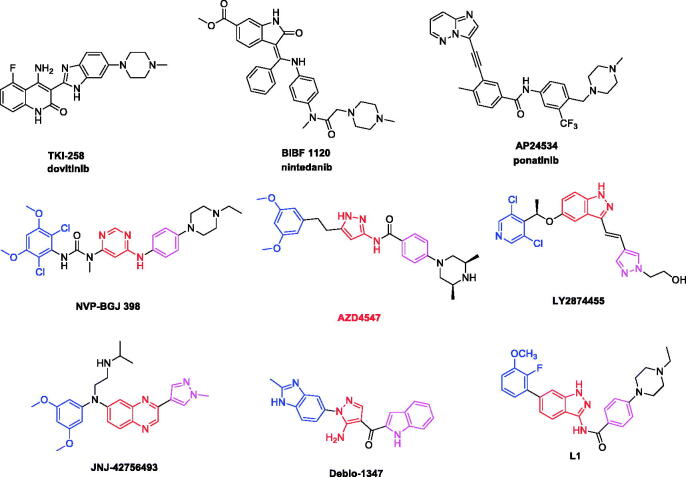
Structure of representative FGFR inhibitors.

In the past few years, different strategies have been pursued to inhibit abnormal FGFR signalling pathways, among them several small-molecule FGFR inhibitors have advanced into clinical trial (Figure 1)^13^. While the pan-kinase inhibitors (e.g. **dovitinib** (**TKI-258**)^14^, **nintedanib** (**BIBF-1120**)^15^, and **ponatinib** (**AP-24534**)^16^) which were originally adopted for the treatment of cancers that harbour aberrant FGFR, severe side effects were observed due to inhibition of several off-target kinases such as EGFR, VEGFR, PDGFR, etc. More recently, there has been an increasing interest in developing selective FGFR inhibitors. Several selective FGFR inhibitors bear various scaffolds have been developed into clinical investigation (e.g. **NVP-BGJ398**^17^, **LY-2874455**^18^, **AZD4547**^19^, **JNJ-42756493**^20^, and **Debio-1347**^21^).

In our previous studies, a series of indazole derivatives were discovered as potent FGFR1 inhibitors^22^. The most promising compound **L1** exhibited good enzymatic inhibition against FGFR1 and modest cellular inhibition (Figure 1). In this study, in order to improve the cellular inhibition, discover novel scaffold and enrich the structure-activity relationship, further work has been putting into modification of indazole scaffold and the hydrophobic substituents. The goal of this compound modification was to increase the ligand-receptor interaction, and improve the physico-chemical property. As a result, we designed the novel FGFR1 inhibitors by the following two strategies: (a) according to the fragment-based drug design strategy, we introduced novel flat hetero aromatic ring which formed key H-bond interaction with hinge region; (b) further optimisation of the original indazole inhibitors *via* introduced halogen atoms, that were often used to improve permeability through the modulation of modulation of compound’s lipophilicity and halogen-protein interactions.

**Figure 2.**
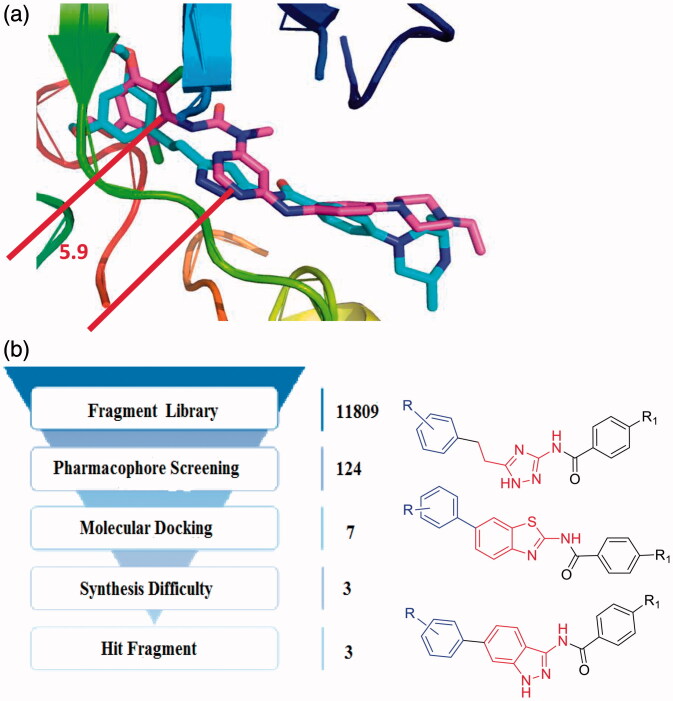
(a) Binding model of AZD4547 and NVP-BGJ398 to the FGFR1 kinase domain (PDB ID: 4V05, 3TTO). (b) The fragment-based virtual screening protocol.

Herein, with the fragment-based virtual screening strategy, we designed three novel series of FGFR1 inhibitors bearing 1*H*-1,2,4-triazole, benzothiazole and indazole scaffold (Figure 2(b)). The indazole derivative **9 u** stood out as the most potent FGFR1 inhibitor, and it also exhibited good kinase selectivity. Additionally, the docking studies were carried out to investigate the receptor-ligand interaction.

## Materials and methods

### Fragment-based virtual screening

#### Pharmacophore screening

The fragment library was derived from kinase hinge region directed library provided by enamine (https://enamine.net/index.php), which contained 11809 fragments. On the basis of the chemical features of FGFR1 inhibitors, these pharmacophore features, hydrogen bond acceptor (HBA), hydrogen bond donor (HBD), and hydrophobic (HY) were defined as a query for pharmacophore screening. The database screening was performed using the Ligand Pharmacophore Mapping protocol in DS 4.0. The fit values were calculated based on the chemical substructures map the location constraints of the pharmacophoric features and their distance deviation from the feature centres. Finally, only the compounds showed good fit values in pharmacophore model could enter the further molecular docking study.

#### Molecular docking study

Molecular docking of compounds into the three dimensional X-ray structure of FGFR1 (PDB ID: 4ZSA) was carried out using the surflex-dock module of the Sybyl-x 2.0 software package.The three-dimensional structure of compound was constructed using ChemBio 3 D Ultra software [Chemical Structure Drawing Standard; Cambridge corporation, USA 2010], then it was energetically minimised by using MMFF94 with 5000 iterations and minimum RMS gradient 0.05. The protein was prepared by the Protein preparation wizard of Sybyl-x 2.0. The waters were eliminated from the protein and the polar hydrogen was added. Receptor grids were generated using Receptor Grid Generation. The generated binding site was just the ATP-binding pocket of FGFR1, including several key amino acid residues: Glu562, Ala564.

Compound **9d** was placed during the molecular docking procedure. Types of interactions of the docked protein with ligand were analysed after the end of molecular docking. The detailed structures and calculation results were shown in Supplementary Table S1.

### Chemistry

Solvents were distilled under the positive pressure of dry argon before use and dried using standard methods. Chemicals were obtained from local suppliers and were used without further purification. All reactions were monitored by thin-layer chromatography (silica gel 60 F254 glass plates). NMR spectra were recorded on Bruker 400 MHz instruments, and the chemical shifts were presented in terms of parts per million with TMS as the internal reference. Electron-spray ionisation mass spectra in positive mode (ESI-MS) data were obtained with a Bruker Esquire 3000+ spectrometer. Flash column chromatography was performed on silica gel (200–300 mesh, Adamas, China).

### *General method for preparation of compounds 9a-9z* (exemplified by 9d)

The detail experimental procedures of intermediate **2**, **3**, **4**, **6**, **7** and **8** are described as previous reference 22 (see in Supplementary Material).

**4–(4-ethylpiperazin-1-yl)-*N*-(6–(3-methoxyphenyl)-1H-indazol-3-yl)benzamide (9d)** The building block **8** (100.0 mg, 0.2 mmol) was dissolved in dioxane (2 ml), then followed by the addition of (3-methoxyphenyl)boronic acid (85.2 mg, 0.5 mmol), Pd(dppf)Cl_2_ (20 mg), 1 *M* Cs_2_CO_3_ (500 µL). The reaction mixture was heated at 120 °C. for 1 h, then it cooled to rt. After concentrated, the residue was dissolved in EtOAc (50 ml) and washed with H_2_O (10 ml ×2), and brine (10 ml ×2), dried over Na_2_SO_4_, concentrated in *vacuo.* The residue was purified by chromatography on silica gel DCM-MeOH (10:1) to give the **9d** (45.6 mg, 44%). Mp 226.5–230.2 °C. ^1^H NMR (400 MHz, DMSO-d_6_) δ: 12.80 (brs, 1H), 10.53 (brs, 1H), 7.99 (d, *J* = 8.9 Hz, 2H), 7.78 (d, *J* = 8.6 Hz, 1H), 7.68 (s, 1H), 7.35–7.45 (m, 2H), 7.30 (d, *J* = 7.9 Hz, 1H), 7.24–7.26 (d, *J* = 2.0 Hz, 1H), 7.03 (d, *J* = 9.0 Hz, 2H), 6.97 (dd, *J* = 8.2, 2.0 Hz, 1H), 3.85 (s, 3H), 2.48–2.55 (m, 4H), 2.39 (q, *J* = 7.1 Hz, 2H), 1.05 (t, *J* = 7.2 Hz, 3H). ^13 ^C NMR (100 MHz, DMSO-d_6_) δ: 165.5, 160.2, 153.6, 142.6, 142.2, 141.1, 138.9, 130.5, 129.8, 123.1, 123.0, 120.0, 119.8, 117.0, 113.9, 113.6, 113.1, 108.3, 55.6, 52.6, 52.1, 47.4, 12.4. ESI-MS (*m/z*): [M + H]^+^ = 456.7 (Calcd: 456.55). HRMS: calcd for C_27_H_31_N_5_O_2_ (M + H)^+^ 456.2400, found 456.2391. HPLC analysis: MeOH-H_2_O (80: 20), 6.85 min, 96.8% purity.

***N*-(6–(3,5-dimethoxyphenyl)-1*H*-indazol-3-yl)-4-((*3R,5S*)-3,5-dimethylpiperazin-1-yl)benzamide (9a)** White solid: 29.2 mg, yield 38%; Mp 228.1–232.2 °C. ^1^H NMR (400 MHz, DMSO-d_6_) δ: 12.81 (brs, 1H), 10.53 (brs, 1H), 7.99 (d, *J* = 8.3 Hz, 2H), 7.77 (d, *J* = 8.0 Hz, 1H), 7.68 (s, 1H), 7.37 (dd, *J* = 8.6, 1.3 Hz, 1H), 7.04 (d, *J* = 9.0 Hz, 2H), 6.85 (d, *J* = 2.2 Hz, 2H), 6.54 (t, *J* = 2.1 Hz, 1H), 3.85 (s, 2H), 3.83 (s, 6H), 2.90–3.01 (m, 2H), 2.35–2.41 (m, 2H), 1.12 (s, 3H), 1.10 (s, 3H). ^13 ^C NMR (100 MHz, DMSO-*d6*) δ: 165.7, 161.3, 153.2, 143.3, 142.1, 141.0, 139.0, 129.9, 122.9, 120.0, 117.1, 114.0, 108.4, 105.8, 99.9, 55.8, 53.1, 50.6, 18.7. ESI-MS (*m/z*): [M + H]^+^ = 486.1 (Calcd: 486.24). ESI-HRMS: calcd for C_28_H_33_N_5_O_3_ (M + H)^+^ 486.2505, found 486.2502. HPLC analysis: MeOH-H_2_O (80: 20), 7.36 min, 96.0% purity.

**4-((*3R,5S*)-3,5-dimethylpiperazin-1-yl)-*N*-(6–(3-methoxyphenyl)-1*H*-indazol-3-yl)benzamide (9 b)** White solid: 24.8 mg, yield 30%; Mp 224.8–227.4 °C. 1H NMR (400 MHz, DMSO-*d*_6_) δ: 12.81 (brs, 1H), 10.52 (brs, 1H), 7.98 (d, *J* = 8.9 Hz, 2H), 7.78 (d, *J* = 8.1 Hz, 1H), 7.68 (s, 1H), 7.37–7.44 (m, 2H), 7.30 (d, *J* = 7.9 Hz, 1H), 7.26 (s, 1H), 7.03 (d, *J* = 8.2 Hz, 2H), 6.97 (dd, *J* = 8.1, 2.2 Hz, 1H), 3.85 (s, 3H), 3.79–3.81 (m, 2H), 2.85–2.93 (m, 2H), 2.28–2.34 (m, 2H), 1.09 (s, 3H), 1.07 (s, 3H). ^13^ C NMR (100 MHz, DMSO-*d6*) δ: 165.7, 160.2, 153.1, 142.5, 142.2, 141.0, 139.0, 130.6, 129.9, 123.0, 119.9, 117.0, 114.1, 113.6, 113.1, 108.3, 55.6, 52.8, 50.7, 18.4. ESI-MS (*m/z*): [M + H]^+^ = 456.1 (Calcd: 456.23). ESI-HRMS: calcd for C_27_H_31_N_5_O_2_ (M + H)^+^ 456.2400, found 456.2390. HPLC analysis: MeOH-H_2_O (80: 20), 7.27 min, 95.9% purity.

***N*-(6–(3-methoxyphenyl)-1*H*-indazol-3-yl)-4–(4-methylpiperazin-1-yl)benzamide (9c)** White solid: 40.2 mg, yield 45%; Mp 224.8–227.4 °C. 1H NMR (400 MHz, DMSO-*d*_6_) δ: 12.82 (brs, 1H), 10.55 (brs, 1H), 8.00 (d, *J* = 8.6 Hz, 2H), 7.80 (d, *J* = 8.8 Hz, 1H), 7.65 (s, 1H), 7.35–7.45 (m, 2H), 7.29 (d, *J* = 8.0 Hz, 1H), 7.25 (d, *J* = 2.0 Hz, 1H), 7.05 (d, *J* = 8.8 Hz, 2H), 6.95 (dd, *J* = 8.2, 1.9 Hz, 1H), 3.83 (s, 3H), 2.48–2.55 (m, 4H), 2.39 (q, *J* = 7.1 Hz, 2H), 1.05 (t, *J* = 7.2 Hz, 3H). ^13^ C NMR (100 MHz, DMSO-*d6*) δ: 165.6, 160.2, 153.7, 142.6, 141.1, 139.0, 130.5, 129.8, 123.1, 122.9, 120.0, 119.9, 117.0, 113.9, 113.6, 113.1, 108.3, 56.2, 52.6, 52.1, 46.2. ESI-MS (*m/z*): [M + H]^+^ = 442.2 (Calcd: 442.21). ESI-HRMS: calcd for C_26_H_28_N_5_O_2_ (M + H)^+^ 442.2198, found 442.2190. HPLC analysis: MeOH-H_2_O (80: 20), 7.38 min, 96.5% purity.

***N*-(6–(3-ethoxyphenyl)-1*H*-indazol-3-yl)-4–(4-ethylpiperazin-1-yl)benzamide (9e)** White solid: 22.7 mg, yield 39%; Mp 223.6–226.0 °C. ^1^H NMR (400 MHz, DMSO-*d*_6_) δ: 12.80 (brs, 1H), 10.54 (brs, 1H), 8.00 (d, *J* = 8.9 Hz, 2H), 7.78 (d, *J* = 8.5 Hz, 1H), 7.66 (s, 1H), 7.35–7.43 (m, 2H), 7.29 (d, *J* = 7.8 Hz, 1H), 7.24 (s, 1H), 7.03 (d, *J* = 8.9 Hz, 2H), 6.96 (dd, *J* = 8.1, 1.9 Hz, 1H), 4.13 (q, *J* = 6.9 Hz, 2H), 3.26–3.40 (m, 4H), 2.51–2.62 (m, 4H), 2.34–2.45 (m, 2H), 1.37 (t, *J* = 8.0 Hz, 3H), 1.06 (t, *J* = 7.1 Hz, 3H). ^13 ^C NMR (100 MHz, DMSO-*d_6_*) δ: 165.6, 159.5, 153.6, 142.6, 142.2, 141.1, 139.0, 130.5, 129.8, 123.1, 123.0, 119.9, 117.0, 114.0, 113.9, 113.6, 108.3, 63.6, 52.5, 52.1, 47.4, 15.2, 12.3. ESI-MS (*m/z*): [M + H]^+^ = 470.2 (Calcd: 470.25). ESI-HRMS: calcd for C_28_H_33_N_5_O_2_ (M + H)^+^ 470.2556, found 470.2550. HPLC analysis: MeOH-H_2_O (80: 20), 7.42 min, 96.9% purity.

**4–(4-ethylpiperazin-1-yl)-*N*-(6–(3-*iso*propoxyphenyl)-1*H*-indazol-3-yl)benzamide (9f)** White solid: 21.3 mg, yield 39%; Mp 227.9–231.7 °C. 1H NMR (400 MHz, DMSO-*d*_6_) δ: 12.77 (brs, 1H), 10.52 (brs, 1H), 8.00 (d, *J* = 8.7 Hz, 2H), 7.78 (d, *J* = 8.5 Hz, 1H), 7.65 (s, 1H), 7.35–7.41 (m, 2H), 7.27 (d, *J* = 7.7 Hz, 1H), 7.22 (s, 1H), 7.03 (d, *J* = 8.0 Hz, 2H), 6.92 (dd, *J* = 8.3, 1.9 Hz, 1H), 4.72–4.78 (m, 1H), 3.27–3.38 (m, 4H), 2.40–2.58 (m, 4H), 2.34 (q, *J* = 7.1 Hz, 2H), 1.32 (s, 3H), 1.31 (s, 3H), 1.06 (t, *J* = 7.1 Hz, 3H). ^13 ^C NMR (100 MHz, DMSO-*d_6_*) δ: 166.6, 158.4, 153.6, 142.7, 142.2, 141.1, 139.0, 130.6, 129.8, 123.1, 119.8, 117.0, 115.0, 114.9, 114.0, 69.7, 52.5, 52.0, 47.3, 22.4, 12.3. ESI-MS (*m/z*): [M + H]^+^ = 484.3 (Calcd: 484.27). ESI-HRMS: calcd for C_29_H_35_N_5_O_2_ (M + H)^+^ 484.2713, found 484.2706. HPLC analysis: MeOH-H_2_O (80: 20), 7.22 min, 97.1% purity.

***N*-(6–(3-(*sec*-butoxy)phenyl)-1*H*-indazol-3-yl)-4–(4-ethylpiperazin-1-yl)benzamide (9 g)** White solid: 23.9 mg, yield 47%; Mp 225.9–290.0 °C. 1H NMR (400 MHz, DMSO-*d*_6_) δ: 12.79 (brs, 1H), 10.54 (brs, 1H), 8.00 (d, *J* = 8.9 Hz, 2H), 7.78 (d, *J* = 8.5 Hz, 1H), 7.67 (s, 1H), 7.37–7.42 (m, 2H), 7.29 (d, *J* = 7.9 Hz, 1H), 7.24–7.26 (m, 1H), 7.03 (d, *J* = 9.0 Hz, 2H), 6.96 (dd, *J* = 8.1, 1.8 Hz, 1H), 3.86 (s, 1H), 3.84 (s, 1H), 3.26–3.38 (m, 4H), 2.51–2.60 (m, 4H), 2.40–2.46 (m, 2H), 2.03–2.09 (m, 1H), 1.06 (t, *J* = 7.1 Hz, 3H), 1.03 (s, 3H), 1.01 (s, 3H). ^13 ^C NMR (100 MHz, DMSO-*d_6_*) δ: 166.6, 159.8, 153.6, 142.6, 142.2, 141.1, 139.0, 130.5, 129.8, 123.1, 119.9, 117.0, 114.0, 113.6, 108.3, 74.3, 52.5, 52.0, 47.3, 31.2, 28.3, 19.6, 12.3. ESI-MS (*m/z*): [M + H]^+^ = 498.3 (Calcd: 498.29). ESI-HRMS: calcd for C_30_H_37_N_5_O_2_ (M + H)^+^ 498.2869, found 498.2875. HPLC analysis: MeOH-H_2_O (80: 20), 7.30 min, 97.0% purity.

**methyl 3–(3-(4–(4-ethylpiperazin-1-yl)benzamido)-1*H*-indazol-6-yl)benzoate (9 h)** White solid: 27.5 mg, yield 45%; Mp 228.7–233.4 °C. 1H NMR (400 MHz, DMSO-*d*_6_) δ 12.91 (brs, 1H), 10.61 (brs, 1H), 8.27 (s, 1H), 7.98–8.06 (m, 4H), 7.84 (d, *J* = 8.5 Hz, 1H), 7.74 (s, 1H), 7.65–7.69 (m, 1H), 7.42 (d, *J* = 8.6 Hz, 1H), 7.08 (d, *J* = 8.5 Hz, 2H), 3.91 (s, 3H), 3.42–3.48 (m, 4H), 2.80–2.90 (m, 4H), 2.48–2.52 (q, *J* = 7.6 Hz, 2H), 1.19 (t, *J* = 7.1 Hz, 3H). ^13 ^C NMR (100 MHz, DMSO-*d_6_*) δ: 166.7, 165.6, 153.0, 142.2, 141.5, 141.1, 137.9, 132.5, 130.9, 130.1, 129.9, 128.6, 128.0, 123.4, 119.6, 117.2, 114.3, 108.5, 52.8, 51.6, 46.1, 29.5, 10.8. ESI-MS (*m/z*): [M + H]^+^ = 484.3 (Calcd: 484.24). ESI-HRMS: calcd for C_28_H_31_N_5_O_3_ (M + H)^+^ 484.2349, found 484.2358. HPLC analysis: MeOH-H_2_O (80: 20), 7.05 min, 96.5% purity.

**4–(4-ethylpiperazin-1-yl)-*N*-(6–(3-methoxy-4-methylphenyl)-1*H*-indazol-3-yl) benzamide (9i)** White solid: 27.7 mg, yield 41%; Mp 227.3–231.2 °C. ^1^H NMR (400 MHz, DMSO-*d*_6_) δ 12.83 (brs, 1H), 10.54 (brs, 1H), 8.01 (d, *J* = 8.7 Hz, 2H), 7.75 (d, *J* = 8.5 Hz, 1H), 7.60 (s, 1H), 7.54 (s, 2H), 7.33 (dd, *J* = 8.6, 1.1 Hz, 1H), 7.05 (d, *J* = 9.1 Hz, 3H), 3.84 (s, 3H), 3.33–3.38 (m, 4H), 2.59–2.64 (m, 4H), 2.46 (q, *J* = 7.3 Hz, 2H), 2.25 (s, 3H), 1.09 (t, *J* = 7.7 Hz, 3H). ^13 ^C NMR (100 MHz, DMSO-*d_6_*) δ: 165.5, 157.6, 142.4, 141.0, 138.9, 132.9, 129.9, 126.5, 126.1, 122.9, 119.5, 116.4, 114.1, 111.2, 107.3, 56.5, 55.9, 51.8, 46.6, 19.0, 16.6. ESI-MS (*m/z*): [M + H]^+^ = 470.3 (Calcd: 470.26). ESI-HRMS: calcd for C_28_H_33_N_5_O_2_ (M + H)^+^ 470.2556, found 470.2551. HPLC analysis: MeOH-H_2_O (80: 20), 7.14 min, 96.3% purity.

***N*-(6–(3,5-bis(trifluoromethyl)phenyl)-1*H*-indazol-3-yl)-4–(4-ethylpiperazin-1-yl)benzamide (9j)** White solid: 33.6 mg, yield 44%; Mp 227.5–213.3 °C. ^1^H NMR (400 MHz, DMSO-*d*_6_) δ: 12.95 (brs, 1H), 10.59 (brs, 1H), 8.42 (s, 2H), 8.13 (s, 1H), 8.00 (d, *J* = 8.9 Hz, 2H), 7.94 (s, 1H), 7.87 (d, *J* = 8.6 Hz, 1H), 7.54 (d, *J* = 8.2 Hz, 1H), 7.04 (d, *J* = 8.0 Hz, 2H), 3.27–3.38 (m, 4H), 2.44–2.52 (m, 4H), 2.40 (q, *J* = 7.1 Hz, 2H), 1.05 (t, *J* = 7.2 Hz, 3H). ^13 ^C NMR (100 MHz, DMSO-*d6*) δ: 165.6, 153.6, 143.7, 141.9, 141.3, 135.7, 131.5, 131.2, 129.9, 128.4, 125.0, 123.6, 122.9, 122.8, 119.7, 117.6, 113.9, 109.7, 52.6, 52.1, 47.4, 12.4. ESI-MS (*m/z*): [M + H]^+^ = 562.2 (Calcd: 562.20). ESI-HRMS: calcd for C_28_H_27_F_6_N_5_O (M + H)^+^ 562.2042, found 562.2049. HPLC analysis: MeOH-H_2_O (80: 20), 7.08 min, 95.9% purity.

***N*-(6–(3,5-dichlorophenyl)-1*H*-indazol-3-yl)-4–(4-ethylpiperazin-1-yl)benzamide (9k)** White solid: 28.9 mg, yield 41%; Mp 226.4–228.7 °C. ^1^H NMR (400 MHz, DMSO-*d*_6_) δ 12.90 (brs, 1H), 10.58 (brs, 1H), 7.99 (t, *J* = 9.4 Hz, 2H), 7.81 (dd, *J* = 10.2, 5.0 Hz, 3H), 7.67–7.73 (m, 1H), 7.63 (t, *J* = 1.7 Hz, 1H), 7.16–7.47 (m, 1H), 7.03 (dd, *J* = 8.8, 4.5 Hz, 2H), 3.34 (s, 4H), 2.46–2.67 (m, 4H), 2.41 (s, 2H), 1.06 (t, *J* = 7.0 Hz, 3H). ^13 ^C NMR (100 MHz, DMSO-*d_6_*) δ: 165.5, 153.6, 144.6, 141.9, 136.0, 135.1, 129.8, 127.3, 126.4, 124.7, 123.5, 123.1, 120.2, 119.5, 117.5, 113.9, 113.1, 109.1, 52.5, 52.0, 47.3, 12.3. ESI-MS (*m/z*): [M + H]^+^ = 494.2 (Calcd: 494.15). ESI-HRMS: calcd for C_26_H_27_Cl_2_N_5_O (M + H)^+^ 494.1514, found 494.1508. HPLC analysis: MeOH-H_2_O (80: 20), 7.23 min, 96.5% purity.

***N*-(6–(2,5-dichlorophenyl)-1*H*-indazol-3-yl)-4–(4-ethylpiperazin-1-yl)benzamide (9 l)** White solid: 30.3 mg, yield 39%; Mp 231.5–234.6 °C. ^1^H NMR (400 MHz, DMSO-*d*_6_) δ 12.93 (brs, 1H), 10.59 (brs, 1H), 8.01 (d, *J* = 8.8 Hz, 2H), 7.79 (d, *J* = 8.5 Hz, 1H), 7.64 (d, *J* = 8.6 Hz, 1H), 7.58 (d, *J* = 2.5 Hz, 1H), 7.52 (dd, *J* = 8.3, 2.8 Hz, 2H), 7.09–7.15 (m, 1H), 7.05 (d, *J* = 8.8 Hz, 2H), 3.36–3.40 (m, 4H), 2.61–2.68 (m, 4H), 2.46–2.50 (q, *J* = 7.2 Hz, 2H), 1.10 (t, *J* = 6.8 Hz, 3H). ^13 ^C NMR (100 MHz, DMSO-*d_6_*) δ: 165.7, 153.4, 142.3, 141.3, 141.1, 136.0, 132.4, 131.9, 131.6, 130.9, 128.9, 129.5, 123.2, 122.5, 121.6, 117.1, 114.1, 111.2, 52.1, 51.9, 46.8, 11.6. ESI-MS (*m/z*): [M + H]^+^ = 494.2 (Calcd: 494.15). ESI-HRMS: calcd for C_26_H_27_Cl_2_N_5_O (M + H)^+^ 494.1514, found 494.1510. HPLC analysis: MeOH-H_2_O (80: 20), 7.36 min, 96.7% purity.

***N*-(6–(2,3-dichlorophenyl)-1*H*-indazol-3-yl)-4–(4-ethylpiperazin-1-yl)benzamide (9 m)** White solid: 22.1 mg, yield 36%; Mp 220.1–222.4 °C. ^1^H NMR (400 MHz, DMSO-*d*_6_) δ: 12.89 (brs, 1H), 10.58 (brs, 1H), 8.00 (d, *J* = 8.4 Hz, 2H), 7.78 (d, *J* = 8.0 Hz, 1H), 7.70–7.72 (m, 1H), 7.46–7.50 (m, 3H), 7.10 (d, *J* = 7.8 Hz, 1H), 7.04 (d, *J* = 8.4 Hz, 2H), 3.26–3.40 (m, 4H), 2.51–2.55 (m, 4H), 2.42 (q, *J* = 7.1 Hz, 2H), 1.05 (t, *J* = 7.2 Hz, 3H). ^13 ^C NMR (100 MHz, DMSO-*d_6_*) δ: 165.6, 153.5, 143.1, 141.3, 141.2, 137.1, 132.8, 130.8, 130.4, 130.2, 129.8, 128.8, 123.0, 122.5, 121.6, 117.0, 114.0, 111.0, 52.4, 52.0, 47.2, 12.2. ESI-MS (*m/z*): [M + H]^+^ = 494.2 (Calcd: 494.15). ESI-HRMS: calcd for C_26_H_27_Cl_2_N_5_O (M + H)^+^ 494.1514, found 494.1509. HPLC analysis: MeOH-H_2_O (80: 20), 6.88 min, 95.5% purity.

***N*-(6–(3,4-dichlorophenyl)-1*H*-indazol-3-yl)-4–(4-ethylpiperazin-1-yl)benzamide (9n)** White solid: 21.8 mg, yield 38%; Mp 224.9–228.0 °C. ^1^H NMR (400 MHz, DMSO-*d*_6_) δ: 12.91 (brs, 1H), 10.57 (brs, 1H), 7.99–8.03 (m, 3H), 7.73–7.81 (m, 3H), 7.53–7.60 (m, 1H), 7.40–7.47 (m, 1H), 7.04 (d, *J* = 8.4 Hz, 2H), 3.26–3.40 (m, 4H), 2.51–2.55 (m, 4H), 2.41 (q, *J* = 7.1 Hz, 2H), 1.06 (t, *J* = 7.2 Hz, 3H). ^13 ^C NMR (100 MHz, DMSO-*d_6_*) δ: 165.5, 153.6, 142.0, 141.7, 141.2, 136.3, 132.2, 131.5, 130.7, 129.8, 129.4, 127.9, 123.5, 122.9, 119.4, 117.3, 113.9, 108.7, 52.6, 52.1, 47.4, 12.4. ESI-MS (*m/z*): [M + H]^+^ = 494.2 (Calcd: 494.15). ESI-HRMS: calcd for C_26_H_27_Cl_2_N_5_O (M + H)^+^ 494.1514, found 494.1514. HPLC analysis: MeOH-H_2_O (80: 20), 7.05 min, 96.5% purity.

**4–(4-ethylpiperazin-1-yl)-*N*-(6–(3-fluoro-5-methoxyphenyl)-1*H*-indazol-3-yl)benzamide (9o)** White solid: 22.1 mg, yield 34%; Mp 225.5–227.0 °C. ^1^H NMR (400 MHz, DMSO-*d*_6_) δ: 12.86 (brs, 1H), 10.55 (brs, 1H), 8.00 (d, *J* = 8.9 Hz, 2H), 7.80 (d, *J* = 8.5 Hz, 1H), 7.72 (s, 1H), 7.40 (dd, *J* = 8.6, 1.3 Hz, 1H), 7.12–7.21 (m, 2H), 7.03 (d, *J* = 9.0 Hz, 2H), 6.85–6.89 (m, 1H), 3.87 (s, 3H), 3.26–3.41 (m, 4H), 2.51–2.57 (m, 4H), 2.39 (q, *J* = 7.1 Hz, 2H), 1.05 (t, *J* = 7.2 Hz, 3H). ^13 ^C NMR (100 MHz, DMSO-*d_6_*) δ: 165.0, 162.2, 161.1, 143.5, 141.5, 140.7, 137.2, 129.3, 122.8, 122.4, 119.2, 116.8, 113.4, 109.1, 108.1, 106.2, 106.0, 55.7, 52.1, 51.6, 46.9, 11.9. HRMS: calcd for C_27_H_30_FN_5_O_2_ (M + H)^+^ 474.2305, found 474.2296. HPLC analysis: MeOH-H_2_O (80: 20), 7.24 min, 96.7% purity.

***N*-(6–(2-chloro-4-(trifluoromethyl)phenyl)-1*H*-indazol-3-yl)-4–(4-ethylpiperazin-1-yl)benzamide (9p)** White solid: 19.9 mg, yield 35%; Mp 227.9–232.0 °C. ^1^H NMR (400 MHz, DMSO-*d*_6_) δ 12.92 (brs, 1H), 10.58 (brs, 1H), 7.97–8.04 (m, 3H), 7.80–7.85 (, 2H), 7.74 (d, *J* = 8.0 Hz, 1H), 7.55 (s, 1H), 7.14 (dd, *J* = 8.5, 1.2 Hz, 1H), 7.03 (d, *J* = 8.9 Hz, 2H), 3.33–3.40 (m, 4H), 2.48–2.52 (m, 4H), 2.45 (s, 2H), 1.06 (t, *J* = 7.1 Hz, 3H). ^13 ^C NMR (100 MHz, DMSO-*d_6_*) δ: 165.7, 153.5, 144.8, 141.3, 136.0, 133.2, 133.0, 129.9, 127.2, 124.8, 123.0, 122.7, 121.4, 117.2, 114.0, 111.2, 52.4, 52.0, 47.1, 12.1. ESI-MS (*m/z*): [M + H]^+^ = 528.1 (Calcd: 528.17). ESI-HRMS: calcd for C_27_H_27_ClF_3_N_5_O (M + H)^+^ 528.1778, found 528.1777. HPLC analysis: MeOH-H_2_O (80: 20), 7.18 min, 96.9% purity.

***N*-(6–(2,6-dimethylphenyl)-1*H*-indazol-3-yl)-4–(4-ethylpiperazin-1-yl)benzamide (9q)** White solid: 30.9 mg, yield 40%; Mp 235.7–239.2 °C. ^1^H NMR (400 MHz, DMSO-*d*_6_) δ 12.75 (brs, 1H), 10.56 (brs, 1H), 8.02 (d, *J* = 8.7 Hz, 2H), 7.77 (d, *J* = 8.3 Hz, 1H), 7.18 (d, *J* = 8.4 Hz, 2H), 7.14 (d, *J* = 8.2 Hz, 2H), 7.07 (d, *J* = 8.6 Hz, 2H), 6.82 (d, *J* = 8.3 Hz, 1H), 3.37–3.43 (m, 4H), 2.54–2.68 (m, 4H), 2.35 (q, *J* = 7.2 Hz, 2H), 2.01 (s, 6H), 1.14 (t, *J* = 7.0 Hz, 3H). ^13 ^C NMR (100 MHz, DMSO-*d_6_*) δ: 165.6, 153.0, 142.0, 141.1, 139.1, 135.8, 129.9, 127.8, 127.5, 122.8, 121.5, 116.5, 114.3, 110.2, 51.7, 51.6, 46.4, 21.0, 12.2. ESI-MS (*m/z*): [M + H]^+^ = 454.3 (Calcd: 454.26). ESI-HRMS: calcd for C_26_H_28_ClN_5_O (M + H)^+^ 454.2607, found 454.2606. HPLC analysis: MeOH-H_2_O (80: 20), 7.30 min, 95.7% purity.

**4–(4-ethylpiperazin-1-yl)-*N*-(6–(2-(trifluoromethoxy)phenyl)-1*H*-indazol-3-yl)benzamide (9r)** White solid: 28.2 mg, yield 38%; Mp 230.9–233.5 °C. ^1^H NMR (400 MHz, DMSO-*d*_6_) δ: 12.87 (brs, 1H), 10.57 (brs, 1H), 8.00 (d, *J* = 8.4 Hz, 2H), 7.79 (d, *J* = 7.8 Hz, 1H), 7.62–7.64 (m, 1H), 7.53–7.57 (m, 4H), 7.16 (d, *J* = 8.0 Hz, 1H), 7.04 (d, *J* = 8.8 Hz, 2H), 3.28–3.43 (m, 4H), 2.47–2.53 (m, 4H), 2.41 (q, *J* = 7.1 Hz, 2H), 1.06 (t, *J* = 7.2 Hz, 3H). ^13 ^C NMR (100 MHz, DMSO-*d_6_*) δ: 165.6, 153.6, 145.9, 141.6, 141.2, 135.5, 134.7, 132.4, 129.9, 128.5, 123.0, 122.6, 121.5, 117.0, 113.9, 110.8, 52.5, 52.0, 47.4, 12.3. ESI-MS (*m/z*): [M + H]^+^ = 510.2 (Calcd: 510.21). ESI-HRMS: calcd for C_27_H_28_F_3_N_5_O_2_ (M + H)^+^ 510.2117, found 510.2119. HPLC analysis: MeOH-H_2_O (80: 20), 7.18 min, 96.9% purity.

***N*-(6–(4-cyanophenyl)-1*H*-indazol-3-yl)-4–(4-ethylpiperazin-1-yl)benzamide (9 s)** White solid: 15.8 mg, yield 38%; Mp 226.4–230.0 °C. ^1^H NMR (400 MHz, DMSO-*d*_6_) δ 12.94 (brs, 1H), 10.52 (brs, 1H), 7.93–8.08 (m, 6H), 7.85 (d, *J* = 8.5 Hz, 1H), 7.78 (s, 1H), 7.44 (d, *J* = 8.6 Hz, 1H), 7.03 (d, *J* = 8.6 Hz, 2H), 3.28–3.39 (m, 4H), 2.51–2.59 (m, 4H), 2.40 (q, *J* = 6.7 Hz, 2H), 1.06 (t, *J* = 7.1 Hz, 3H). ^13 ^C NMR (100 MHz, DMSO-*d_6_*) δ: 165.6, 153.7, 145.6, 142.1, 141.2, 137.1, 133.3, 129.9, 128.5, 123.6, 122.9, 119.6, 117.5, 113.9, 110.5, 109.1, 52.5, 52.1, 47.4, 12.3. ESI-MS (*m/z*): [M + H]^+^ = 451.2 (Calcd: 451.22). ESI-HRMS: calcd for C_27_H_28_N_6_O (M + H)^+^ 451.2246, found 451.2238. HPLC analysis: MeOH-H_2_O (80: 20), 7.40 min, 96.3% purity.

**4–(4-ethylpiperazin-1-yl)-*N*-(6–(1-methyl-1*H*-pyrazol-3-yl)-1*H*-indazol-3-yl) benzamide (9t)** White solid: 18.7 mg, yield 35%; Mp 221.6–224.2 °C. ^1^H NMR (400 MHz, DMSO-*d*_6_) δ 12.67 (brs, 1H), 10.46 (brs, 1H), 8.23 (s, 1H), 7.98 (d, *J* = 8.8 Hz, 2H), 7.94 (s, 1H), 7.68 (d, *J* = 8.5 Hz, 1H), 7.57 (s, 1H), 7.30 (d, *J* = 8.5 Hz, 1H), 7.02 (d, *J* = 8.8 Hz, 2H), 3.89 (s, 3H), 3.37–3.45 (m, 4H), 2.52–2.60 (m, 4H), 2.41 (q, *J* = 6.7 Hz, 2H), 1.06 (t, *J* = 7.2 Hz, 3H). ^13 ^C NMR (100 MHz, DMSO-*d_6_*) δ: 165.6, 153.6, 142.4, 141.1, 136.8, 131.3, 129.8, 128.7, 123.0, 122.7, 118.6, 116.1, 113.9, 105.5, 52.5, 52.0, 47.3, 19.0, 12.3. ESI-MS (*m/z*): [M + H]^+^ = 430.2 (Calcd: 430.24). ESI-HRMS: calcd for C_24_H_29_N_7_O (M + H)^+^ 430.2355, found 430.2357. HPLC analysis: MeOH-H_2_O (80: 20), 7.11 min, 95.5% purity.

**4–(4-(dimethylamino)piperidin-1-yl)-*N*-(6–(3-fluoro-5-methoxyphenyl)-1*H*-indazol-3-yl)benzamide (9 u)** White solid: 14.3 mg, yield 46%; Mp 223.8–227.4 °C. ^1^H NMR (400 MHz, DMSO-*d*_6_) δ 12.87 (brs, 1H), 10.54 (brs, 1H), 7.99 (d, *J* = 8.7 Hz, 2H), 7.79 (d, *J* = 8.5 Hz, 1H), 7.72 (s, 1H), 7.40 (d, *J* = 8.8 Hz, 1H), 7.11–7.22 (m, 2H), 7.04 (d, *J* = 8.8 Hz, 2H), 6.87 (d, *J* = 10.9 Hz, 1H), 3.98 (d, *J* = 12.2 Hz, 2H), 3.87 (s, 3H), 2.82–2.90 (m, 2H), 2.59 (s, 1H), 2.35 (s, 6H), 1.92 (s, 2H), 1.51 (d, *J* = 10.8 Hz, 2H). ^13 ^C NMR (100 MHz, DMSO-*d6*) δ: 165.5, 164.8, 162.9, 161.6, 153.2, 143.9, 142.0, 141.2, 137.7, 129.9, 123.3, 122.5, 119.6, 117.3, 114.1, 109.6, 108.6, 106.7, 100.9, 62.1, 56.2, 46.9, 41.3, 27.4, 21.6. ESI-MS (*m/z*): [M + H]^+^ = 488.3 (Calcd: 488.26). HRMS: calcd for C_27_H_30_FN_5_O_2_ (M + H)^+^ 488.2462, found 488.2459. HPLC analysis: MeOH-H_2_O (80: 20), 7.22 min, 97.5% purity.

**4-((*3R,5S*)-3,5-dimethylpiperazin-1-yl)-*N*-(6–(3-fluoro-5-methoxyphenyl)-1*H*-indazol-3-yl)benzamide (9v)** White solid: 26.5 mg, yield 38%; Mp 229.6–233.4 °C. ^1^H NMR (400 MHz, DMSO-*d*_6_) δ 12.87 (brs, 1H), 10.56 (brs, 1H), 7.99 (d, *J* = 8.8 Hz, 2H), 7.79 (d, *J* = 8.6 Hz, 1H), 7.72 (s, 1H), 7.40 (d, *J* = 8.5 Hz, 1H), 7.14–7.18 (m, 2H), 7.04 (d, *J* = 9.0 Hz, 2H), 6.87 (d, *J* = 10.9 Hz, 1H), 4.47 (brs, 1H), 3.87 (s, 3H), 3.82 (s, 2H), 2.96 (s, 2H), 2.36 (d, *J* = 10.7 Hz, 2H), 1.11 (d, *J* = 6.1 Hz, 6H). ^13 ^C NMR (100 MHz, DMSO-*d6*) δ: 165.5, 164.8, 162.9, 161.6, 153.2, 143.9, 142.0, 141.1, 137.7, 129.9, 126.7, 123.3, 119.6, 117.3, 113.9, 109.6, 108.6, 106.7, 100.9, 63.3, 56.2, 53.3, 50.6, 19.0. ESI-MS (*m/z*): [M + H]^+^ = 474.2 (Calcd: 474.23). ESI-HRMS: calcd for C_27_H_30_FN_5_O_2_ (M + H)^+^ 474.2305, found 474.2290. HPLC analysis: MeOH-H_2_O (80: 20), 7.11 min, 95.5% purity.

***N*-(6–(3-fluoro-5-methoxyphenyl)-1*H*-indazol-3-yl)-4–(4-methyl-1,4-diazepan-1-yl)benzamide (9w)** White solid: 30.1 mg, yield 33%; Mp 230.4–234.6 °C. ^1^H NMR (400 MHz, DMSO-*d*_6_) δ 12.83 (brs, 1H), 10.44 (brs, 1H), 7.97 (d, *J* = 8.4 Hz, 2H), 7.79 (d, *J* = 8.0 Hz, 1H), 7.71 (s, 1H), 7.39 (d, *J* = 8.1 Hz, 1H), 7.14–7.18 (m, *2*H), 6.85–6.89 (m, 1H), 6.80 (d, *J* = 7.9 Hz, 2H), 3.87 (s, 7H), 3.63–3.65 (m, 2H), 3.53 (t, *J* = 6.8 Hz, 2H), 2.70–2.78 (m, 2H), 2.55–2.62 (m, 2H), 2.48–2.51 (m, 4H), 2.36 (s, 3H). ^13 ^C NMR (100 MHz, DMSO-*d_6_*) δ: 165.6, 164.8, 162.9, 161.6, 151.7, 144.0, 142.0, 137.6, 130.16, 123.3, 120.2, 119.6, 117.3, 110.8, 109.6, 108.6, 106.7, 101.1, 57.2, 56.6, 56.2, 48.1, 46.1, 26.8. ESI-MS (*m/z*): [M + H]^+^ = 474.0(Calcd: 473.22). ESI-HRMS: calcd for C_27_H_30_FN_5_O_2_ (M + H)^+^ 474.2305, found 474.2297. HPLC analysis: MeOH-H_2_O (80: 20), 7.15 min, 96.2% purity.

***N*-(6–(3-fluoro-5-methoxyphenyl)-1*H*-indazol-3-yl)-4-((4-methylpiperazin-1-yl)methyl)benzamide (9x)** White solid: 20.1 mg, yield 44%; Mp 240.3–242.5 °C. ^1^H NMR (400 MHz, DMSO-*d*_6_) δ: 13.01 (brs, 1H), 10.84 (brs, 1H), 8.06 (d, *J* = 8.2 Hz, 2H), 7.80 (d, *J* = 8.2 Hz, 1H), 7.74 (s, 1H), 7.46 (d, *J* = 8.0 Hz, 2H), 7.41 (dd, *J* = 8.0, 1.4 Hz, 1H), 7.14–7.18 (m, 2H), 6.85–6.89 (m, 1H), 3.87 (s, 1H), 3.55 (s, 2H), 2.33–2.46 (m, 8H), 2.16 (s, 3H). ^13 ^C NMR (100 MHz, DMSO-*d_6_*) δ: 165.9, 164.8, 162.9, 161.6, 144.0, 143.0, 142.0, 140.6, 137.7, 132.9, 130.1, 129.1, 128.4, 123.0, 119.8, 117.1, 109.6, 108.7, 106.7, 100.9, 62.1, 56.2, 55.2, 53.1, 46.2. ESI-MS (*m/z*): [M + H]^+^ = 474.2 (Calcd: 474.23). ESI-HRMS: calcd for C_27_H_30_FN_5_O_2_ (M + H)^+^ 474.2305, found 474.2298. HPLC analysis: MeOH-H_2_O (80: 20), 7.11 min, 95.5% purity.

***N*-(6–(3-fluoro-5-methoxyphenyl)-1*H*-indazol-3-yl)-4–(4-methylpiperazin-1-yl)benzamide (9 y)** White solid: 17.1 mg, yield 30%; Mp 237.4–241.6 °C. ^1^H NMR (400 MHz, DMSO-*d*_6_) δ: 13.01 (brs, 1H), 10.84 (brs, 1H), 8.06 (d, *J* = 8.2 Hz, 2H), 7.80 (d, *J* = 8.2 Hz, 1H), 7.74 (s, 1H), 7.46 (d, *J* = 8.0 Hz, 2H), 7.41 (dd, *J* = 8.0, 1.4 Hz, 1H), 7.14–7.18 (m, 2H), 6.85–6.89 (m, 1H), 3.87 (s, 1H), 3.24–3.39 (s, 4H), 2.44–2.52 (s, 4H), 2.16 (s, 3H). ^13 ^C NMR (100 MHz, DMSO-*d_6_*) δ: 165.9, 164.7, 163.1, 161.6, 144.0, 143.0, 142.0, 140.7, 137.7, 132.9, 129.1, 128.4, 123.0, 119.8, 117.2, 109.6, 108.7, 106.7, 101.1, 62.1, 56.2, 55.2, 53.1, 46.2. ESI-MS (*m/z*): [M + H]^+^ = 460.2 (Calcd: 460.21). ESI-HRMS: calcd for C_27_H_30_FN_5_O_2_ (M + H)^+^ 460.2149, found 460.2155. HPLC analysis: MeOH-H_2_O (80: 20), 7.42 min, 96.4% purity.

***N*-(6–(3-fluoro-5-methoxyphenyl)-1*H*-indazol-3-yl)-5–(4-methylpiperazin-1-yl)pyrazine-2-carboxamide (9z)** White solid: 19.8 mg, yield 35%; Mp 237.7–240.3 °C. ^1^H NMR (400 MHz, DMSO-*d*_6_) δ: 12.95 (brs, 1H), 10.42 (brs, 1H), 8.80 (s, 1H), 8.44 (s, 1H), 7.94 (d, *J* = 8.4 Hz, 1H), 7.74 (s, 1H), 7.42 (d, *J* = 8.3 Hz, 1H), 7.05–7.25 (m, 2H), 6.87 (d, *J* = 10.9 Hz, 1H), 3.87 (s, 7H), 2.70 (s, 4H), 2.42 (s, 3H). ESI-MS (*m/z*): [M + H]^+^=462.2 (Calcd: 462.21). ^13^ C NMR (100 MHz, DMSO-*d6*) δ: 164.8, 162.8, 161.6, 155.6, 142.8 142.1, 140.1, 137.8, 132.4, 130.1, 129.5, 123.2, 119.8, 116.7 109.6, 108.6, 106.7, 101.0, 56.2, 54.5, 46.0, 44.2. ESI-MS (*m/z*): [M + H]^+^ = 462.2 (Calcd: 462.20). HRMS: calcd for C_24_H_25_FN_7_O_2_ (M + H)^+^ 462.2054, found 462.2038. HPLC analysis: MeOH-H_2_O (80: 20), 7.11 min, 95.5% purity.

### *General method for preparation of compounds 12a-12d* (exemplified by 12a)

***N*-(6–(3-methoxyphenyl)benzo[d]thiazol-2-yl)-4–(4-methylpiperazin-1-yl) benzamide (12a)** white solid: 73.7 mg, yield 53%. Mp 230.5–233.2 °C. ^1^H NMR (500 MHz, DMSO-*d_6_*) δ: 12.57 (brs, 1H), 8.34 (s, 1H), 8.07 (d, *J* = 8.8 Hz, 2H), 7.81 (d, *J* = 8.4 Hz, 1H), 7.76 (d, *J* = 8.3 Hz, 1H), 7.40 (t, *J* = 7.9 Hz, 1H), 7.31 (d, *J* = 7.7 Hz, 1H), 7.28 (s, 1H), 7.03 (d, *J* = 8.9 Hz, 2H), 6.95 (d, *J* = 8.0 Hz, 1H), 3.85 (s, 3H), 2.44 (s, 4H), 2.23 (s, 3H). ^13 ^C NMR (125 MHz, DMSO-*d*_6_) δ 165.57, 160.24, 160.06, 154.19, 148.63, 141.98, 135.97, 132.96, 130.46, 130.43, 125.64, 120.78, 120.29, 120.19, 119.59, 113.71, 113.34, 112.70, 55.62, 54.77, 46.94, 46.18. HRMS: calcd for C_26_H_27_N_4_O_2_S (M + H)^+^ 459.1776, found 459.1708. HPLC analysis: MeOH-H_2_O (85: 15), 7.50 min, 97.1% purity.

***N*-(6–(3,5-dimethoxyphenyl)benzo[d]thiazol-2-yl)-4–(4-methylpiperazin-1-yl) benzamide (12 b)** White solid 56.7 mg, yield 49%. Mp 234.6–238.2 °C. ^1^H NMR (500 MHz, DMSO-*d*_6_) δ 12.58 (brs, 1H), 8.34 (s, 1H), 8.06 (d, *J* = 8.3 Hz, 2H), 7.73–7.84 (m, 2H), 7.03 (d, *J* = 8.3 Hz, 2H), 6.88 (s, 2H), 6.51 (s, 1H), 3.84 (s, 6H), 2.44 (s, 4H), 2.23 (s, 3H). ^13 ^C NMR (125 MHz, DMSO-*d*_6_) δ 165.57, 161.33, 160.09, 154.19, 148.72, 142.62, 136.02, 132.89, 130.43, 125.67, 120.69, 120.29, 120.25, 113.70, 105.39, 99.68, 55.77, 54.77, 46.94, 46.17. HRMS: calcd for C_27_H_29_N_4_O_3_S (M + H)^+^ 489.1882, found 489.1852. HPLC analysis: MeOH-H_2_O (85: 15), 7.70 min, 96.4% purity.

**4–(4-methylpiperazin-1-yl)-*N*-(6–(3,4,5-trimethoxyphenyl)benzo[d]thiazol-2-yl)benzamide (12c)** White solid 50.5 mg, yield 47%. Mp 236.4–238.3 °C. ^1^H NMR (500 MHz, DMSO-*d*_6_) δ 12.59 (brs, 1H), 8.17 (s, 1H), 8.07 (d, *J* = 8.8 Hz, 2H), 7.83 (d, *J* = 8.4 Hz, 1H), 7.60 (d, *J* = 8.3 Hz, 1H), 7.24 (t, *J* = 7.9 Hz, 1H), 7.19 (t, *J* = 7.5 Hz, 1H), 7.12 (t, *J* = 6.6 Hz, 1H), 7.04 (d, *J* = 8.9 Hz, 2H), 3.90 (s, 3H), 2.45 (s, 4H), 2.23 (s, 3H). ^13 ^C NMR (125 MHz, DMSO-*d*_6_) δ 165.57, 159.85, 154.19, 153.68, 148.40, 137.43, 136.32, 136.26, 132.86, 130.43, 125.67, 120.63, 120.30, 120.08, 113.71, 104.76, 60.54, 56.46, 54.77, 46.94, 46.18. HRMS: calcd for C_28_H_31_N_4_O_4_S (M + H)^+^ 519.1988, found 519.2007. HPLC analysis: MeOH-H_2_O (85: 15), 7.35 min, 96.8% purity.

***N*-(6–(2-fluoro-3-methoxyphenyl)benzo[d]thiazol-2-yl)-4–(4-methylpiperazin-1-yl)benzamide (12d)** White solid 64.8 mg, yield 52%. Mp 232.5–235.2 °C. ^1^H NMR (500 MHz, DMSO-*d*_6_) δ 12.59 (brs, 1H), 8.17 (s, 1H), 8.07 (d, *J* = 8.8 Hz, 2H), 7.83 (d, *J* = 8.4 Hz, 1H), 7.60 (d, *J* = 8.3 Hz, 1H), 7.24 (t, *J* = 7.9 Hz, 1H), 7.19 (t, *J* = 7.5 Hz, 1H), 7.12 (t, *J* = 6.6 Hz, 1H), 7.04 (d, *J* = 8.9 Hz, 2H), 3.90 (s, 3H), 2.45 (s, 4H), 2.23 (s, 3H). ^13 ^C NMR (125 MHz, DMSO-*d*_6_) δ 165.59, 160.39, 154.19, 150.12, 148.69, 148.32, 148.23, 148.17, 132.55, 130.65, 130.44, 129.38, 129.29, 127.56, 127.54, 125.01, 124.97, 122.36, 122.28, 120.49, 120.24, 113.70, 113.24, 56.58, 54.75, 46.92, 46.15. HRMS: calcd for C_26_H_26_FN_4_O_2_S (M + H)^+^ 477.1682, found 477.1653. HPLC analysis: MeOH-H_2_O (85: 15), 7.58 min, 95.5% purity.

### *General method for preparation of compounds 18a-18c* (exemplified by 18a)

**4–(4-methylpiperazin-1-yl)-*N*-(5-phenethyl-1*H*-1,2,4-triazol-3-yl)benzamide (18a)** white solid 145.4 mg, yield 32%. Mp 218.1–222.5 °C. ^1^H NMR (500 MHz, DMSO-*d*_6_) δ 13.14 (brs, 1H), 11.59 (brs, 1H), 7.96 (d, *J* = 7.7 Hz, 2H), 7.24–7.28 (m, 4H), 7.18 (t, *J* = 7.1 Hz, 1H), 7.00 (d, *J* = 8.9 Hz, 2H), 3.31 (s, 4H), 3.00 (t, *J* = 7.7 Hz, 2H), 2.88 (s, 2H), 2.37–2.48 (m, 4H), 2.22 (s, 3H). ^13 ^C NMR (125 MHz, DMSO-*d*_6_) δ 165.27, 160.15, 153.93, 130.06, 128.75, 128.71, 126.38, 121.02, 113.77, 111.75, 54.80, 47.09, 46.19, 34.09, 30.25. HRMS: calcd for C_24_H_31_N_6_O_3_ (M + H)^+^ 391.2168, found 391.2083. HPLC analysis: MeOH-H_2_O (85: 15), 9.05 min, 97.5% purity.

***N*-(5–(3-methoxyphenethyl)-1H-1,2,4-triazol-3-yl)-4–(4-methylpiperazin-1-yl) benzamide (18 b)** White solid 82.1 mg, yield 31%. Mp 216.4–219.5 °C. ^1^H NMR (500 MHz, DMSO-*d*_6_) δ 13.09 (brs, 1H), 11.55 (brs, 1H), 7.96 (d, *J* = 5.2 Hz, 2H), 7.19 (t, *J* = 7.8 Hz, 1H), 7.00 (d, *J* = 8.9 Hz, 2H), 6.81 (d, *J* = 7.6 Hz, 2H), 6.75 (d, *J* = 8.4 Hz, 1H), 3.72 (s, 3H), 3.31 (s, 4H), 2.97 (t, *J* = 7.5 Hz, 2H), 2.88 (d, *J* = 8.6 Hz, 2H), 2.37–2.47 (m, 4H), 2.23 (s, 3H). ^13 ^C NMR (125 MHz, DMSO-*d*_6_) δ 165.29, 159.70, 153.93, 130.05, 129.74, 120.96, 114.42, 113.77, 111.80, 55.33, 54.80, 47.11, 46.21, 34.05. HRMS: calcd for C_23_H_29_N_6_O_2_ (M + H)^+^ 421.2274, found 421.2215. HPLC analysis: MeOH-H_2_O (85: 15), 8.80 min, 97.2% purity.

***N*-(5–(3,5-dimethoxyphenethyl)-1H-1,2,4-triazol-3-yl)-4–(4-methylpiperazin-1-yl)benzamide (18c)**. White solid 77.8 mg, yield 31%. Mp 220.9–223.3 °C. ^1^H NMR (500 MHz, DMSO-*d*_6_) δ 13.11 (brs, 1H), 11.60 (brs, 1H), 7.96 (s, 2H), 6.94–7.05 (m, 2H), 6.38–6.41 (m, 2H), 6.31 (s, 1H), 3.71 (s, 6H), 3.31 (s, 4H), 2.79–2.97 (m, 4H), 2.40–2.47 (m, 4H), 2.22 (s, 3H). HRMS: calcd for C_24_H_31_N_6_O_3_ (M + H)^+^ 451.2379, found 451.2351. HPLC analysis: MeOH-H_2_O (85: 15), 8.72 min, 96.9% purity.

### Biological evaluation

#### Kinase profiling

The kinase profiling of **9 u** was conducted using Elisa kinase assay and the Eurofins Kinase Profiler Selectivity Testing Service.

*Elisa kinase assay*. The effects of the indicated compounds on the activities of receptor tyrosine kinases were determined using enzyme-linked immunosorbent assays (ELISAs) with purified recombinant proteins. Briefly, 20 μg/mL poly (Glu,Tyr)_4:1_ (Sigma, St Louis, MO, USA) was pre-coated in 96-well plates as a substrate. A 50 μL aliquot of 10 μmol/L ATP solution diluted in kinase reaction buffer (50 mmol/L HEPES [pH 7.4], 50 mmol/L MgCl_2_, 0.5 mmol/L MnCl_2_, 0.2 mmol/L Na_3_VO_4_, and 1 mmol/L DTT) was added to each well; 1 μL of different compounds with indicated concentration diluted in 1% DMSO (v/v) (Sigma, St Louis, MO, USA) were then added to each reaction well. DMSO (1%, v/v) was used as the negative control. The kinase reaction was initiated by the addition of purified tyrosine kinase proteins (FGFR1, FGFR2, FGFR3, FGFR4, ALK, Bcr-Abl, EPH-A2, Flt-1, RET, c-Src, IGF1R, c-Met, EGFR, ErbB2) diluted in 49 μL of kinase reaction buffer solution. After incubation for 60 min at 37 °C, the plate was washed three times with phosphate-buffered saline (PBS) containing 0.1% Tween 20 (T-PBS). Anti-phosphotyrosine (PY99) antibody (100 μL; 1:500, diluted in 5 mg/mL BSA T-PBS) was then added. After a 30 min incubation at 37 °C, the plate was washed three times, and 100 μL horseradish peroxidase-conjugated goat anti-mouse IgG (1:2000, diluted in 5 mg/mL BSA T-PBS) was added. The plate was then incubated at 37 °C for 30 min and washed 3 times. A 100 μL aliquot of a solution containing 0.03% H_2_O_2_ and 2 mg/mL *o*-phenylenediamine in 0.1 mol/l citrate buffer (pH 5.5) was added. The reaction was terminated by the addition of 50 μL of 2 mol/l H_2_SO_4_ as the colour changed, and the plate was analysed using a multi-well spectrophotometer (SpectraMAX 190, Molecular Devices, Sunnyvale, CA, USA) at 490 nm. The inhibition rate (%) was calculated using the following equation: [1-(A490/A490 control)] × 100%. The IC_50_ values were calculated from the inhibition curves in two separate experiments.

##### Cell proliferation assay

Human gastric cancer cell line SNU-16 was purchased from American Type Culture Collection (ATCC, Manssas, VA, USA). All the cell lines were routinely maintained in complete medium according to the suppliers’ recommendations.

Cells were seeded in 96-well tissue culture plates. On the day when seeding, the cells were exposed to various concentrations of compounds and further cultured for 72 h at 37 °C. Finally, cell proliferation was determined using Cell Counting Kit (CCK-8) or the thiazolyl blue tetrazolium bromide (MTT, from Sigma-Aldrich, St. Louis, MO, USA) assay. The IC_50_ values were calculated by concentration–response curve fitting using a SoftMax pro-based four-parameter method.

## Results and discussion

### Rational drug design

Most selective FGFR1 inhibitors are ATP-competitive inhibitors that bind to the ATP-binding pocket of FGFR. The ATP binding pocket of the FGFR1 consists of five regions^3^^,23,24^: (i) the adenine region, (ii) hydrophobic region I, (iii) hydrophobic region II, (iv) the nucleotide domain, (v) and the phosphate region. The adenine region is considered as the major binding site in which heterocycle scaffolds are anchored through several H-bonds with a hinge region of the kinase (Figure 1, red colour). Meanwhile, the hydrophobic region I (Figure 1, blue colour) and hydrophobic region II (Figure 1, pink colour) are the other two important sites, which plays key factor in binding small molecules by interacting with lipophilic moieties hydrophobically, and forms van der Waals interactions and H-bonds.

Accordingly, the inhibitors are commonly involving the following pharmacophore features. (i) The core structure of most inhibitors consists of a flat hetero aromatic ring system that contains at least one H-bond acceptor and H-bond donor, then it can form hydrogen bonds with the key amino acid residues: Glu562 and Ala564. (ii) Terminal hydrophobic head often interacts with the hydrophobic region I, which is a phenyl ring with different extra hydrophobic substitutions. (iii) Another hydrophobic scaffold directly linked to the flat hetero aromatic ring system which occupies the hydrophobic region II. Herein, we initially pay attention to explore the novel hetero aromatic ring, which was the core structure of FGFR1 inhibitor, formed the key H-bond interaction with hinge region, and connected the hydrophobic region I and II.

Furthermore, we superimposed the two FGFR1 crystal structures of **4V05** and **3TTO**, which were complexed with **AZD4547**^25^ and **NVP-BGJ 398**^17^, respectively. The results revealed that **AZD4547** and **NVP-BGJ 398** were similar in their binding mode with the target were similarly, when there was a significant spatial distance constraint between the pharmacophore features. The spatial distance of the hydrophobic head and the heterocycle ring is between 5.9 Å and 6.5 Å (Figure 2(a) and Figure S2). Therefore, the FGFR1 inhibitors might contain the corresponding pharmacophore features, and these pharmacophore features were also consisted to a space distance constraint.

The fragment library was derived from kinase hinge region directed library, which contained 11809 scaffolds. Subsequently, with the built pharmacophore model and molecular docking, seven hit fragments were obtained through serval round filtration (Supplementary Table S1 and Figure S4). One of them was indazole scaffold, which was proved to be an efficiency FGFR1 inhibitor scaffold^22^, which indicated the accuracy of virtual screening protocol. Then, in view of the difficulties in synthesis, only 1*H*-1,2,4-triazole, benzothiazole and indazole scaffold were selected for further modification (Figure 2(b)). The novel 1*H*-1,2,4-triazole and benzothiazole scaffolds were selected for the study of FGFR1 inhibitors research. Furthermore, we also continued to optimise the original indazole derivatives, improve the physicochemical property and enrich the structure-activity relationship of indazole derivatives. Finally, we synthesised several indazole derivatives through introduced halogen substituents at various positions. As a result, with the fragment-based virtual screening strategy, novel derivatives bearing 1*H*-1,2,4-triazole, benzothiazole and indazole scaffold were designed, synthesised and evaluated.

### Chemistry

**Scheme 1.**
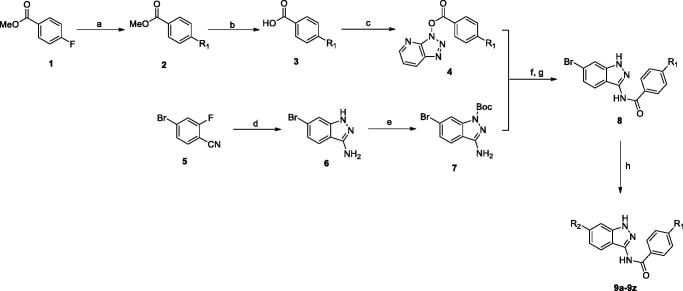
Synthesis of indazole derivatives. Reagents and conditions: (a) K_2_CO_3_, DMSO, 120 °C, 85–90%; (b) NaOH, MeOH, H_2_O, 80 °C, 93–96%; (c) HATU, K_2_CO_3_, DMF, rt, 62–70%; (d) hydrazine hydrate, *n*-butanol, 120 °C, 86%; (e) Boc_2_O, DMAP, THF, 89%; (f) NaH, THF, 60 °C, 52–61%; (g) TFA, CH_2_Cl_2_, 0 °C, 70%; (h) R-B(OH)_2_, Cs_2_CO_3_, Pd(dppf)Cl_2_, dioxane, 120 °C, 30–48%.

**Scheme 2.**
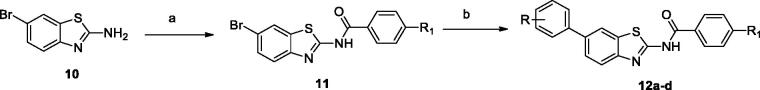
Synthesis of benzothiazole derivatives. Reagents and conditions: (a) **4**, NaH, THF, 60 °C, 51–62%; (b) R-B(OH)_2_, Cs_2_CO_3_, Pd(dppf)Cl_2_, dioxane, 120 °C, 47–53%.

**Scheme 3.**
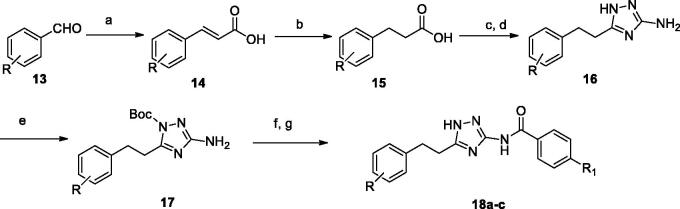
Synthesis of 1*H*-1,2,4-triazole derivatives. Reagents and conditions: (a) propanedioic acid, pyridine, piperidine, 105 °C, 77–82%; (b) Pd/C, H_2_, EtOH, 88–93%; (c) SO_2_Cl_2_, reflux; (d) amino guanidine hydrochloride, 140 °C, 49–53%; (f) **4**, NaH, THF, 60 °C, 54–62%; (g) TFA, CH_2_Cl_2_, 0 °C, 70%.

The preparation of target compounds **9a-t, 12a-d** and **18a-c** was described in Schemes 1–3. Compounds **9a-t** were synthesised from starting material 4-fluorobenzoate (**1**) and 4-bromo-2-fluorobenzonitrile (**2**) through seven steps. The nucleophilic substitution reactions of 4-fluorobenzoate with various piperazines afforded the methyl ester **2** intermediate, which then could hydrolysis to carboxylic acid **3**. Subsequently, the carboxylic acid **3** was condensed with HATU to provide the activated ester reagent **4**. Meanwhile, the intermediate **5** could be condensed by 4-bromo-2-fluorobenzonitrile **1** with hydrazine. Then nitrogen atom at the 1-position of 1*H*-indazol-3-amine scaffold **5** was protected by BOC group. The key intermediate **8** was obtained by condensation of **4** and **7** in the presence of NaH. Treatment of the intermediate **8** with various substituted-phenylboronic acid under Suzuki coupling condition^26^ of Pd(dppf)Cl_2_ and Cs_2_CO_3_ in refluxing dioxane/water gave target compounds. According to the similar synthetic route, compounds **12a-d** were synthesised from the starting material 6-bromobenzo[*d*]thiazol-2-amine (**10**) through two steps.

The synthesis of the other 1*H*-1,2,4-triazole derivatives was shown in Scheme 3. The starting material substituted-benzaldehyde (**13**) was condensed with propanedioic acid in the presence of pyridine and piperidine at 105 °C *via* Aldol condensation, which the intermediate **14** was obtained. After hydrogenation of **14** released the corresponding aliphatic acid **15**, which was connected with the amino guanidine hydrochloride giving triazole scaffold **16** in 49.1% yield. Then nitrogen atom at the 1-position of the 1*H*-1,2,4-triazole heterocycle **16** was protected by BOC group. The target compounds **18a-c** were prepared in similar way by following condensation and Suzuki-coupling procedures.

### Biological evaluation

#### Biological evaluation for various scaffold derivatives 9a-d, 12a-d, 18a-c

Initially, we designed and synthesised the indazole (**9a**-**d**), benzothiazole (**12a**-**d**) and 1*H*-1,2,4-triazole (**18a**-**c**) derivatives, with the goal of analysing the effects of different scaffolds on biological activity (Table 1). All these compounds were evaluated against FGFR1 enzyme and SNU-16 cell line. The results indicated that when the heterocycle was substituted by a 1*H*-1,2,4-triazole scaffold, it would result in almost no enzyme inhibitory activities and cell anti-proliferative activities. Furthermore, the corresponding benzothiazole derivatives **12a**-**d** were also revealed poor inhibitory activity against FGFR1 enzyme (IC_50_ >1000 nM), thus led to slight reduction cellular anti-proliferative activities.

**Table 1.**
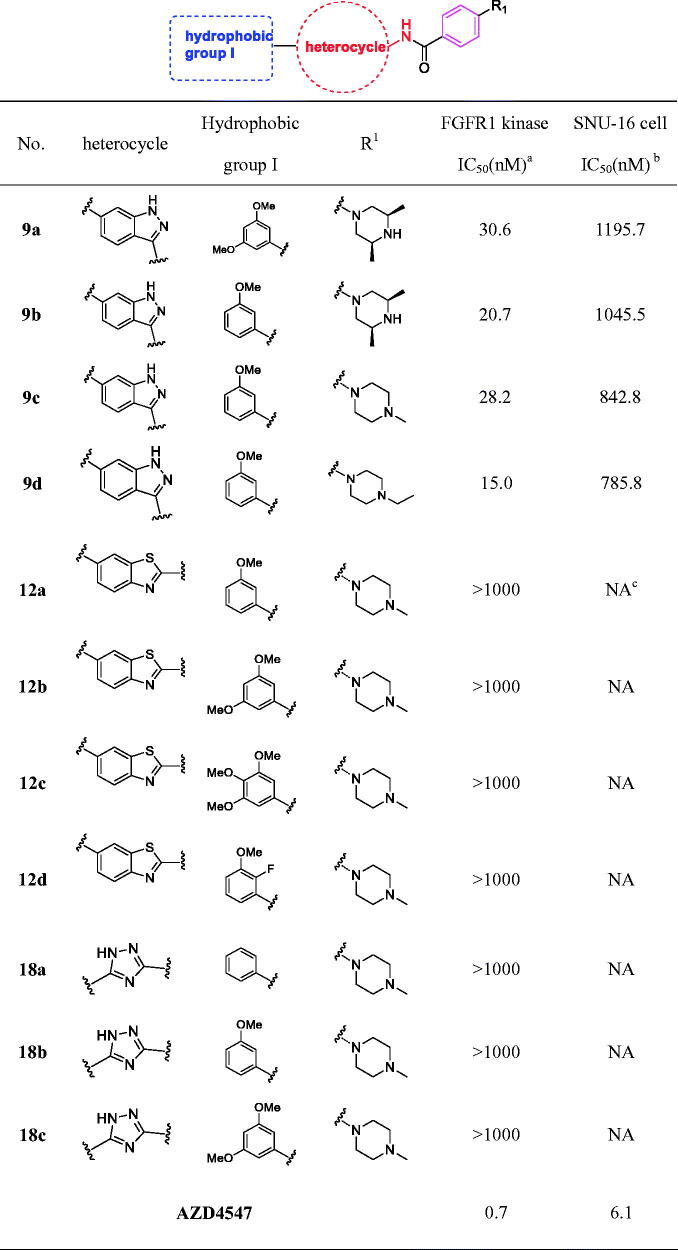
Structures and activities of various derivatives 9a-d, 12a-d, and 18a-c.

^a^ The IC_50_ values are shown as the average values from two separate experiments.

^b^ SNU-16 is FGFR2-amplified cell type.

^c^ “NA” means IC_50_ > 5 μM.

Fortunately, compound **9a** with an indazole scaffold exhibited good FGFR1 enzyme inhibitory activity (IC_50_ = 30.6 nM) and modest cellular inhibition (IC_50_ = 1195.7 nM). While the 3,5-dimethoxy phenyl substituent was replaced with the 3-methoxy phenyl group, the enzyme inhibition of **9 b** was relatively lower than **9a**. However it was more potent in cellular assay (**9a** *vs* **9 b**). Besides, co-crystal structure analysis revealed that the R_1_ substituent is at the solvent region of the target protein, which might be helpful to improve membrane permeability and enhance cellular potencies. Then the other two derivatives **9c** and **9d** with *N*-methyl piperazine and *N*-ethyl piperazine substituent at the R_1_ position, respectively, were synthesised and evaluated. As a result, the **9d** demonstrated the best enzyme inhibitory (IC_50_ = 15.0 nM) and cellular anti-proliferative activities (IC_50_ = 785.8 nM). The above results demonstrated that the heterocycle could significantly affect the enzyme and cell growth inhibitory activity.

#### The structure-activity relationship of indazole derivatives 9e-z

The hydrophobic group I located in the hydrophobic cavity of the active site and formed strong hydrophobic interaction with the target. To increase the enzyme inhibitory activity, we initially optimised the hydrophobic group I (Table 2). It was indicated that 3-methoxy phenyl (**9d**, IC_50_ = 15.0 nM) was substituted by the 3-ethoxy phenyl (**9e**, IC_50_ = 13.2 nM), 3-*iso*propoxy phenyl (**9f**, IC_50_ = 9.8 nM) caused increase of activity. On the contrary, the compound **9 h** with 3-s-butoxy phenyl exhibited low enzymatic inhibitory activity (IC_50_ > 1000 nM). Meanwhile, the compound with 3-COOMe-phenyl (**9 h**, IC_50_ = 77.7 nM), 3,5-dichloro-phenyl (**9k**, IC_50_ = 67.2 nM), 2,5-dichloro-phenyl (**9 l**, IC_50_ = 50.6 nM), 2,3-dichloro-phenyl (**9 m**, IC_50_ = 44.8 nM), 2,6-methyl-phenyl (**9q**, IC_50_ = 41.7 nM), shown moderate FGFR1 inhibitory activity. On the other hand, compounds containing a substituent either the electron-withdrawing or electron-donating group at *para*-position, were significant decreasing their activities (**9d** *vs* **9i**; **9k**, **9 l** and **9 m** *vs* **9n**; **9 m** *vs* **9p**). Furthermore, the additional halogen atoms on phenyl might be helpful to improve physicochemical properties and enhance the cell anti-proliferative activities. Thus, the compound **9o** (IC_50_ = 5.5 nM) containing a fluorine atom at the *para*-position of phenyl showed better inhibitory activity than their counterparts lack of these atoms (**9d**, IC_50_ = 15.0 nM).

**Table 2.**
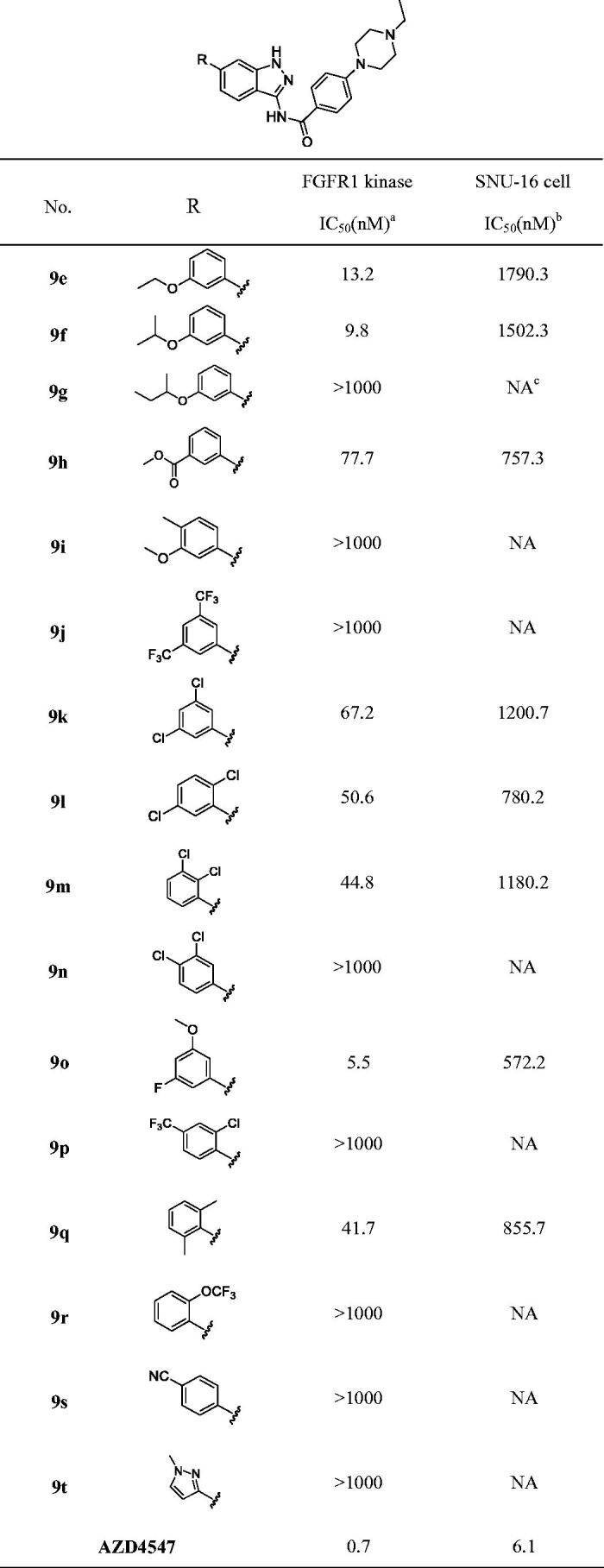
Structures and activities of indazole derivatives 9e–t.

^a^ The IC_50_ values are shown as the average values from two separate experiments.

^b^ SNU-16 is FGFR2-amplified cell type.

^c^ “NA” means IC_50_ > 5 μM.

The active compounds **9e**, **9f**, **9 h**, **9k**, **9 l**, **9 m**, **9o** and **9q** were also selected for further evaluation of their cellular potencies (SNU-16 cell). Although, the FGFR1 enzyme inhibitory activities of compound **9 h** and **9j** were higher than that of **9d**, while the cell anti-proliferative activities were similar. Besides, compound **9o** bearing a fluorine atom at *meta*-position of phenyl resulted in 1.4-fold enhancement of the cellular inhibition (**9o** vs **9d)**, which exhibited the best enzyme and cellular inhibitory activity of these indazole compounds **9e-t**.

As the above reported, R_1_ group located at the solvent region of the target protein, which might be help to improve membrane permeability and enhance cell anti-proliferative activities. Another six compounds **9 u**-**z** were designed, prepared and evaluated for their biological activities (Table 3). The results of inhibition of FGFR1 assay revealed that (*2S*, *6 R*)-2,6-dimethyl-1-phenylpiperazine (**9v**, IC_50_ = 13.2 nM), and *N*-methyl-4-phenylpiperazine (**9 y**, IC_50_ = 10.2 nM) led to slight reduction potency comparing to *N*-ethyl-4-phenylpiperazine (**9o**, IC_50_ = 5.5 nM), while introducing of a 1-benzyl-4-methylpiperazine group (**9x**, IC_50_ > 1000 nM) demonstrated poor inhibitory activity against FGFR1 enzyme. Meanwhile, the FGFR1 inhibitory activities of compounds **9 u**, **9w** and **9z** were enhanced, and the lead compound **9 u** was 1.5-fold enhancement. More importantly, the cellular potencies were improved by introducing various R_1_ group (**9 u**, **9v** and **9w** vs **9o**), and among them **9 u** exhibited the good FGFR1 inhibitory activity and cell anti-proliferative activity. Therefore, we selected **9 u** as the lead compound for further pharmacological research.

**Table 3.**
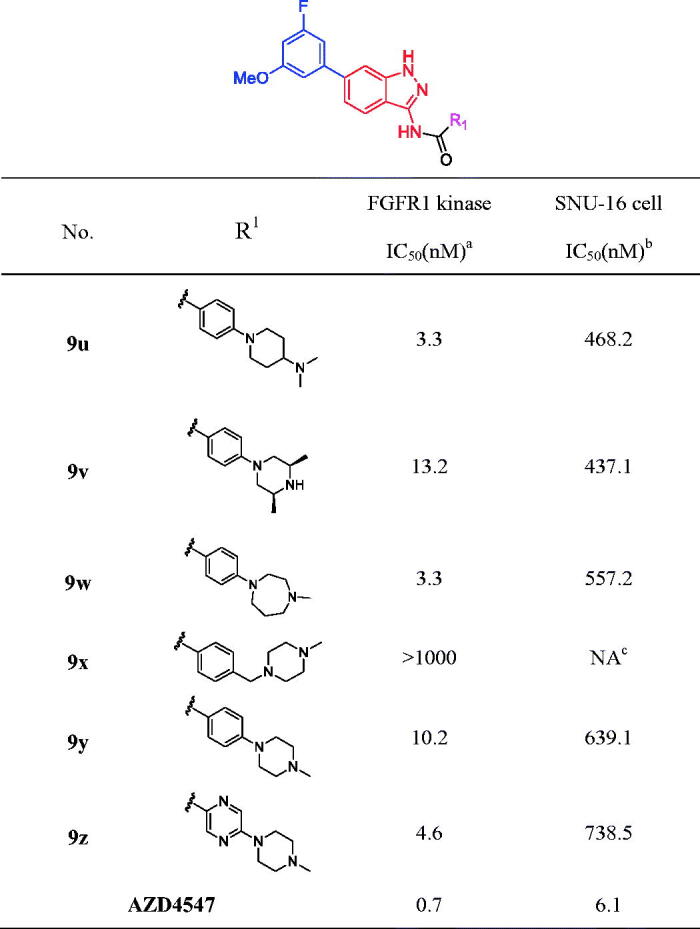
Structures and activities of indazole derivatives 9 u–z.

^a^ The IC_50_ values are shown as the average values from two separate experiments.

^b^ SNU-16 is FGFR2-amplified cell type.

^c^ “NA” means IC_50_ > 5 μM.

#### The kinase selectivity profile of compound 9 u

To identify the kinase selectivity of the lead compound **9 u**, the pharmacological experiment was first evaluated by testing it against our in-house panel of tyrosine kinases. The results were summarised in Table 4, **9 u** effectively inhibited the potent activities of FGFR1-3 with IC_50_ values of 3.3 nM, 5.2 nM and 12.2 nM, respectively. However, **9 u** exhibited low enzymatic inhibitory activity (IC_50_ > 1000 nM), which indicated that **9 u** is a selective FGFR1-3 inhibitor. The reason was that the sequence of FGFR1-3 was very similar, while the sequence of FGFR4 was quite different. Therefore, the binding mode of FGFR1-3 inhibitor was different from FGFR4 inhibitor, and the pharmacophore of FGFR4 inhibitor usually contained an acrylamide group ^[^^27–29^^]^. Furthermore, IC_50_ values for most of kinases were greater than 1 μM except for Flt-1, RET and c-Src, which were inhibited at sub-micromolar concentrations of the compound. As a result, compound **9 u** exhibited good kinase selectivity.

**Table 4.** Selectivity of 9 u in a panel of kinases.

Tyrosine kinase	IC_50_ (nM)	Tyrosine kinase	IC_50_ (nM)
FGFR1	3.3	Flt-1	>100
FGFR2	5.2	RET	>100
FGFR3	12.2	c-Src	>100
FGFR4	>1000	IGF1R	>1000
ALK	>1000	c-Met	>1000
Bcr-Abl	>1000	EGFR	>1000
EPH-A2	>1000	ErbB2	>1000

### Molecular docking studies

The docking simulation was performed to elucidate the binding mode of the various scaffold derivatives **9d**, **12a** and **18 b**, the potent indazole inhibitor **9e** and **9f**, the inactive inhibitor **9 g** with FGFR1. These compounds were docked into the ATP-binding pocket of FGFR1 (4ZSA).

**Figure 3.**
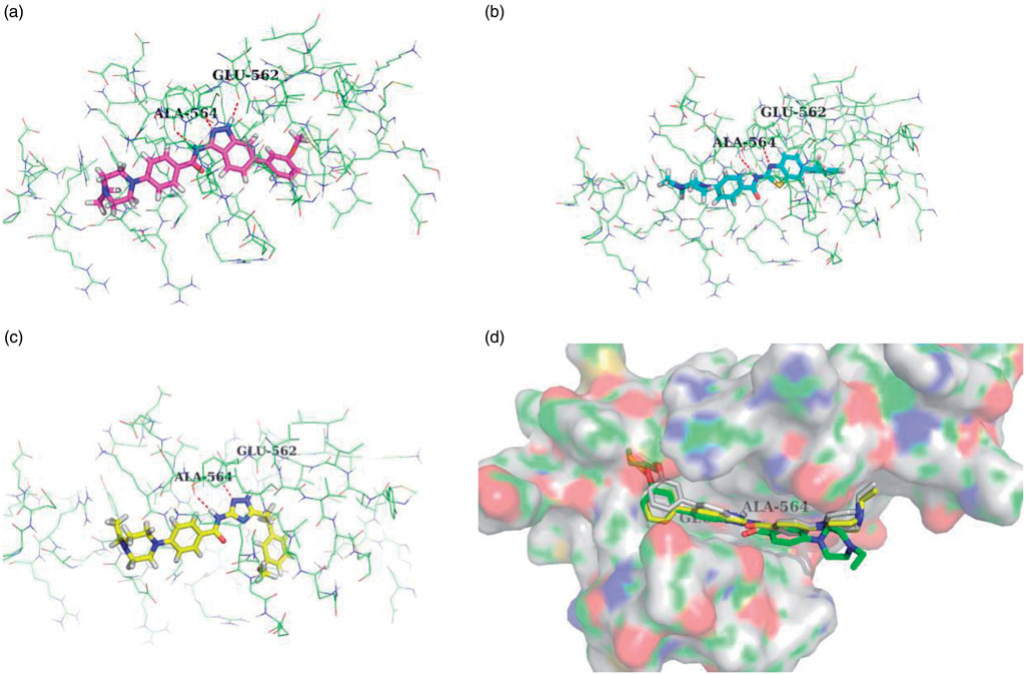
The docking mode of compound 9d (a), 12a (b), and 18 b (c). (d) Superposed docking poses of 9e (white), 9f (yellow), and 9 g (green).

As a result, the core indazole ring of compound **9d** was entered the adenine region by forming the essential H-bond interaction with hinge residue Glu562 and Ala564 (docking score: 8.523), while the N-H of indazole scaffold formed H-bond with Glu562, and the nitrogen atom of indazole scaffold and N-H of amide formed another two hydrogen bond with Ala564 (Figure 3(a)). Meanwhile, the 3-methoxy phenyl group occupied the hydrophobic area I, and hydrophobic interact with the lipophilic residue Leu547, Val561 and Phe 642. However, while the hetero aromatic rings were converted to 1*H*-1,2,4-triazole or benzothiazole, there was weakly H-bond interaction with Ala564, and no H-bond interaction with Glu562 (Figure 3(b,c)). Besides, the hydrophobic group I was deviated from hydrophobic pocket. The above docking results might explain why the triazole and benzothiazole derivatives lost their biological activities (**12a** docking score: 6.380, **18 b** docking score: 6.588).

Additionally, the methoxy group of **9d** was inserted into a small hydrophobic cavity of the hydrophobic area I. While this cavity could contain a certain size of lipophilic group, thus the ethyloxy substituent of **9e** (docking score: 8.983) and the *iso*-propyxoy substituent of **9f** (docking score: 9.451) were more appropriate to this hydrophobic cavity, meanwhile **9e** and **9f** shown more potent FGFR1 inhibitory activity than **9d.** However, the cavity could not accommodate large volume groups, including the *sec*-butoxy group (**9 g** docking score: 5.668, IC_50_ > 1000 nM). The molecular simulation results may further confirm the inhibitory potency of **9e** and **9f** against FGFR1, and the **9 g** was inactive (Figure 3(d)).

## Conclusion

In summary, we designed three novel series of FGFR1 inhibitors bearing indazole, benzothiazole, and 1*H*-1,2,4-triazole scaffold *via* fragment-based virtual screening. Interestingly, 33 new compounds were synthesised and evaluated for their inhibitory activity against FGFR1. Initially, the indazole derivative **9d** was identified as a promising FGFR1 inhibitor, with the good enzymatic inhibition (IC_50_ = 15.0 nM) and modest anti-proliferative activity (IC_50_ = 785.8 nM). Then, the hit **9d** was further optimised, through two rounds of optimisation, the compound **9 u** stood out as the most potent FGFR1 inhibitors with the best enzyme inhibitory (IC_50_ = 3.3 nM) and cellular activity (IC_50_ = 468.2 nM). Moreover, **9 u** also exhibited good kinase selectivity. Meanwhile, the docking study was performed to investigate the putative interaction mechanism with the FGFR1 target. Further studies on the structural optimisation and biological evaluation of **9 u** are currently underway in our laboratory. Our study would provide a basis for discovering novel FGFR1 inhibitors.

## Supplementary Material

Supplemental Material
