# Supplementary material for: Design, synthesis and biological evaluation of novel 1H-1,2,4-triazole, benzothiazole and indazole-based derivatives as potent FGFR1 inhibitors viafragment-based virtual screening
Source: J Enzyme Inhib Med Chem. 2019 Nov 4;35(1):72–84. doi: 10.1080/14756366.2019.1673745 (PMC6844396; doi:10.1080/14756366.2019.1673745)

Supporting Information of
Design, Synthesis and Biological Evaluation of Novel 1*H*-1,2,4-Triazole, Benzothiazole and Indazole-Based Derivatives as potent FGFR1 Inhibitors *via* Fragment-Based Virtual Screening

Jian Liu [a, #, *], Yu Wen [a, #], Lina Gao [a,#], Liang Gao [a], Fengjun He [a], Jingxian Zhou [a], Junwei Wang [a], Rupeng Dai [a], Xiaojing Chen [a], Di Kang [a,*] , Lihong Hu [a,*]

[a] *Jiangsu Key Laboratory for Functional Substance of Chinese Medicine, Jiangsu Collaborative Innovation Center of Chinese Medicinal Resources Industrialization, Stake Key Laboratory Cultivation Base for TCM Quality and Efficacy, School of Pharmacy, Nanjing University of Chinese Medicine, Nanjing, 210023, PR China*

*Corresponding author: liujian623@njucm.edu.cn (Jian Liu); kangdi@njucm.edu.cn (Di Kang); lhhu@njucm.edu.cn (Lihong Hu)
# Jian Liu, Yu Wen and Lina Gao contributed equally.

Pharmacophore modeling

The selection of a suitable training set was the most important step to pharmacophore model, as it determined the quality of generated pharmacophore. A series of known FGFR1 inhibitors were obtained from the literatures [1-7]. Most of these inhibitors were launched or in clinical trial.

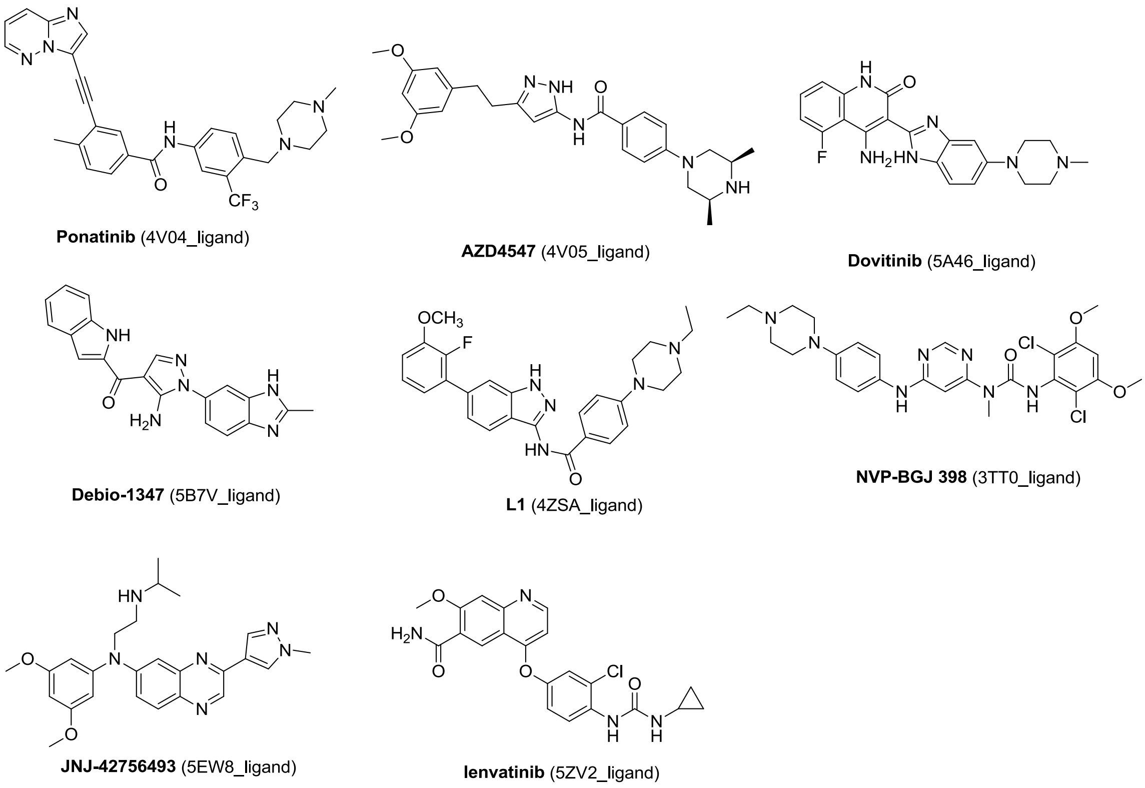

Figure S1. The dataset to build Pharmacophore

The three-dimensional pharmacophore Generation protocol in Discovery Studio 4.0 (DS) was used to build the pharmacophore models. The conformational set was generated for each molecule using the 'best-quality conformational analysis' method, based on the CHARMM force field. On the basis of the chemical features of compounds in the training set and the proposed mechanism of action, four pharmacophore features, hydrogen bond acceptor (HBA), hydrogen bond donor (HBD), hydrophobic (HY), and ring aromatic group (RA) were defined for the creation of pharmacophore models. All other parameters used were set at the default values. Pharmacophores were then computed and the top 10 scoring hypotheses were obtained. To check the pharmacophore's capability of distinguishing active inhibitors from inactive compounds and predicting their activity values, the developed pharmacophore models were validated by three methods: Cost analysis, Test set prediction and Fischer's randomization test. A best pharmacophore hypothesis should meet the following

requirements: the total cost close to the fixed cost value and away from the null cost value. Meanwhile, the testing set was also prepared using the same protocol and utilized to verify the predictability of the best pharmacophore hypothesis. Fischer's randomization test was performed to obtain cross validation via randomizing the data.

(a).

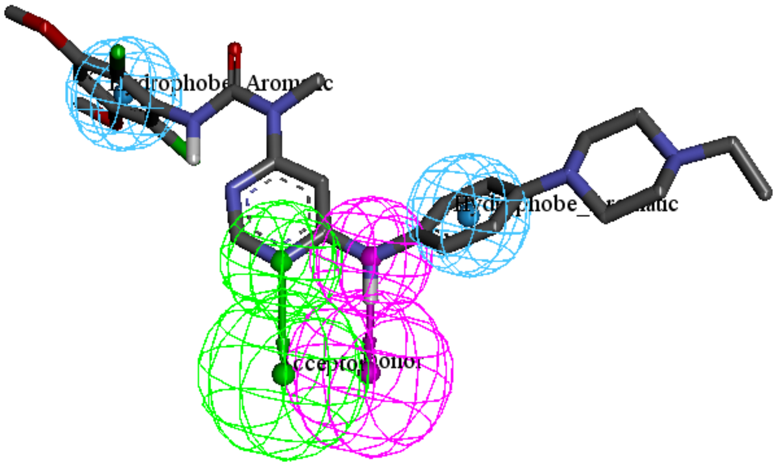

(b).

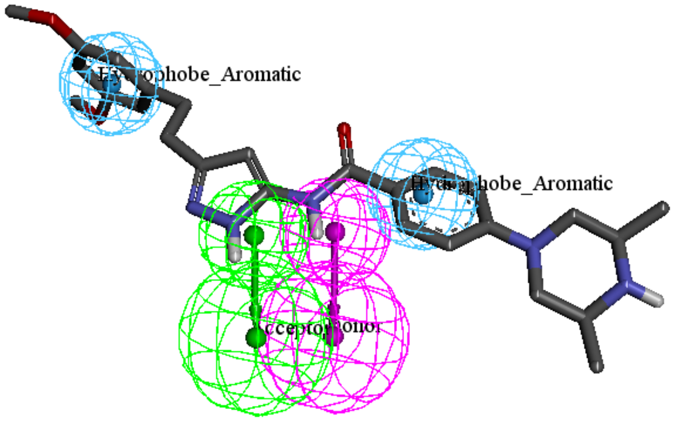

Figure S2. Compound **AZD4547** (**Fig S2a**) and **NVP-BGJ398** (**Fig S2b**) mapped the pharmacophore model. Pharmacophore features colored as follow: HBA (green), HBD (magenta), hydrophobic (cyan).

Fragment-Based Virtual Screening

In the Protein Data Bank (PDB), we selected 8 crystal structures of the FGFR (PDB ID: 4V04、4V05、5A46、5B7V、4ZSA、3TT0、5EW8、5ZV2) for further docking study. In view of available FGFR structures with a resolution<2 Å and good ligand-receptor interactions in binding cavity which can be observed by PyMOL. Afterwards, we used the Glide program in Maestro 9.4 to dock proteins with inhibitors.

The docking reliability was evaluated by calculating the root-mean-square deviation (heavy atoms) difference between the reference position of the ligand in the crystal structures and that predicted by the docking software. Finally, 4ZSA was selected as the template protein for follow-up virtual screening which had low re-docking value of RMSD.

The fragment library was derived from kinase hinge region directed library provided by enamine (https://enamine.net/index.php), which contained 11809 fragments. As the pharmacophore were constructed, we initially utilized this model for the first round of virtual screening. The well-validated pharmacophore model was used as a 3D structural query for retrieving potent compounds and estimating their inhibitory activity of FGFR1. The database screening was performed using the Ligand Pharmacophore Mapping protocol in DS 4.0. The fit values were calculated based on the chemical substructures map the location constraints of the pharmacophoric features and their distance deviation from the feature centers (**Table S1**). Finally, only the compounds both showed good fit values and the chemical features mapped the location distance constraints in pharmacophore model, could enter the next stage of virtual screening calculation (**Figure S3**).

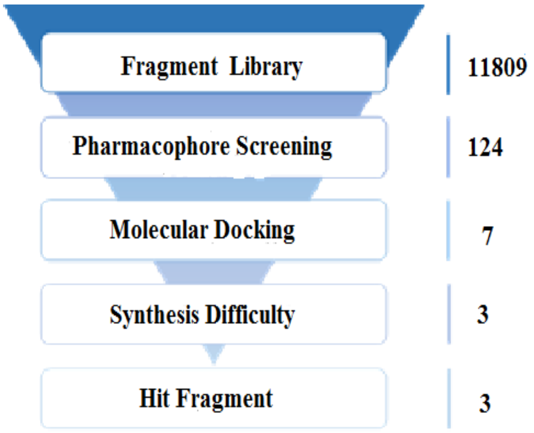

Figure S3. The fragment-based virtual screening protocol.

The Surflex-dock module in Sybyl-x 2.0 was used for molecular docking. For checking the robustness of docking protocol, self-docking was performed in which the bound ligand was re-docked into the catalytic site of protein. In both cases, the ligand-binding site search region was defined to center on the ligand in the crystal structure. The crystal structure of the FGFR1 (PDB ID: 4ZSA) was considered for this study and prepared using Protein Preparation Wizard module. Hydrogen atoms were added, water molecules in all the system were removed, followed by energy minimization and

optimization by MMFF94 force field. LigPrep module was employed to prepare the compounds for molecular docking. To prepare ligand structures, hydrogens were added, 3D geometries, ionization and tautomeric states were generated. Finally, the ligand structures were minimized using MMFF94 with 5000 iterations and minimum RMS gradient 0.05. Other parameters were as default. The compounds were selected based on the docking score, seven fragments were select for the next filtration (**Table S1**).. Finally, as for these seven fragments, we use SciFinder search tool to evaluate the difficulty of synthesis of target compounds, therefore only three target fragments for the next experimental research, including 1*H*-1,2,4-triazole, benzothiazole and indazole scaffold (**Figure S4**).

(a).

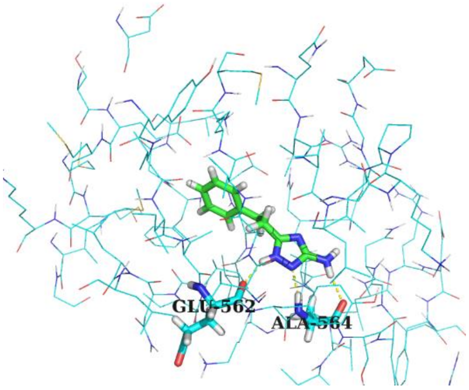

(b).

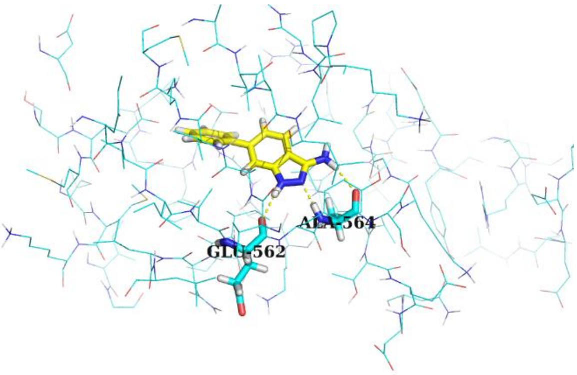

(c).

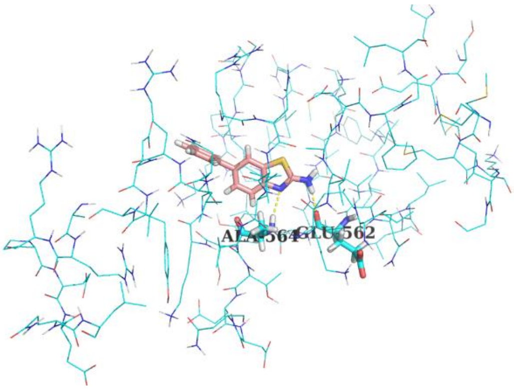

Figure S4. The docking results of 1*H*-1,2,4-triazole (**4a**), indazole (**4b**) and benzothiazole (**4c**) scaffold

Table S1. Fragment-Based Virtual Screening Results

	Fragments	Fit value	Distance	Surflex-dock
**F1**	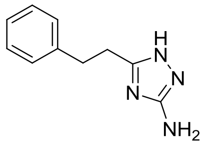	6.67	6.4 Å	6.01
**F2**	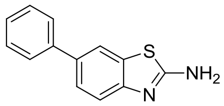	6.88	5.9 Å	5.99
**F3**	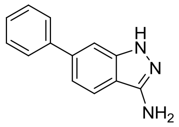	7.02	6.2 Å	5.45
**F4**	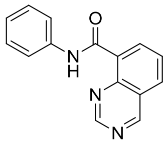	5.60	6.4 Å	4.68
**F5**	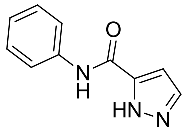	6.05	6.5 Å	5.33
**F6**	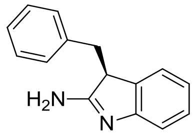	6.23	6.3 Å	5.63
**F7**	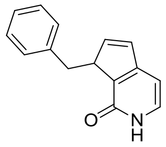	5.98	6.3 Å	5.12

Experimental Section

1.1 Chemistry

Solvents were distilled under the positive pressure of dry argon before use and dried using standard methods. Chemicals were obtained from local suppliers and were used without further purification. All reactions were monitored by thin-layer chromatography (silica gel 60 F254 glass plates). NMR spectra were recorded on Bruker 400 MHz instruments, and the chemical shifts were presented in terms of parts per million with TMS as the internal reference. Electron-spray ionization mass spectra in positive mode (ESI-MS) data were obtained with a Bruker Esquire 3000+ spectrometer. Flash column chromatography was performed on silica gel (200-300 mesh, Adamas, China).

Ethyl 4-(4-methylpiperazin-1-yl)benzoate (2) Ethyl 4-fluorobenzoate (4.6 g, 29.7 mmol) was dissolved in DMSO (20 mL), then followed by the addition of K_2CO_3 (12.3 g, 89.1 mmol) and 1-ethylpiperazine (7.6 mL, 59.4 mmol). The mixture was heated to 110 °C and stirred for 10 h before cooled to rt and diluted with water (50 mL) and EtOAc (200 mL). The organic phase was washed with brine (50 mL), dried over Na_2SO_4, filtered, and concentrated. The residue was purified by silica gel column chromatography using CH_2Cl_2-MeOH (10:1) to afford **2** (6.4 g, 89%) as a yellow solid. Mp 74.0-75.2 °C. ^{1}H NMR(400 MHz, $CDCl_3$) δ: ^{1}H NMR (400 MHz, DMSO-d_6) δ: 7.78 (d, J = 8.9 Hz, 2H), 6.97 (d, J = 9.0 Hz, 2H), 4.23 (q, J = 7.1 Hz, 2H), 3.32 - 3.26 (m, 4H), 2.45 - 2.38 (m, 4H), 2.21 (s, 3H), 1.28 (t, J = 7.1 Hz, 3H).

4-(4-methylpiperazin-1-yl)benzoic acid (3) To a solution of intermediate **2** (1.0 g, 4.0 mmol) in MeOH (50 mL) and H_2O (15 mL) was added NaOH (0.48 g, 12.0 mmol) in room temperature. The mixture was stirred at 80 °C for 1 h. After completion, the organic phase was concentrated in *vacuo* and the aqueous phase was acidified to pH = 3-4 with 1 M HCl, filtered, and concentrated to dryness to afford **3** (0.82 g, 93%) as a white solid. Mp 245.0-255.2 °C. ^{1}H NMR(400 MHz, DMSO-d_6) δ: ^{1}H NMR (400 MHz, DMSO-d_6) δ: 12.38 (s, 1H), 7.76 (d, J = 8.9 Hz, 2H), 6.95 (d, J = 9.0 Hz, 2H), 3.32 - 3.18 (m, 4H), 2.46 - 2.38 (m, 4H), 2.21 (s, 3H).

3*H*-[1,2,3]triazolo[4,5-b]pyridin-3-yl 4-(4-methylpiperazin-1-yl)benzoate (4) To a solution of intermediate **3** (0.82 g, 3.8 mmol) in DMF (30 mL) was added HATU (2.2 g, 5.7 mmol), and K_2CO_3 (1.0 g, 7.6 mmol) at 0 °C. The mixture was stirred at room temperature for 3 h. After completion, the reaction was quenched with water (60 mL)

and extracted with ethyl acetate (3 × 25 mL). The combined organic phase was washed with water (30 mL) and brine (30 mL), dried over Na_2SO_4, filtered, and concentrated in *vacuo*. The residue was purified by silica column chromatography ($CHCl_2$/MeOH= 15:1) to give compound **4** (0.83 g, 65%) as yellowish solid. Mp 150.5-152.0 °C. ^{1}H NMR (500 MHz, DMSO-d_6) δ: 8.83 (dd, *J* = 4.4, 1.3 Hz, 1H), 8.75 (dd, *J* = 8.4, 1.3 Hz, 1H), 8.06 (d, *J* = 8.9 Hz, 2H), 7.66 (dd, *J* = 8.4, 4.4 Hz, 1H), 7.15 (d, *J* = 8.9 Hz, 2H), 3.50 (t, *J* = 5.0 Hz, 4H), 2.49 (s, 4H), 2.26 (s, 3H).

6-Bromo-1*H*-indazol-3-amine (6) 4-bromo-2-fluorobenzonitrile (5.0 g, 25.1 mmol) was dissolved in 1-butanol (20 mL), then followed by the addition of NH_2NH_2(1.0 mL, 50.3 mmol). The reaction mixture was heated to reflux for 4 h. Then cooled to rt, filtered, washed with *n*-hexane and dried to give **4** as white solid (4.8 g, 86%). Mp 237.5-238.4 °C. ^{1}H NMR (400 MHz, DMSO-d_6) δ: 11.52 (brs, 1H), 7.64 (d, *J* = 8.5 Hz, 1H), 7.37-7.52 (m, 1H), 7.02 (dd, *J* = 8.5, 1.6 Hz, 1H), 5.47 (brs, 2H).

***tert*-Butyl- 3-amino-6-bromo-1*H*-indazole-1-carboxylate(7)** DMAP (100.0 mg) and Boc_2O (566.1mg, 2.6 mmol) were added to the solution of building block **6** (500.0 mg, 2.4 mmol) in THF (10 mL). The reaction mixture was stirred for 1 h and monitored by TLC. After concentrated, the residue was dissolved in EtOAc (100 mL) and washed with 1M HCl (20 mL × 2), $NaHCO_3$ (20 mL ×2) and brine (20 mL × 2), dried over Na_2SO_4, concentrated in *vacuo.* The residue was purified by chromatography on silica gel Petroleum-EtOAc (1:1) to give **5** as white solid (653.7 mg, 89%). Mp 202.5-204.0°C. ^{1}H NMR (400 MHz, DMSO-d_6) δ: 8.12 (s, 1H), 7.80 (d, *J* = 8.4 Hz, 1H), 7.45 (dd, *J* = 8.4, 1.7 Hz, 1H), 6.44 (s, 2H), 1.57 (s, 9H).

***N*-(6-Bromo-1*H*-indazol-3-yl)-4-(4-methylpiperazin-1-yl)benzamide (8)** The building block **7** (250.0 mg, 0.8 mmol) was dissolved in THF (10 mL), then followed by the addition of building block **4** (405.6 mg, 1.2 mmol) and 60% NaH (160 mg, 4.0 mmol). The reaction mixture was heated to 55 °C and stirred for 0.5 h. After completion, it was quenched with water and the organic phase was concentrated in *vacuo*. Then, the residue was dissolved in EtOAc (100 mL), and washed with brine (20 mL × 2), dried over Na_2SO_4, concentrated in *vacuo.* The residue was purified by silica gel column chromatography using CH_2Cl_2-MeOH (10:1) to afford **8** (199.0 mg, 60%) as a yellow solid. Mp 218.8-220.7 °C. ^{1}H NMR (400 MHz, DMSO-d_6) δ: 12.86 (brs, 1H), 10.56 (brs, 1H), 7.94 (d, *J* = 8.7 Hz, 2H), 7.61-7.70 (m, 1H), 7.17 (d, *J* = 7.9 Hz, 1H), 6.99 (d, *J* = 8.6 Hz, 2H), 3.29-3.40 (m, 4H), 2.51-2.56 (s, 4H), 2.36 (q, *J* = 7.1 Hz, 2H), 1.02 (t, *J* = 7.2 Hz, 3H).

General method for preparation of compounds 9a-9z (exemplified by **9d**).

4-(4-ethylpiperazin-1-yl)-*N*-(6-(3-methoxyphenyl)-*1H*-indazol-3-yl)benzamide (9d) The building block **8** (100.0 mg, 0.2 mmol) was dissolved in dioxane (2mL), then followed by the addition of (3-methoxyphenyl)boronic acid (85.2 mg, 0.5 mmol), Pd(dppf)Cl_2 (20 mg), 1M Cs_2CO_3 (500 μL). The reaction mixture was heated at 120 °C. for 1 h, then it cooled to rt. After concentrated, the residue was dissolved in EtOAc (50 mL) and washed with H_2O (10 mL ×2), and brine (10 mL ×2), dried over Na_2SO_4, concentrated in *vacuo*. The residue was purified by chromatography on silica gel DCM-MeOH (10:1) to give the **9d** (45.6 mg, 44%). Mp 226.5-230.2 °C. ^{1}H NMR (400 MHz, DMSO-d_6) δ: 12.80 (brs, 1H), 10.53 (brs, 1H), 7.99 (d, *J* = 8.9 Hz, 2H), 7.78 (d, *J* = 8.6 Hz, 1H), 7.68 (s, 1H), 7.35-7.45 (m, 2H), 7.30 (d, *J* = 7.9 Hz, 1H), 7.24-7.26 (d, *J* = 2.0 Hz, 1H), 7.03 (d, *J* = 9.0 Hz, 2H), 6.97 (dd, *J* = 8.2, 2.0 Hz, 1H), 3.85 (s, 3H), 2.48-2.55 (m, 4H), 2.39 (q, *J* = 7.1 Hz, 2H), 1.05 (t, *J* = 7.2 Hz, 3H). ^{13}C NMR (100 MHz, DMSO-d_6) δ: 165.6, 160.2, 153.7, 142.6, 141.1, 139.0, 130.5, 129.8, 123.1, 122.9, 120.0, 119.9, 117.0, 113.9, 113.6, 113.1, 108.3, 55.6, 52.6, 52.1, 47.4, 12.3. ESI-MS (*m/z*): $[M+H]^+$ = 456.7 (Calcd: 456.55). HRMS: calcd for $C_{27}H_{31}N_5O_2$ $(M+H)^+$ 456.2400, found 456.2391. HPLC analysis: MeOH-H_2O (80: 20), 6.85 min, 96.8% purity.

***N*-(6-(3,5-dimethoxyphenyl)-1*H*-indazol-3-yl)-4-((*3R*,*5S*)-3,5-dimethylpiperazin-1-yl)benzamide (9a)** White solid: 29.2 mg, yield 38%; Mp 228.1-232.2 °C. ^{1}H NMR (400 MHz, DMSO-d_6) δ: 12.81 (brs, 1H), 10.53 (brs, 1H), 7.99 (d, *J* = 8.3 Hz, 2H), 7.77 (d, *J* = 8.0 Hz, 1H), 7.68 (s, 1H), 7.37 (dd, *J* = 8.6, 1.3 Hz, 1H), 7.04 (d, *J* = 9.0 Hz, 2H), 6.85 (d, *J* = 2.2 Hz, 2H), 6.54 (t, *J* = 2.1 Hz, 1H), 3.85 (s, 2H), 3.83 (s, 6H), 2.90-3.01 (m, 2H), 2.35-2.41 (m, 2H), 1.12 (s, 3H), 1.10 (s, 3H). ^{13}C NMR (100 MHz, DMSO-d_6) δ: 165.7, 161.3, 153.2, 143.3, 142.1, 141.0, 139.0, 129.9, 122.9, 120.0, 117.1, 114.0, 108.4, 105.8, 99.9, 55.8, 53.1, 50.6, 18.7. ESI-MS (*m/z*): $[M+H]^+$ = 486.1 (Calcd: 486.24). ESI-HRMS: calcd for $C_{28}H_{33}N_5O_3$ $(M+H)^+$ 486.2505, found 486.2502. HPLC analysis: MeOH-H_2O (80: 20), 7.36 min, 96.0% purity.

4-((*3R*,*5S*)-3,5-dimethylpiperazin-1-yl)-*N*-(6-(3-methoxyphenyl)-1*H*-indazol-3-yl)benzamide (9b) White solid: 24.8 mg, yield 30%; Mp 224.8-227.4 °C.^{1}H NMR (400 MHz, DMSO-d_6) δ: 12.81 (brs, 1H), 10.52 (brs, 1H), 7.98 (d, *J* = 8.9 Hz, 2H), 7.78 (d, *J* = 8.1 Hz, 1H), 7.68 (s, 1H), 7.37-7.44 (m, 2H), 7.30 (d, *J* = 7.9 Hz, 1H), 7.26 (s, 1H), 7.03 (d, *J* = 8.2 Hz, 2H), 6.97 (dd, *J* = 8.1, 2.2 Hz, 1H), 3.85 (s, 3H), 3.79-3.81 (m, 2H), 2.85-2.93 (m, 2H), 2.28-2.34 (m, 2H), 1.09 (s, 3H), 1.07 (s, 3H). ^{13}C NMR (100 MHz,

DMSO-d_6) δ: 165.7, 160.2, 153.1, 142.5, 142.2, 141.0, 139.0, 130.6, 129.9, 123.0, 119.9, 117.0, 114.1, 113.6, 113.1, 108.3, 55.6, 52.8, 50.7, 18.4. ESI-MS (*m/z*): $[M+H]^+$ = 456.1 (Calcd: 456.23). ESI-HRMS: calcd for $C_{27}H_{31}N_5O_2$ $(M+H)^+$ 456.2400, found 456.2390. HPLC analysis: MeOH-H_2O (80: 20), 7.27 min, 95.9% purity.

***N*-(6-(3-methoxyphenyl)-1*H*-indazol-3-yl)-4-(4-methylpiperazin-1-yl)benzamide (9c)** White solid: 40.2 mg, yield 45%; Mp 224.8-227.4 °C.^{1}H NMR (400 MHz, DMSO-d_6) δ: 12.82 (brs, 1H), 10.55 (brs, 1H), 8.00 (d, *J* = 8.6 Hz, 2H), 7.80 (d, *J* = 8.8 Hz, 1H), 7.65 (s, 1H), 7.35-7.45 (m, 2H), 7.29 (d, *J* = 8.0 Hz, 1H), 7.25 (d, *J* = 2.0 Hz, 1H), 7.05 (d, *J* = 8.8 Hz, 2H), 6.95 (dd, *J* = 8.2, 1.9 Hz, 1H), 3.83 (s, 3H), 2.48-2.55 (m, 4H), 2.39 (q, *J* = 7.1 Hz, 2H), 1.05 (t, *J* = 7.2 Hz, 3H). ^{13}C NMR (100 MHz, DMSO-d_6) δ: 165.5, 160.2, 153.6, 142.6, 142.2, 141.1, 138.9, 130.5, 129.8, 123.1, 123.0, 120.0, 119.8, 117.0, 113.9, 113.6, 113.1, 108.3, 55.6, 52.6, 52.1, 47.4, 12.4. ESI-MS (*m/z*): $[M+H]^+$ = 442.2 (Calcd: 442.21). ESI-HRMS: calcd for $C_{26}H_{28}N_5O_2$ $(M+H)^+$ 442.2198, found 442.2190. HPLC analysis: MeOH-H_2O (80: 20), 7.38 min, 96.5% purity.

***N*-(6-(3-ethoxyphenyl)-1*H*-indazol-3-yl)-4-(4-ethylpiperazin-1-yl)benzamide (9e)** White solid: 22.7 mg, yield 39%; Mp 223.6-226.0 °C. ^{1}H NMR (400 MHz, DMSO-d_6) δ: 12.80 (brs, 1H), 10.54 (brs, 1H), 8.00 (d, *J* = 8.9 Hz, 2H), 7.78 (d, *J* = 8.5 Hz, 1H), 7.66 (s, 1H), 7.35-7.43 (m, 2H), 7.29 (d, *J* = 7.8 Hz, 1H), 7.24 (s, 1H), 7.03 (d, *J* = 8.9 Hz, 2H), 6.96 (dd, *J* = 8.1, 1.9 Hz, 1H), 4.13 (q, *J* = 6.9 Hz, 2H), 3.26-3.40 (m, 4H), 2.51-2.62 (m, 4H), 2.34-2.45 (m, 2H), 1.37 (t, *J* = 8.0 Hz, 3H), 1.06 (t, *J* = 7.1 Hz, 3H). ^{13}C NMR (100 MHz, DMSO-d_6) δ: 165.6, 159.5, 153.6, 142.6, 142.2, 141.1, 139.0, 130.5, 129.8, 123.1, 123.0, 119.9, 117.0, 114.0, 113.9, 113.6, 108.3, 63.6, 52.5, 52.1, 47.4, 15.2, 12.3. ESI-MS (*m/z*): $[M+H]^+$ = 470.2 (Calcd: 470.25). ESI-HRMS: calcd for $C_{28}H_{33}N_5O_2$ $(M+H)^+$ 470.2556, found 470.2550. HPLC analysis: MeOH-H_2O (80: 20), 7.42 min, 96.9% purity.

4-(4-ethylpiperazin-1-yl)-*N*-(6-(3-*iso*propoxyphenyl)-1*H*-indazol-3-yl)benzamide (9f) White solid: 21.3 mg, yield 39%; Mp 227.9-231.7 °C.^{1}H NMR (400 MHz, DMSO-d_6) δ: 12.77 (brs, 1H), 10.52 (brs, 1H), 8.00 (d, *J* = 8.7 Hz, 2H), 7.78 (d, *J* = 8.5 Hz, 1H), 7.65 (s, 1H), 7.35-7.41 (m, 2H), 7.27 (d, *J* = 7.7 Hz, 1H), 7.22 (s, 1H), 7.03 (d, *J* = 8.0 Hz, 2H), 6.92 (dd, *J* = 8.3, 1.9 Hz, 1H), 4.72-4.78 (m, 1H), 3.27-3.38 (m, 4H), 2.40-2.58 (m, 4H), 2.34 (q, *J* = 7.1 Hz, 2H), 1.32 (s, 3H), 1.31 (s, 3H), 1.06 (t, *J* = 7.1 Hz, 3H). ^{13}C NMR (100 MHz, DMSO-d_6) δ: 166.6, 158.4, 153.6, 142.7, 142.2, 141.1, 139.0, 130.6, 129.8, 123.1, 119.8, 117.0, 115.0, 114.9, 114.0, 69.7, 52.5, 52.0, 47.3, 22.4, 12.3. ESI-MS (*m/z*): $[M+H]^+$ = 484.3 (Calcd: 484.27). ESI-HRMS: calcd for

$C_{29}H_{35}N_5O_2$ $(M+H)^+$ 484.2713, found 484.2706. HPLC analysis: MeOH-H_2O (80: 20), 7.22 min, 97.1% purity.

***N*-(6-(3-(*sec*-butoxy)phenyl)-1*H*-indazol-3-yl)-4-(4-ethylpiperazin-1-yl)benzamide (9g)** White solid: 23.9 mg, yield 47%; Mp 225.9-290.0 °C.^{1}H NMR (400 MHz, DMSO-d_6) δ: 12.79 (brs, 1H), 10.54 (brs, 1H), 8.00 (d, *J* = 8.9 Hz, 2H), 7.78 (d, *J* = 8.5 Hz, 1H), 7.67 (s, 1H), 7.37-7.42 (m, 2H), 7.29 (d, *J* = 7.9 Hz, 1H), 7.24-7.26 (m, 1H), 7.03 (d, *J* = 9.0 Hz, 2H), 6.96 (dd, *J* = 8.1, 1.8 Hz, 1H), 3.86 (s, 1H), 3.84 (s, 1H), 3.26-3.38 (m, 4H), 2.51-2.60 (m, 4H), 2.40-2.46 (m, 2H), 2.03-2.09 (m, 1H), 1.06 (t, *J* = 7.1 Hz, 3H), 1.03 (s, 3H), 1.01 (s, 3H). ^{13}C NMR (100 MHz, DMSO-d_6) δ: 166.6, 159.8, 153.6, 142.6, 142.2, 141.1, 139.0, 130.5, 129.8, 123.1, 119.9, 117.0, 114.0, 113.6, 108.3, 74.3, 52.5, 52.0, 47.3, 31.2, 28.3, 19.6, 12.3. ESI-MS (*m/z*): $[M+H]^+$ = 498.3 (Calcd: 498.29). ESI-HRMS: calcd for $C_{30}H_{37}N_5O_2$ $(M+H)^+$ 498.2869, found 498.2875. HPLC analysis: MeOH-H_2O (80: 20), 7.30 min, 97.0% purity.

methyl 3-(3-(4-(4-ethylpiperazin-1-yl)benzamido)-1*H*-indazol-6-yl)benzoate (9h) White solid: 27.5 mg, yield 45%; Mp 228.7-233.4 °C.^{1}H NMR (400 MHz, DMSO-d_6) δ 12.91 (brs, 1H), 10.61 (brs, 1H), 8.27 (s, 1H), 7.98-8.06 (m, 4H), 7.84 (d, *J* = 8.5 Hz, 1H), 7.74 (s, 1H), 7.65-7.69 (m, 1H), 7.42 (d, *J* = 8.6 Hz, 1H), 7.08 (d, *J* = 8.5 Hz, 2H), 3.91 (s, 3H), 3.42-3.48 (m, 4H), 2.80-2.90 (m, 4H), 2.48-2.52 (q, *J* = 7.6 Hz, 2H), 1.19 (t, *J* = 7.1 Hz, 3H). ^{13}C NMR (100 MHz, DMSO-d_6) δ: 166.7, 165.6, 153.0, 142.2, 141.5, 141.1, 137.9, 132.5, 130.9, 130.1, 129.9, 128.6, 128.0, 123.4, 119.6, 117.2, 114.3, 108.5, 52.8, 51.6, 46.1, 29.5, 10.8. ESI-MS (*m/z*): $[M+H]^+$ = 484.3 (Calcd: 484.24). ESI-HRMS: calcd for $C_{28}H_{31}N_5O_3$ $(M+H)^+$ 484.2349, found 484.2358. HPLC analysis: MeOH-H_2O (80: 20), 7.05 min, 96.5% purity.

4-(4-ethylpiperazin-1-yl)-*N*-(6-(3-methoxy-4-methylphenyl)-1*H*-indazol-3-yl) benzamide (9i) White solid: 27.7 mg, yield 41%; Mp 227.3-231.2 °C. ^{1}H NMR (400 MHz, DMSO-d_6) δ 12.83 (brs, 1H), 10.54 (brs, 1H), 8.01 (d, *J* = 8.7 Hz, 2H), 7.75 (d, *J* = 8.5 Hz, 1H), 7.60 (s, 1H), 7.54 (s, 2H), 7.33 (dd, *J* = 8.6, 1.1 Hz, 1H), 7.05 (d, *J* = 9.1 Hz, 3H), 3.84 (s, 3H), 3.33-3.38 (m, 4H), 2.59-2.64 (m, 4H), 2.46 (q, *J* = 7.3 Hz, 2H), 2.25 (s, 3H), 1.09 (t, *J* = 7.7 Hz, 3H). ^{13}C NMR (100 MHz, DMSO-d_6) δ: 165.5, 157.6, 142.4, 141.0, 138.9, 132.9, 129.9, 126.5, 126.1, 122.9, 119.5, 116.4, 114.1, 111.2, 107.3, 56.5, 55.9, 51.8, 46.6, 19.0, 16.6. ESI-MS (*m/z*): $[M+H]^+$ = 470.3 (Calcd: 470.26). ESI-HRMS: calcd for $C_{28}H_{33}N_5O_2$ $(M+H)^+$ 470.2556, found 470.2551. HPLC analysis: MeOH-H_2O (80: 20), 7.14 min, 96.3% purity.

***N*-(6-(3,5-bis(trifluoromethyl)phenyl)-1*H*-indazol-3-yl)-4-(4-ethylpiperazin-1-**

yl)benzamide (9j) White solid: 33.6 mg, yield 44%; Mp 227.5-213.3 °C. ^{1}H NMR (400 MHz, DMSO-d_6) δ: 12.95 (brs, 1H), 10.59 (brs, 1H), 8.42 (s, 2H), 8.13 (s, 1H), 8.00 (d, *J* = 8.9 Hz, 2H), 7.94 (s, 1H), 7.87 (d, *J* = 8.6 Hz, 1H), 7.54 (d, *J* = 8.2 Hz, 1H), 7.04 (d, *J* = 8.0 Hz, 2H), 3.27-3.38 (m, 4H), 2.44-2.52 (m, 4H), 2.40 (q, *J* = 7.1 Hz, 2H), 1.05 (t, *J* = 7.2 Hz, 3H). ^{13}C NMR (100 MHz, DMSO-d_6) δ: 165.6, 153.6, 143.7, 141.9, 141.3, 135.7, 131.5, 131.2, 129.9, 128.4, 125.0, 123.6, 122.9, 122.8, 119.7, 117.6, 113.9, 109.7, 52.6, 52.1, 47.4, 12.4. ESI-MS (*m/z*): $[M+H]^+$ = 562.2 (Calcd: 562.20). ESI-HRMS: calcd for $C_{28}H_{27}F_6N_5O$ $(M+H)^+$ 562.2042, found 562.2049. HPLC analysis: MeOH-H_2O (80: 20), 7.08 min, 95.9% purity.

***N*-(6-(3,5-dichlorophenyl)-1*H*-indazol-3-yl)-4-(4-ethylpiperazin-1-yl)benzamide (9k)** White solid: 28.9 mg, yield 41%; Mp 226.4-228.7 °C.^{1}H NMR (400 MHz, DMSO-d_6) δ 12.90 (brs, 1H), 10.58 (brs, 1H), 7.99 (t, *J* = 9.4 Hz, 2H), 7.81 (dd, *J* = 10.2, 5.0 Hz, 3H), 7.67-7.73 (m, 1H), 7.63 (t, *J* = 1.7 Hz, 1H), 7.16-7.47 (m, 1H), 7.03 (dd, *J* = 8.8, 4.5 Hz, 2H), 3.34 (s, 4H), 2.46-2.67 (m, 4H), 2.41 (s, 2H), 1.06 (t, *J* = 7.0 Hz, 3H). ^{13}C NMR (100 MHz, DMSO-d_6) δ: 165.5, 153.6, 144.6, 141.9, 136.0, 135.1, 129.8, 127.3, 126.4, 124.7, 123.5, 123.1, 120.2, 119.5, 117.5, 113.9, 113.1, 109.1, 52.5, 52.0, 47.3, 12.3. ESI-MS (*m/z*): $[M+H]^+$ = 494.2 (Calcd: 494.15). ESI-HRMS: calcd for $C_{26}H_{27}Cl_2N_5O$ $(M+H)^+$ 494.1514, found 494.1508. HPLC analysis: MeOH-H_2O (80: 20), 7.23 min, 96.5% purity.

***N*-(6-(2,5-dichlorophenyl)-1*H*-indazol-3-yl)-4-(4-ethylpiperazin-1-yl)benzamide (9l)** White solid: 30.3 mg, yield 39%; Mp 231.5-234.6 °C.^{1}H NMR (400 MHz, DMSO-d_6) δ 12.93 (brs, 1H), 10.59 (brs, 1H), 8.01 (d, *J* = 8.8 Hz, 2H), 7.79 (d, *J* = 8.5 Hz, 1H), 7.64 (d, *J* = 8.6 Hz, 1H), 7.58 (d, *J* = 2.5 Hz, 1H), 7.52 (dd, *J* = 8.3, 2.8 Hz, 2H), 7.09-7.15 (m, 1H), 7.05 (d, *J* = 8.8 Hz, 2H), 3.36-3.40 (m, 4H), 2.61-2.68 (m, 4H), 2.46-2.50 (q, *J* = 7.2 Hz, 2H), 1.10 (t, *J* = 6.8 Hz, 3H). ^{13}C NMR (100 MHz, DMSO-d_6) δ: 165.7, 153.4, 142.3, 141.3, 141.1, 136.0, 132.4, 131.9, 131.6, 130.9, 128.9, 129.5, 123.2, 122.5, 121.6, 117.1, 114.1, 111.2, 52.1, 51.9, 46.8, 11.6. ESI-MS (*m/z*): $[M+H]^+$ = 494.2 (Calcd: 494.15). ESI-HRMS: calcd for $C_{26}H_{27}Cl_2N_5O$ $(M+H)^+$ 494.1514, found 494.1510. HPLC analysis: MeOH-H_2O (80: 20), 7.36 min, 96.7% purity.

***N*-(6-(2,3-dichlorophenyl)-1*H*-indazol-3-yl)-4-(4-ethylpiperazin-1-yl)benzamide (9m)** White solid: 22.1 mg, yield 36%; Mp 220.1-222.4 °C.^{1}H NMR (400 MHz, DMSO-d_6) δ: 12.89 (brs, 1H), 10.58 (brs, 1H), 8.00 (d, *J* = 8.4 Hz, 2H), 7.78 (d, *J* = 8.0 Hz, 1H), 7.70-7.72 (m, 1H), 7.46-7.50 (m, 3H), 7.10 (d, *J* = 7.8 Hz, 1H), 7.04 (d, *J* = 8.4 Hz, 2H), 3.26-3.40 (m, 4H), 2.51-2.55 (m, 4H), 2.42 (q, *J* = 7.1 Hz, 2H), 1.05 (t, *J*

= 7.2 Hz, 3H). ^{13}C NMR (100 MHz, DMSO-d_6) δ: 165.6, 153.5, 143.1, 141.3, 141.2, 137.1, 132.8, 130.8, 130.4, 130.2, 129.8, 128.8, 123.0, 122.5, 121.6, 117.0, 114.0, 111.0, 52.4, 52.0, 47.2, 12.2. ESI-MS (*m/z*): $[M+H]^+$ = 494.2 (Calcd: 494.15). ESI-HRMS: calcd for $C_{26}H_{27}Cl_2N_5O$ $(M+H)^+$ 494.1514, found 494.1509. HPLC analysis: MeOH-H_2O (80: 20), 6.88 min, 95.5% purity.

***N*-(6-(3,4-dichlorophenyl)-1*H*-indazol-3-yl)-4-(4-ethylpiperazin-1-yl)benzamide (9n)** White solid: 21.8 mg, yield 38%; Mp 224.9-228.0 °C.^{1}H NMR (400 MHz, DMSO-d_6) δ: 12.91 (brs, 1H), 10.57 (brs, 1H), 7.99-8.03 (m, 3H), 7.73-7.81 (m, 3H), 7.53-7.60 (m, 1H), 7.40-7.47 (m, 1H), 7.04 (d, *J* = 8.4 Hz, 2H), 3.26-3.40 (m, 4H), 2.51-2.55 (m, 4H), 2.41 (q, *J* = 7.1 Hz, 2H), 1.06 (t, *J* = 7.2 Hz, 3H). ^{13}C NMR (100 MHz, DMSO-d_6) δ: 165.5, 153.6, 142.0, 141.7, 141.2, 136.3, 132.2, 131.5, 130.7, 129.8, 129.4, 127.9, 123.5, 122.9, 119.4, 117.3, 113.9, 108.7, 52.6, 52.1, 47.4, 12.4. ESI-MS (*m/z*): $[M+H]^+$ = 494.2 (Calcd: 494.15). ESI-HRMS: calcd for $C_{26}H_{27}Cl_2N_5O$ $(M+H)^+$ 494.1514, found 494.1514. HPLC analysis: MeOH-H_2O (80: 20), 7.05 min, 96.5% purity.

4-(4-ethylpiperazin-1-yl)-*N*-(6-(3-fluoro-5-methoxyphenyl)-1H-indazol-3-yl)benzamide (9o) White solid: 22.1 mg, yield 34%; Mp 225.5-227.0 °C. ^{1}H NMR (400 MHz, DMSO-d_6) δ: 12.86 (brs, 1H), 10.55 (brs, 1H), 8.00 (d, *J* = 8.9 Hz, 2H), 7.80 (d, *J* = 8.5 Hz, 1H), 7.72 (s, 1H), 7.40 (dd, *J* = 8.6, 1.3 Hz, 1H), 7.12-7.21 (m, 2H), 7.03 (d, *J* = 9.0 Hz, 2H), 6.85-6.89 (m, 1H), 3.87 (s, 3H), 3.26-3.41 (m, 4H), 2.51-2.57 (m, 4H), 2.39 (q, *J* = 7.1 Hz, 2H), 1.05 (t, *J* = 7.2 Hz, 3H). ^{13}C NMR (100 MHz, DMSO-d_6) δ: 165.0, 162.2, 161.1, 143.5, 141.5, 140.7, 137.2, 129.3, 122.8, 122.4, 119.2, 116.8, 113.4, 109.1, 108.1, 106.2, 106.0, 55.7, 52.1, 51.6, 46.9, 11.9. HRMS: calcd for $C_{27}H_{30}FN_5O_2$ $(M+H)^+$ 474.2305, found 474.2296. HPLC analysis: MeOH-H_2O (80: 20), 7.24 min, 96.7% purity.

***N*-(6-(2-chloro-4-(trifluoromethyl)phenyl)-1*H*-indazol-3-yl)-4-(4-ethylpiperazin-1-yl)benzamide (9p)** White solid: 19.9 mg, yield 35%; Mp 227.9-232.0 °C. ^{1}H NMR (400 MHz, DMSO-d_6) δ 12.92 (brs, 1H), 10.58 (brs, 1H), 7.97-8.04 (m, 3H), 7.80-7.85 (, 2H), 7.74 (d, *J* = 8.0 Hz, 1H), 7.55 (s, 1H), 7.14 (dd, *J* = 8.5, 1.2 Hz, 1H), 7.03 (d, *J* = 8.9 Hz, 2H), 3.33-3.40 (m, 4H), 2.48-2.52 (m, 4H), 2.45 (s, 2H), 1.06 (t, *J* = 7.1 Hz, 3H). ^{13}C NMR (100 MHz, DMSO-d_6) δ: 165.7, 153.5, 144.8, 141.3, 136.0, 133.2, 133.0, 129.9, 127.2, 124.8, 123.0, 122.7, 121.4, 117.2, 114.0, 111.2, 52.4, 52.0, 47.1, 12.1. ESI-MS (*m/z*): $[M+H]^+$ = 528.1 (Calcd: 528.17). ESI-HRMS: calcd for $C_{27}H_{27}ClF_3N_5O$

$(M+H)^+$ 528.1778, found 528.1777. HPLC analysis: MeOH-H_2O (80: 20), 7.18 min, 96.9% purity.

***N*-(6-(2,6-dimethylphenyl)-1*H*-indazol-3-yl)-4-(4-ethylpiperazin-1-yl)benzamide (9q)** White solid: 30.9 mg, yield 40%; Mp 235.7-239.2 °C.^{1}H NMR (400 MHz, DMSO-d_6) δ 12.75 (brs, 1H), 10.56 (brs, 1H), 8.02 (d, J = 8.7 Hz, 2H), 7.77 (d, J = 8.3 Hz, 1H), 7.18 (d, J = 8.4 Hz, 2H), 7.14 (d, J = 8.2 Hz, 2H), 7.07 (d, J = 8.6 Hz, 2H), 6.82 (d, J = 8.3 Hz, 1H), 3.37-3.43 (m, 4H), 2.54-2.68 (m, 4H), 2.35 (q, J = 7.2 Hz, 2H), 2.01 (s, 6H), 1.14 (t, J = 7.0 Hz, 3H). ^{13}C NMR (100 MHz, DMSO-d_6) δ: 165.6, 153.0, 142.0, 141.1, 139.1, 135.8, 129.9, 127.8, 127.5, 122.8, 121.5, 116.5, 114.3, 110.2, 51.7, 51.6, 46.4, 21.0, 12.2. ESI-MS (m/z): $[M+H]^+$ = 454.3 (Calcd: 454.26). ESI-HRMS: calcd for $C_{26}H_{28}ClN_5O$ $(M+H)^+$ 454.2607, found 454.2606. HPLC analysis: MeOH-H_2O (80: 20), 7.30 min, 95.7% purity.

4-(4-ethylpiperazin-1-yl)-*N*-(6-(2-(trifluoromethoxy)phenyl)-1*H*-indazol-3-yl)benzamide (9r) White solid: 28.2 mg, yield 38%; Mp 230.9-233.5 °C. ^{1}H NMR (400 MHz, DMSO-d_6) δ: 12.87 (brs, 1H), 10.57 (brs, 1H), 8.00 (d, J = 8.4 Hz, 2H), 7.79 (d, J = 7.8 Hz, 1H), 7.62-7.64 (m, 1H), 7.53-7.57 (m, 4H), 7.16 (d, J = 8.0 Hz, 1H), 7.04 (d, J = 8.8 Hz, 2H), 3.28-3.43 (m, 4H), 2.47-2.53 (m, 4H), 2.41 (q, J = 7.1 Hz, 2H), 1.06 (t, J = 7.2 Hz, 3H). ^{13}C NMR (100 MHz, DMSO-d_6) δ: 165.6, 153.6, 145.9, 141.6, 141.2, 135.5, 134.7, 132.4, 129.9, 128.5, 123.0, 122.6, 121.5, 117.0, 113.9, 110.8, 52.5, 52.0, 47.4, 12.3. ESI-MS (m/z): $[M+H]^+$ = 510.2 (Calcd: 510.21). ESI-HRMS: calcd for $C_{27}H_{28}F_3N_5O_2$ $(M+H)^+$ 510.2117, found 510.2119. HPLC analysis: MeOH-H_2O (80: 20), 7.18 min, 96.9% purity.

***N*-(6-(4-cyanophenyl)-1*H*-indazol-3-yl)-4-(4-ethylpiperazin-1-yl)benzamide (9s)** White solid: 15.8 mg, yield 38%; Mp 226.4-230.0 °C. ^{1}H NMR (400 MHz, DMSO-d_6) δ 12.94 (brs, 1H), 10.52 (brs, 1H), 7.93-8.08 (m, 6H), 7.85 (d, J = 8.5 Hz, 1H), 7.78 (s, 1H), 7.44 (d, J = 8.6 Hz, 1H), 7.03 (d, J = 8.6 Hz, 2H), 3.28-3.39 (m, 4H), 2.51-2.59 (m, 4H), 2.40 (q, J = 6.7 Hz, 2H), 1.06 (t, J = 7.1 Hz, 3H). ^{13}C NMR (100 MHz, DMSO-d_6) δ: 165.6, 153.7, 145.6, 142.1, 141.2, 137.1, 133.3, 129.9, 128.5, 123.6, 122.9, 119.6, 117.5, 113.9, 110.5, 109.1, 52.5, 52.1, 47.4, 12.3. ESI-MS (m/z): $[M+H]^+$ = 451.2 (Calcd: 451.22). ESI-HRMS: calcd for $C_{27}H_{28}N_6O$ $(M+H)^+$ 451.2246, found 451.2238. HPLC analysis: MeOH-H_2O (80: 20), 7.40 min, 96.3% purity.

4-(4-ethylpiperazin-1-yl)-*N*-(6-(1-methyl-1*H*-pyrazol-3-yl)-1*H*-indazol-3-yl) benzamide (9t) White solid: 18.7 mg, yield 35%; Mp 221.6-224.2 °C. ^{1}H NMR (400 MHz, DMSO-d_6) δ 12.67 (brs, 1H), 10.46 (brs, 1H), 8.23 (s, 1H), 7.98 (d, J = 8.8 Hz,

2H), 7.94 (s, 1H), 7.68 (d, *J* = 8.5 Hz, 1H), 7.57 (s, 1H), 7.30 (d, *J* = 8.5 Hz, 1H), 7.02 (d, *J* = 8.8 Hz, 2H), 3.89 (s, 3H), 3.37-3.45 (m, 4H), 2.52-2.60 (m, 4H), 2.41 (q, *J* = 6.7 Hz, 2H), 1.06 (t, *J* = 7.2 Hz, 3H). ^{13}C NMR (100 MHz, DMSO-d_6) δ: 165.6, 153.6, 142.4, 141.1, 136.8, 131.3, 129.8, 128.7, 123.0, 122.7, 118.6, 116.1, 113.9, 105.5, 52.5, 52.0, 47.3, 19.0, 12.3. ESI-MS (*m/z*): [M+H]$^+$ = 430.2 (Calcd: 430.24). ESI-HRMS: calcd for $C_{24}H_{29}N_7O$ (M+H)$^+$ 430.2355, found 430.2357. HPLC analysis: MeOH-H_2O (80: 20), 7.11 min, 95.5% purity.

4-(4-(dimethylamino)piperidin-1-yl)-*N*-(6-(3-fluoro-5-methoxyphenyl)-1*H*-indazol-3-yl)benzamide (9u) White solid: 14.3 mg, yield 46%; Mp 223.8-227.4 °C. ^{1}H NMR (400 MHz, DMSO-d_6) δ 12.87 (brs, 1H), 10.54 (brs, 1H), 7.99 (d, *J* = 8.7 Hz, 2H), 7.79 (d, *J* = 8.5 Hz, 1H), 7.72 (s, 1H), 7.40 (d, *J* = 8.8 Hz, 1H), 7.11-7.22 (m, 2H), 7.04 (d, *J* = 8.8 Hz, 2H), 6.87 (d, *J* = 10.9 Hz, 1H), 3.98 (d, *J* = 12.2 Hz, 2H), 3.87 (s, 3H), 2.82-2.90 (m, 2H), 2.59 (s, 1H), 2.35 (s, 6H), 1.92 (s, 2H), 1.51 (d, *J* = 10.8 Hz, 2H). ^{13}C NMR (100 MHz, DMSO-d_6) δ: 165.5, 164.8, 162.9, 161.6, 153.2, 143.9, 142.0, 141.2, 137.7, 129.9, 123.3, 122.5, 119.6, 117.3, 114.1, 109.6, 108.6, 106.7, 100.9, 62.1, 56.2, 46.9, 41.3, 27.4, 21.6. ESI-MS (*m/z*): [M+H]$^+$ = 488.3 (Calcd: 488.26). HRMS: calcd for $C_{27}H_{30}FN_5O_2$ (M+H)$^+$ 488.2462, found 488.2459. HPLC analysis: MeOH-H_2O (80: 20), 7.22 min, 97.5% purity.

4-((*3R,5S*)-3,5-dimethylpiperazin-1-yl)-*N*-(6-(3-fluoro-5-methoxyphenyl)-1*H*-indazol-3-yl)benzamide (9v) White solid: 26.5 mg, yield 38%; Mp 229.6-233.4 °C. ^{1}H NMR (400 MHz, DMSO-d_6) δ 12.87 (brs, 1H), 10.56 (brs, 1H), 7.99 (d, *J* = 8.8 Hz, 2H), 7.79 (d, *J* = 8.6 Hz, 1H), 7.72 (s, 1H), 7.40 (d, *J* = 8.5 Hz, 1H), 7.14-7.18 (m, 2H), 7.04 (d, *J* = 9.0 Hz, 2H), 6.87 (d, *J* = 10.9 Hz, 1H), 4.47 (brs, 1H), 3.87 (s, 3H), 3.82 (s, 2H), 2.96 (s, 2H), 2.36 (d, *J* = 10.7 Hz, 2H), 1.11 (d, *J* = 6.1 Hz, 6H). ^{13}C NMR (100 MHz, DMSO-d_6) δ: 165.5, 164.8, 162.9, 161.6, 153.2, 143.9, 142.0, 141.1, 137.7, 129.9, 126.7, 123.3, 119.6, 117.3, 113.9, 109.6, 108.6, 106.7, 100.9, 63.3, 56.2, 53.3, 50.6, 19.0. ESI-MS (*m/z*): [M+H]$^+$ = 474.2 (Calcd: 474.23). ESI-HRMS: calcd for $C_{27}H_{30}FN_5O_2$ (M+H)$^+$ 474.2305, found 474.2290. HPLC analysis: MeOH-H_2O (80: 20), 7.11 min, 95.5% purity.

***N*-(6-(3-fluoro-5-methoxyphenyl)-1*H*-indazol-3-yl)-4-(4-methyl-1,4-diazepan-1-yl)benzamide (9w)** White solid: 30.1 mg, yield 33%; Mp 230.4-234.6 °C. ^{1}H NMR (400 MHz, DMSO-d_6) δ 12.83 (brs, 1H), 10.44 (brs, 1H), 7.97 (d, *J* = 8.4 Hz, 2H), 7.79 (d, *J* = 8.0 Hz, 1H), 7.71 (s, 1H), 7.39 (d, *J* = 8.1 Hz, 1H), 7.14-7.18 (m, 2H), 6.85-6.89 (m, 1H), 6.80 (d, *J* = 7.9 Hz, 2H), 3.87 (s, 7H), 3.63-3.65 (m, 2H), 3.53 (t, *J* = 6.8 Hz,

2H), 2.70-2.78 (m, 2H), 2.55-2.62 (m, 2H), 2.48-2.51 (m, 4H), 2.36 (s, 3H). ^{13}C NMR (100 MHz, DMSO-d_6) δ: 165.6, 164.8, 162.9, 161.6, 151.7, 144.0, 142.0, 137.6, 130.16, 123.3, 120.2, 119.6, 117.3, 110.8, 109.6, 108.6, 106.7, 101.1, 57.2, 56.6, 56.2, 48.1, 46.1, 26.8. ESI-MS (*m/z*): $[M+H]^+$ = 474.0(Calcd: 473.22). ESI-HRMS: calcd for $C_{27}H_{30}FN_5O_2$ $(M+H)^+$ 474.2305, found 474.2297. HPLC analysis: MeOH-H_2O (80: 20), 7.15 min, 96.2% purity.

***N*-(6-(3-fluoro-5-methoxyphenyl)-1*H*-indazol-3-yl)-4-((4-methylpiperazin-1-yl)methyl)benzamide (9x)** White solid: 20.1 mg, yield 44%; Mp 240.3-242.5 °C. ^{1}H NMR (400 MHz, DMSO-d_6) δ: 13.01 (brs, 1H), 10.84 (brs, 1H), 8.06 (d, *J* = 8.2 Hz, 2H), 7.80 (d, *J* = 8.2 Hz, 1H), 7.74 (s, 1H), 7.46 (d, *J* = 8.0 Hz, 2H), 7.41 (dd, *J* = 8.0, 1.4Hz, 1H), 7.14-7.18 (m, 2H), 6.85-6.89 (m, 1H), 3.87 (s, 1H), 3.55 (s, 2H), 2.33-2.46 (m, 8H), 2.16 (s, 3H). ^{13}C NMR (100 MHz, DMSO-d_6) δ: 165.9, 164.8, 162.9, 161.6, 144.0, 143.0, 142.0, 140.6, 137.7, 132.9, 130.1, 129.1, 128.4, 123.0, 119.8, 117.1, 109.6, 108.7, 106.7, 100.9, 62.1, 56.2, 55.2, 53.1, 46.2. ESI-MS (*m/z*): $[M+H]^+$ = 474.2 (Calcd: 474.23). ESI-HRMS: calcd for $C_{27}H_{30}FN_5O_2$ $(M+H)^+$ 474.2305, found 474.2298. HPLC analysis: MeOH-H_2O (80: 20), 7.11 min, 95.5% purity.

***N*-(6-(3-fluoro-5-methoxyphenyl)-1*H*-indazol-3-yl)-4-(4-methylpiperazin-1-yl)benzamide (9y)** White solid: 17.1 mg, yield 30%; Mp 237.4-241.6 °C. ^{1}H NMR (400 MHz, DMSO-d_6) δ: 13.01 (brs, 1H), 10.84 (brs, 1H), 8.06 (d, *J* = 8.2 Hz, 2H), 7.80 (d, *J* = 8.2 Hz, 1H), 7.74 (s, 1H), 7.46 (d, *J* = 8.0 Hz, 2H), 7.41 (dd, *J* = 8.0, 1.4Hz, 1H), 7.14-7.18 (m, 2H), 6.85-6.89 (m, 1H), 3.87 (s, 1H), 3.24-3.39 (s, 4H), 2.44-2.52 (s, 4H), 2.16 (s, 3H). ^{13}C NMR (100 MHz, DMSO-d_6) δ: 165.9, 164.7, 163.1, 161.6, 144.0, 143.0, 142.0, 140.7, 137.7, 132.9, 129.1, 128.4, 123.0, 119.8, 117.2, 109.6, 108.7, 106.7, 101.1, 62.1, 56.2, 55.2, 53.1, 46.2. ESI-MS (*m/z*): $[M+H]^+$ = 460.2 (Calcd: 460.21). ESI-HRMS: calcd for $C_{27}H_{30}FN_5O_2$ $(M+H)^+$ 460.2149, found 460.2155. HPLC analysis: MeOH-H_2O (80: 20), 7.42 min, 96.4% purity.

***N*-(6-(3-fluoro-5-methoxyphenyl)-1*H*-indazol-3-yl)-5-(4-methylpiperazin-1-yl)pyrazine-2-carboxamide (9z)** White solid: 19.8 mg, yield 35%; Mp 237.7-240.3 °C. ^{1}H NMR (400 MHz, DMSO-d_6) δ: 12.95 (brs, 1H), 10.42 (brs, 1H), 8.80 (s, 1H), 8.44 (s, 1H), 7.94 (d, *J* = 8.4 Hz, 1H), 7.74 (s, 1H), 7.42 (d, *J* = 8.3 Hz, 1H), 7.05-7.25 (m, 2H), 6.87 (d, *J* = 10.9 Hz, 1H), 3.87 (s, 7H), 2.70 (s, 4H), 2.42 (s, 3H). ESI-MS (*m/z*): $[M+H]^+$=462.2 (Calcd: 462.21). ^{13}C NMR (100 MHz, DMSO-d_6) δ: 164.8, 162.8, 161.6, 155.6, 142.8 142.1, 140.1, 137.8, 132.4, 130.1, 129.5, 123.2, 119.8, 116.7 109.6, 108.6, 106.7, 101.0, 56.2, 54.5, 46.0, 44.2. ESI-MS (*m/z*): $[M+H]^+$ = 462.2 (Calcd: 462.20).

HRMS: calcd for $C_{24}H_{25}FN_7O_2$ $(M+H)^+$ 462.2054, found 462.2038. HPLC analysis: MeOH-H_2O (80: 20), 7.11 min, 95.5% purity.

***N*-(6-bromobenzo[d]thiazol-2-yl)-4-(4-methylpiperazin-1-yl)benzamide(11)** A mixture of 2-amino-6-bromobenzothiazole (1.15 g, 5 mmol), sodium hydride (60%, 0.99 g 25 mmol) and compound **4** (2.03 g, 6 mmol) in THF (30 mL) was stirred at 60°C for 30min. The reaction annihilated with 1mL H_2O and concentrated in *vacuo*. The residue was dissolved in CH_2Cl_2 (100 mL) and washed with $NaHCO_3$ (50 mL) and brine(50 mL), dried over Na_2SO_4, and concentrated in *vacuo*. The residue was purified by chromatography on silica gel using CH_2Cl_2-MeOH(25:1) to compound **11** as white solid (1.19 g, 55%). Mp 200.6-202.2 °C. ^1H NMR (500 MHz, DMSO-d_6) δ: 12.60 (s, 1H), 8.27 (s, 1H), 8.05 (d, J = 8.5 Hz, 2H), 7.69 (d, J = 8.5 Hz, 1H), 7.59 (d, J = 8.5 Hz, 1H), 7.03 (d, J = 8.6 Hz, 2H), 2.45 (s, 4H), 2.23 (s, 3H). ^{13}C NMR (125 MHz, DMSO-d_6) δ: 165.7, 160.4, 154.2, 148.3, 134.3, 130.5, 129.5, 124.6, 122.2, 120.1, 115.7, 113.7, 54.8, 46.9, 46.2. HRMS: calcd for $C_{19}H_{20}BrN_4OS$ $(M+H)^+$ 431.0463, found 431.0476.

***N*-(6-(3-methoxyphenyl)benzo[d]thiazol-2-yl)-4-(4-methylpiperazin-1-yl) benzamide (12a)** The compound **11** (130.2mg, 0.3mmol) was dissolved in dioxane (10 mL) and H_2O (5 mL), then followed by the additions of 3-methoxyphenylboronic acid (70.2 mg, 0.44 mmol), Pd(dppf)Cl_2 (20 mg), and Cs_2CO_3 (95.4 mg, 0.91 mmol). The reaction mixture was heated at 120 °C for 3 h before cooled to rt. After concentrated, the residue was dissolved in EtOAc (50 mL) and washed with H_2O (20 mL), and brine (20 mL), dried over Na_2SO_4, and concentrated in *vacuo*. The residue was purified by chromatography on silica gel using CH_2Cl_2-MeOH (30:1) to give **12a** (73.7 mg, 53%) as a white solid. Mp 230.5-233.2 °C. ^1H NMR (500 MHz, DMSO-d_6) δ: 12.57 (brs, 1H), 8.34 (s, 1H), 8.07 (d, J = 8.8 Hz, 2H), 7.81 (d, J = 8.4 Hz, 1H), 7.76 (d, J = 8.3 Hz, 1H), 7.40 (t, J = 7.9 Hz, 1H), 7.31 (d, J = 7.7 Hz, 1H), 7.28 (s, 1H), 7.03 (d, J = 8.9 Hz, 2H), 6.95 (d, J = 8.0 Hz, 1H), 3.85 (s, 3H), 2.44 (s, 4H), 2.23 (s, 3H). ^{13}C NMR (125 MHz, DMSO-d_6) δ 165.57, 160.24, 160.06, 154.19, 148.63, 141.98, 135.97, 132.96, 130.46, 130.43, 125.64, 120.78, 120.29, 120.19, 119.59, 113.71, 113.34, 112.70, 55.62, 54.77, 46.94, 46.18. HRMS: calcd for $C_{26}H_{27}N_4O_2S$ $(M+H)^+$ 459.1776, found 459.1708. HPLC analysis: MeOH-H_2O (85: 15), 7.50 min, 97.1% purity.

***N*-(6-(3,5-dimethoxyphenyl)benzo[d]thiazol-2-yl)-4-(4-methylpiperazin-1-yl) benzamide (12b)** White solid 56.7 mg, yield 49%. Mp 234.6-238.2 °C. ^1H NMR (500 MHz, DMSO-d_6) δ 12.58 (brs, 1H), 8.34 (s, 1H), 8.06 (d, J = 8.3 Hz, 2H), 7.73-7.84 (m, 2H), 7.03 (d, J = 8.3 Hz, 2H), 6.88 (s, 2H), 6.51 (s, 1H), 3.84 (s, 6H), 2.44 (s, 4H),

2.23 (s, 3H). ^{13}C NMR (125 MHz, DMSO-d_6) δ 165.57, 161.33, 160.09, 154.19, 148.72, 142.62, 136.02, 132.89, 130.43, 125.67, 120.69, 120.29, 120.25, 113.70, 105.39, 99.68, 55.77, 54.77, 46.94, 46.17. HRMS: calcd for $C_{27}H_{29}N_4O_3S$ $(M+H)^+$ 489.1882, found 489.1852. HPLC analysis: MeOH-H_2O (85: 15), 7.70 min, 96.4% purity.

4-(4-methylpiperazin-1-yl)-*N*-(6-(3,4,5-trimethoxyphenyl)benzo[d]thiazol-2-yl)benzamide (12c) White solid 50.5 mg, yield 47%. Mp 236.4-238.3 °C. ^1H NMR (500 MHz, DMSO-d_6) δ 12.59 (brs, 1H), 8.17 (s, 1H), 8.07 (d, *J* = 8.8 Hz, 2H), 7.83 (d, *J* = 8.4 Hz, 1H), 7.60 (d, *J* = 8.3 Hz, 1H), 7.24 (t, *J* = 7.9 Hz, 1H), 7.19 (t, *J* = 7.5 Hz, 1H), 7.12 (t, *J* = 6.6 Hz, 1H), 7.04 (d, *J* = 8.9 Hz, 2H), 3.90 (s, 3H), 2.45 (s, 4H), 2.23 (s, 3H). ^{13}C NMR (125 MHz, DMSO-d_6) δ 165.57, 159.85, 154.19, 153.68, 148.40, 137.43, 136.32, 136.26, 132.86, 130.43, 125.67, 120.63, 120.30, 120.08, 113.71, 104.76, 60.54, 56.46, 54.77, 46.94, 46.18. HRMS: calcd for $C_{28}H_{31}N_4O_4S$ $(M+H)^+$ 519.1988, found 519.2007. HPLC analysis: MeOH-H_2O (85: 15), 7.35 min, 96.8% purity.

***N*-(6-(2-fluoro-3-methoxyphenyl)benzo[d]thiazol-2-yl)-4-(4-methylpiperazin-1-yl)benzamide (12d)** White solid 64.8 mg, yield 52%. Mp 232.5-235.2 °C. ^1H NMR (500 MHz, DMSO-d_6) δ 12.59 (brs, 1H), 8.17 (s, 1H), 8.07 (d, *J* = 8.8 Hz, 2H), 7.83 (d, *J* = 8.4 Hz, 1H), 7.60 (d, *J* = 8.3 Hz, 1H), 7.24 (t, *J* = 7.9 Hz, 1H), 7.19 (t, *J* = 7.5 Hz, 1H), 7.12 (t, *J* = 6.6 Hz, 1H), 7.04 (d, *J* = 8.9 Hz, 2H), 3.90 (s, 3H), 2.45 (s, 4H), 2.23 (s, 3H). ^{13}C NMR (125 MHz, DMSO-d_6) δ 165.59, 160.39, 154.19, 150.12, 148.69, 148.32, 148.23, 148.17, 132.55, 130.65, 130.44, 129.38, 129.29, 127.56, 127.54, 125.01, 124.97, 122.36, 122.28, 120.49, 120.24, 113.70, 113.24, 56.58, 54.75, 46.92, 46.15. HRMS: calcd for $C_{26}H_{26}FN_4O_2S$ $(M+H)^+$ 477.1682, found 477.1653. HPLC analysis: MeOH-H_2O (85: 15), 7.58 min, 95.5% purity.

4-(4-methylpiperazin-1-yl)-*N*-(5-phenethyl-1*H*-1,2,4-triazol-3-yl)benzamide (18a) A mixture of the compound **17a** (350.6 mg, 1.0 mmol), sodium hydride (119.6 mg, 5.0 mmol) and **4** (406.2 mg, 1.2 mmol) in THF (30 mL) was stirred at 60 °C for 30 min. After reaction annihilated with 1mL H_2O, the suspension was concentrated and the residue was dissolved in CH_2Cl_2 (100 mL) and washed with brine dried over Na_2SO_4. The solution was concentrated in *vacuo* and the residue was dissolved in THF (20mL) and TFA (10 mL). The reaction mixture was stirred for 2 h and monitored by TLC. After concentrated, the residue was dissolved in EtOAc (100 mL) and washed with $NaHCO_3$ (50 mL) and brine (50 mL), dried over Na_2SO_4, and concentrated in *vacuo*. The residue was purified by chromatography on silica gel using CH_2Cl_2-MeOH(20:1) to compound **18c** as white solid (145.4 mg, 32%). Mp 218.1-222.5 °C. ^1H NMR (500

MHz, DMSO-d_6) δ 13.14 (brs, 1H), 11.59 (brs, 1H), 7.96 (d, J = 7.7 Hz, 2H), 7.24-7.28 (m, 4H), 7.18 (t, J = 7.1 Hz, 1H), 7.00 (d, J = 8.9 Hz, 2H), 3.31 (s, 4H), 3.00 (t, J = 7.7 Hz, 2H), 2.88 (s, 2H), 2.37-2.48 (m, 4H), 2.22 (s, 3H). ^{13}C NMR (125 MHz, DMSO-d_6) δ 165.29, 159.70, 153.93, 130.05, 129.74, 120.96, 114.42, 113.77, 111.80, 55.33, 54.80, 47.11, 46.21, 34.05, 30.09. HRMS: calcd for $C_{24}H_{31}N_6O_3$ $(M+H)^+$ 391.2168, found 391.2083. HPLC analysis: MeOH-H_2O (85: 15), 9.05 min, 97.5% purity.

***N*-(5-(3-methoxyphenethyl)-1*H*-1,2,4-triazol-3-yl)-4-(4-methylpiperazin-1-yl) benzamide (18b)** White solid 82.1 mg, yield 31%. Mp 216.4-219.5 °C. ^{1}H NMR (500 MHz, DMSO-d_6) δ 13.09 (brs, 1H), 11.55 (brs, 1H), 7.96 (d, J = 5.2 Hz, 2H), 7.19 (t, J = 7.8 Hz, 1H), 7.00 (d, J = 8.9 Hz, 2H), 6.81 (d, J = 7.6 Hz, 2H), 6.75 (d, J = 8.4 Hz, 1H), 3.72 (s, 3H), 3.31 (s, 4H), 2.97 (t, J = 7.5 Hz, 2H), 2.88 (d, J = 8.6 Hz, 2H), 2.37-2.47 (m, 4H), 2.23 (s, 3H). ^{13}C NMR (125 MHz, DMSO-d_6) δ 165.29, 159.70, 130.05, 129.74, 120.96, 114.42, 113.77, 111.80, 55.33, 54.80, 47.11, 46.21, 34.05. HRMS: calcd for $C_{23}H_{29}N_6O_2$ $(M+H)^+$ 421.2274, found 421.2215. HPLC analysis: MeOH-H_2O (85: 15), 8.80 min, 97.2% purity.

***N*-(5-(3,5-dimethoxyphenethyl)-1*H*-1,2,4-triazol-3-yl)-4-(4-methylpiperazin-1-yl)benzamide (18c)**. White solid 77.8 mg, yield 31%. Mp 220.9-223.3 °C. ^{1}H NMR (500 MHz, DMSO-d_6) δ 13.11 (brs, 1H), 11.60 (brs, 1H), 7.96 (s, 2H), 6.94-7.05 (m, 2H), 6.38-6.41 (m, 2H), 6.31 (s, 1H), 3.71 (s, 6H), 3.31 (s, 4H), 2.79-2.97 (m, 4H), 2.40-2.47 (m, 4H), 2.22 (s, 3H). HRMS: calcd for $C_{24}H_{31}N_6O_3$ $(M+H)^+$ 451.2379, found 451.2351. HPLC analysis: MeOH-H_2O (85: 15), 8.72 min, 96.9% purity.

Reference

1. Tucker JA, Klein T, Breed J, et al. Structural Insights into FGFR Kinase Isoform Selectivity: Diverse Binding Modes of AZD4547 and Ponatinib in Complex with FGFR1. Structure 2014; 22:1764-74.
2. Klein T, Vajpai N, Phillips J, et al. Structural and dynamic insights into the energetics of activation loop rearrangement in FGFR1 kinase. Nat Commun 2015; 6:7877-88.
3. Nakanishi Y, Akiyama N, Tsukaguchi T, et al. The Fibroblast Growth Factor Receptor Genetic Status as a Potential Predictor of the Sensitivity to CH5183284/Debio 1347, a Novel Selective FGFR Inhibitor. Mol Cancer Ther 2014; 13:2547-58.
4. Liu J, Peng X, Dai Y, et al. Design, Synthesis and Biological Evaluation of Novel FGFR Inhibitors Bearing Indazole Scaffold. Org Biomol Chem 2015; 13:7643-54.
5. Guagnano V, Furet P, Spanka C, et al. Discovery of 3-(2,6-Dichloro-3,5-dimethoxy-phenyl)-1-

{6-[4-(4-ethyl-piperazin-1-yl)-phenylamino]-pyrimidin-4-yl}-1-methyl-urea (NVP-BGJ398), A Potent and Selective Inhibitor of the Fibroblast Growth Factor Receptor Family of Receptor Tyrosine Kinase. J Med Chem 2011; 54:7066-83.

6. Patani H, Bunney TD, Thiyagarajan N, et al. Landscape of activating cancer mutations in FGFR kinases and their differential responses to inhibitors in clinical use. Oncotarget 2016; 7:24252-68.

7. Matsuki M, Hoshi T, Yamamoto Y, et al. Lenvatinib inhibits angiogenesis and tumor fibroblast growth factor signaling pathways in human hepatocellular carcinoma models. Cancer Med 2018; 7:2641-53.

^{1}H NMR and ^{13}C NMR of compound **9a**

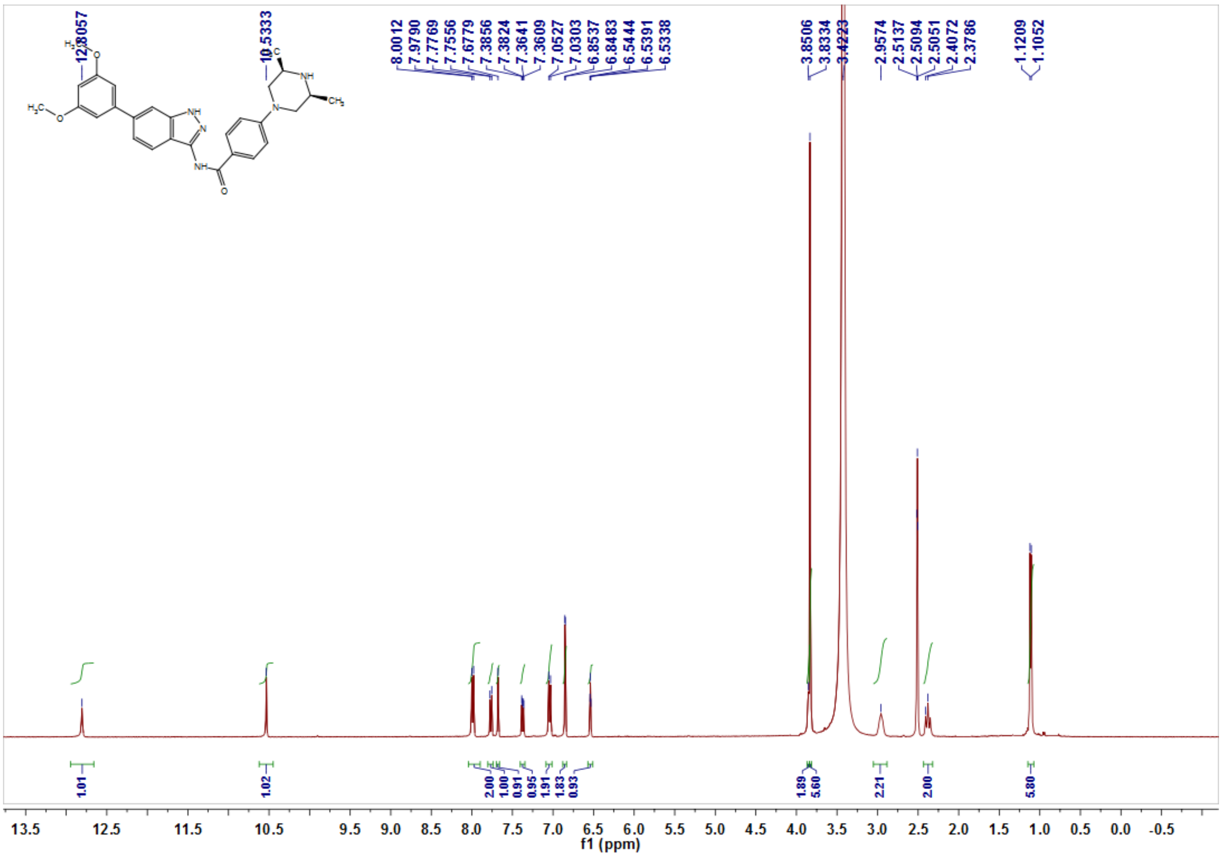

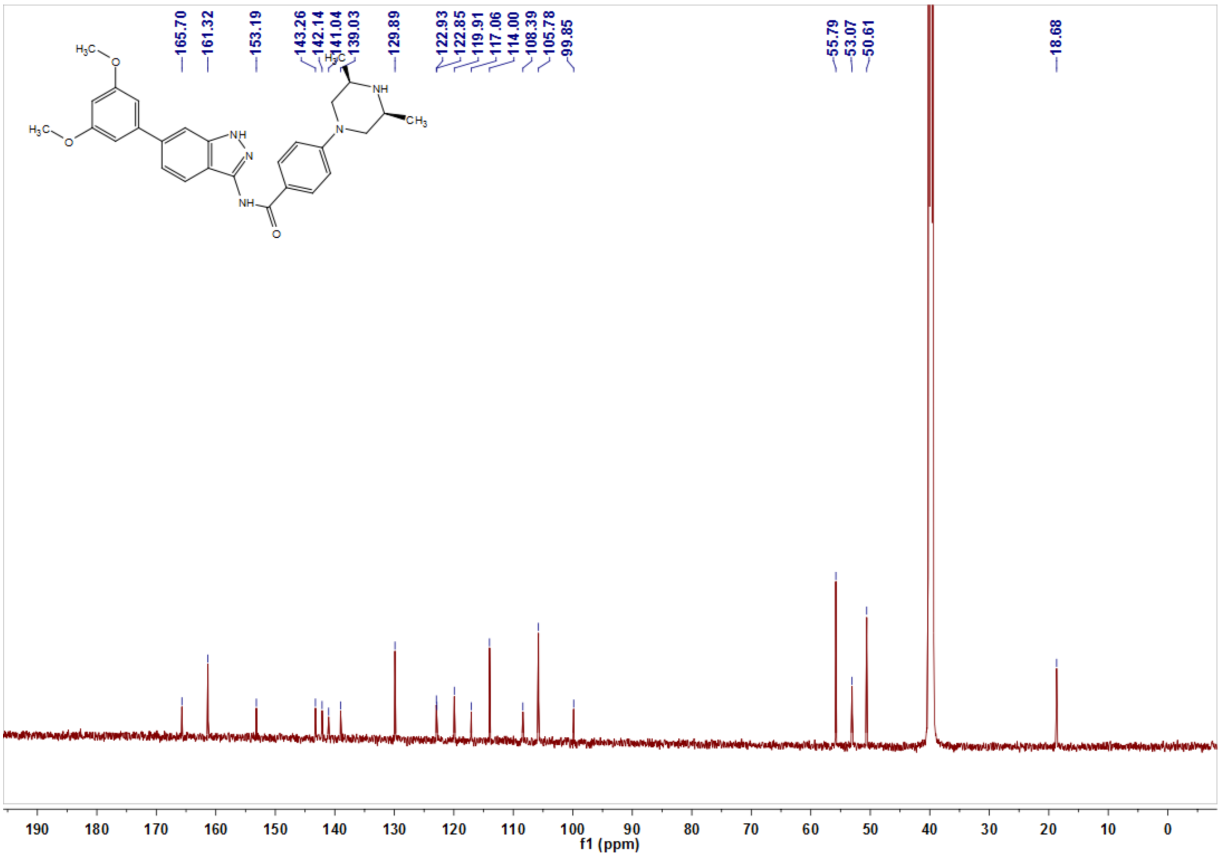

^{1}H NMR and ^{13}C NMR of compound **9b**

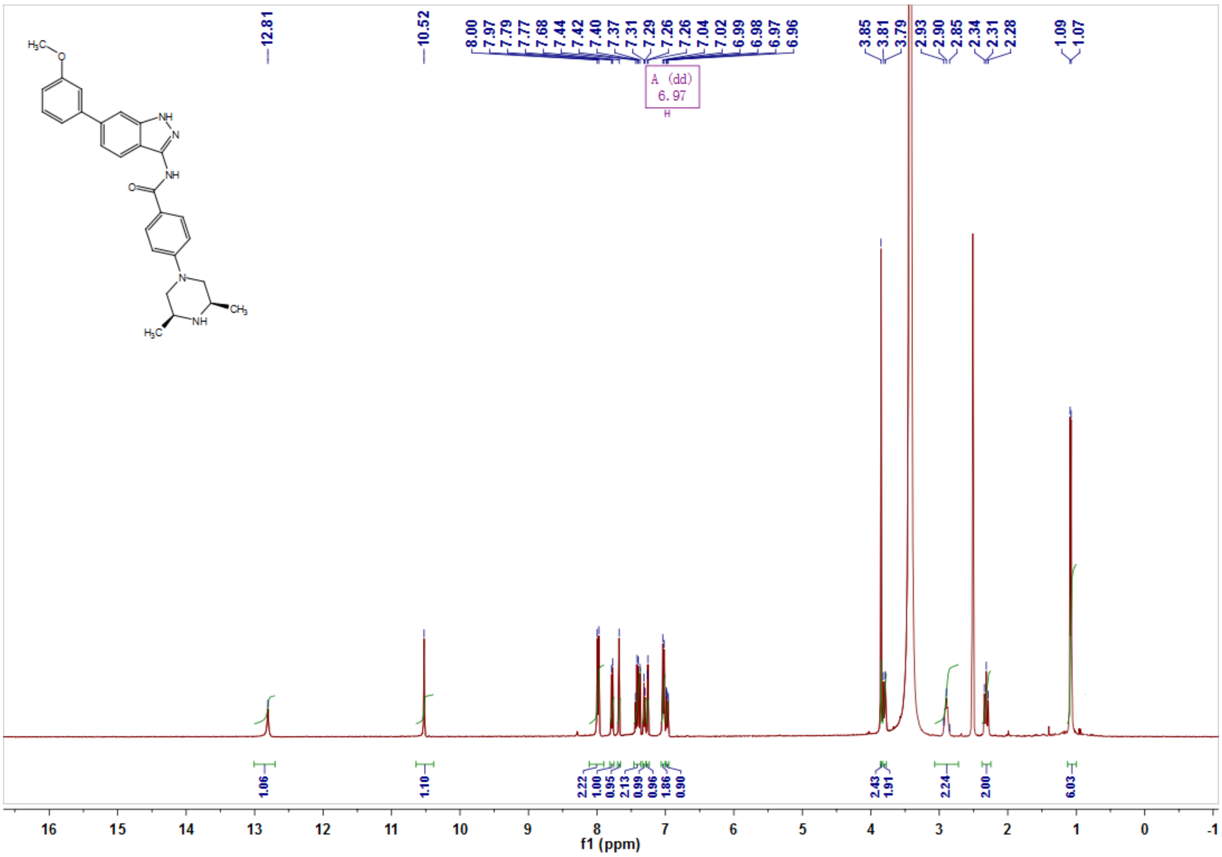

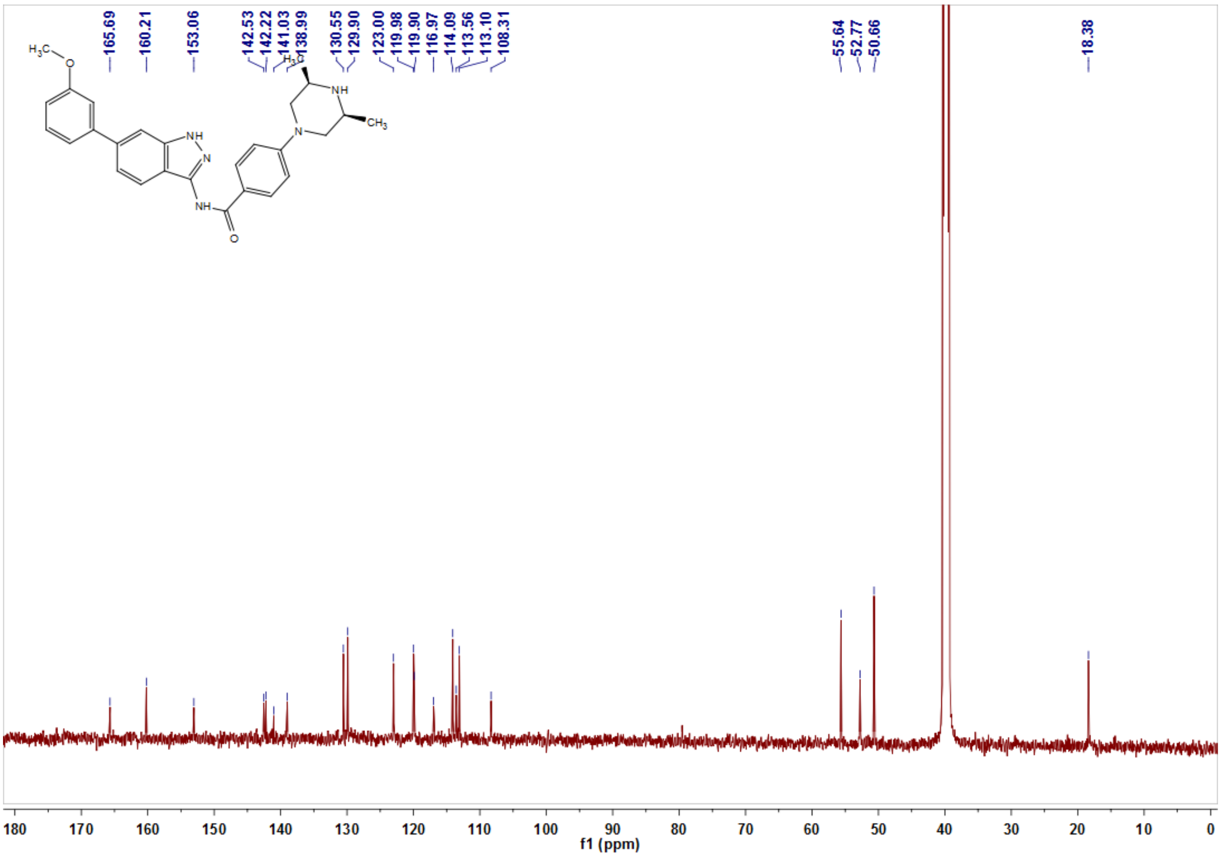

^1H NMR and ^{13}C NMR of compound **9d**

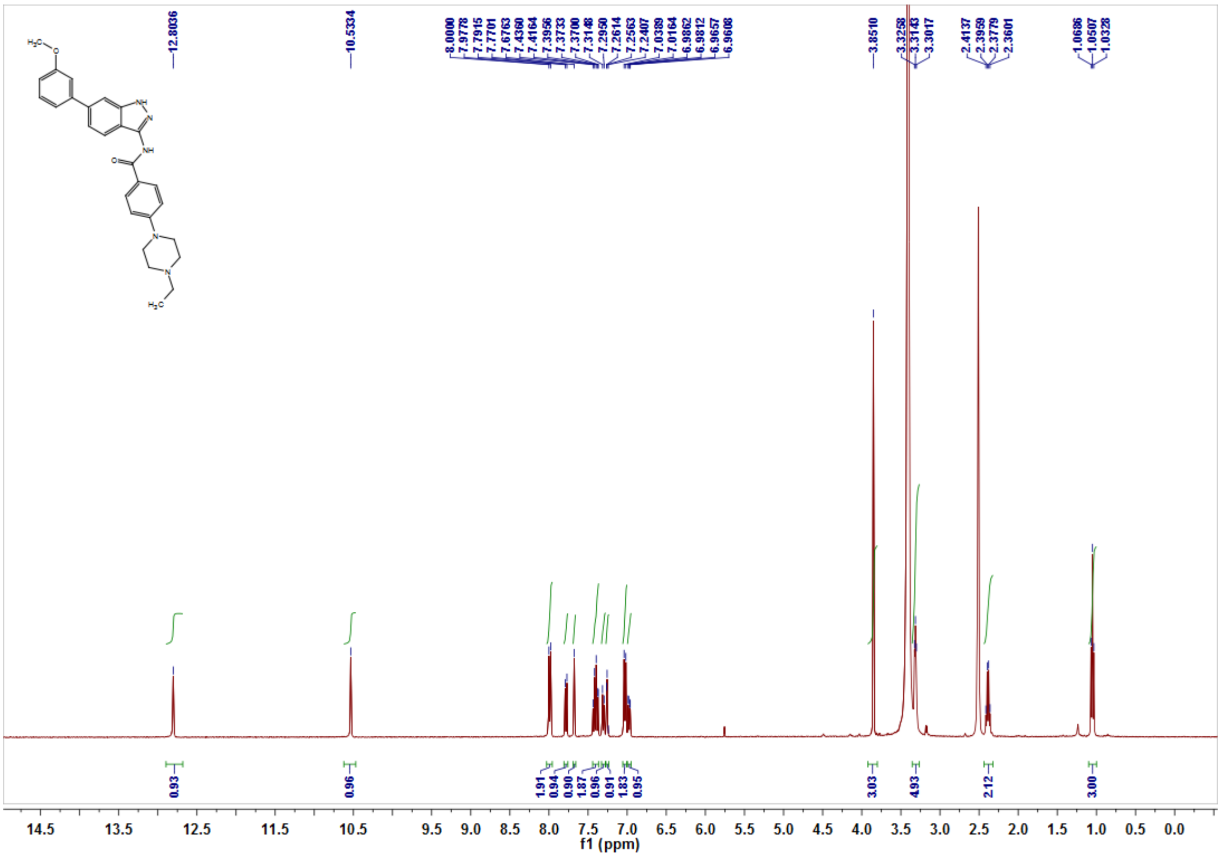

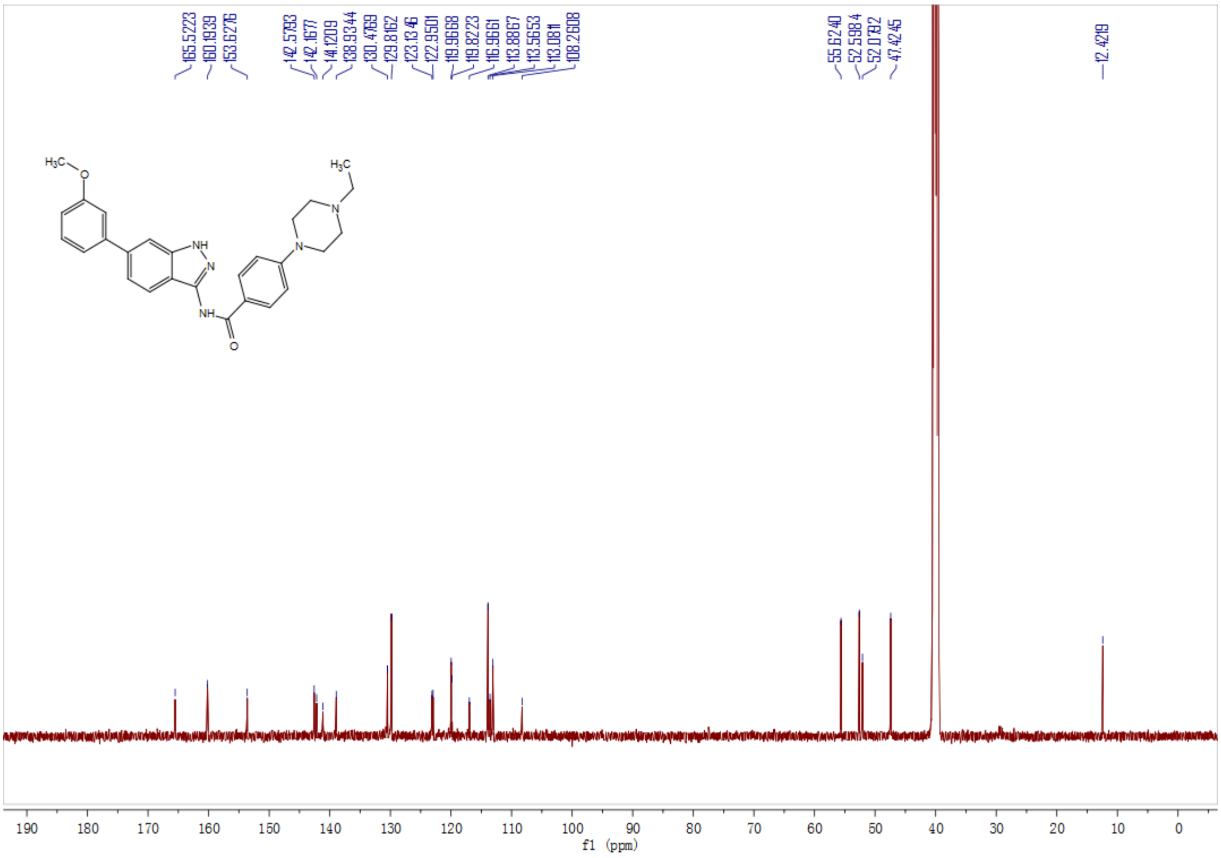

^{1}H NMR and ^{13}C NMR of compound **9e**

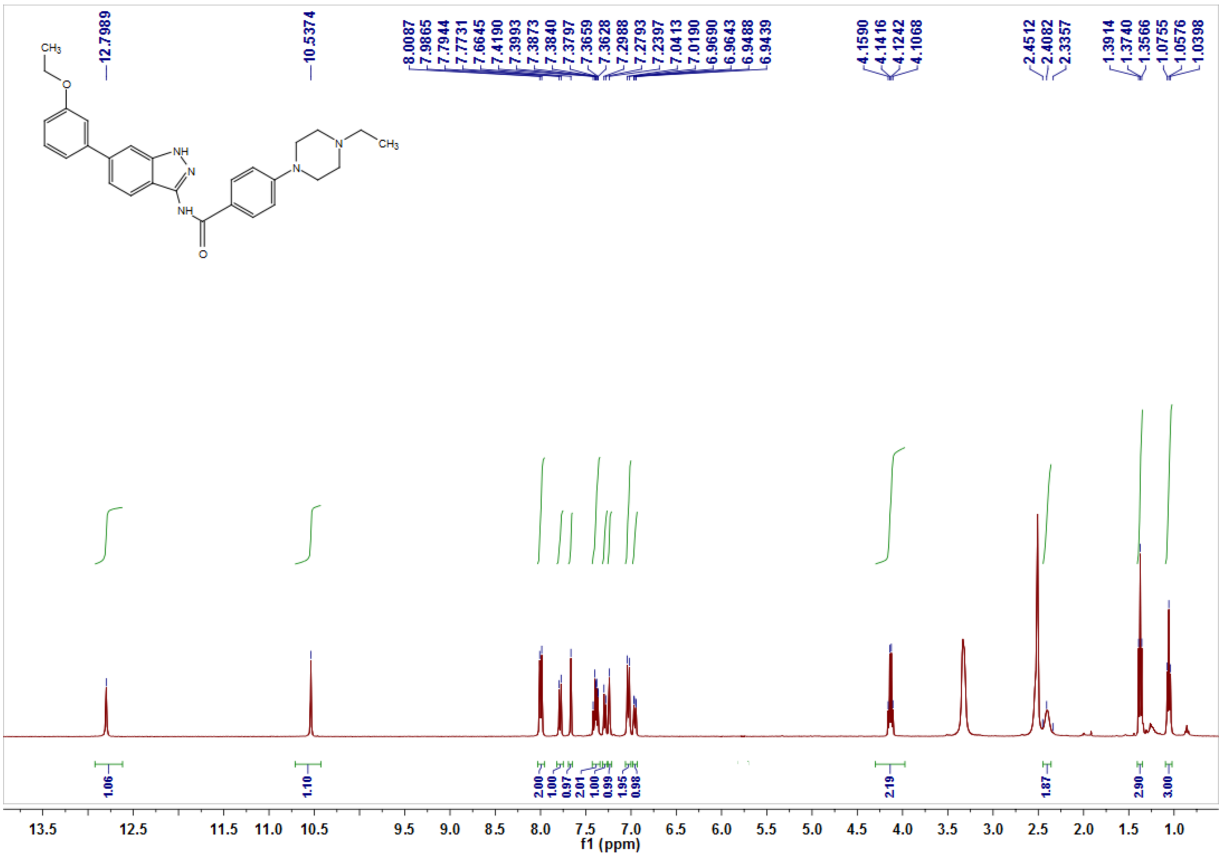

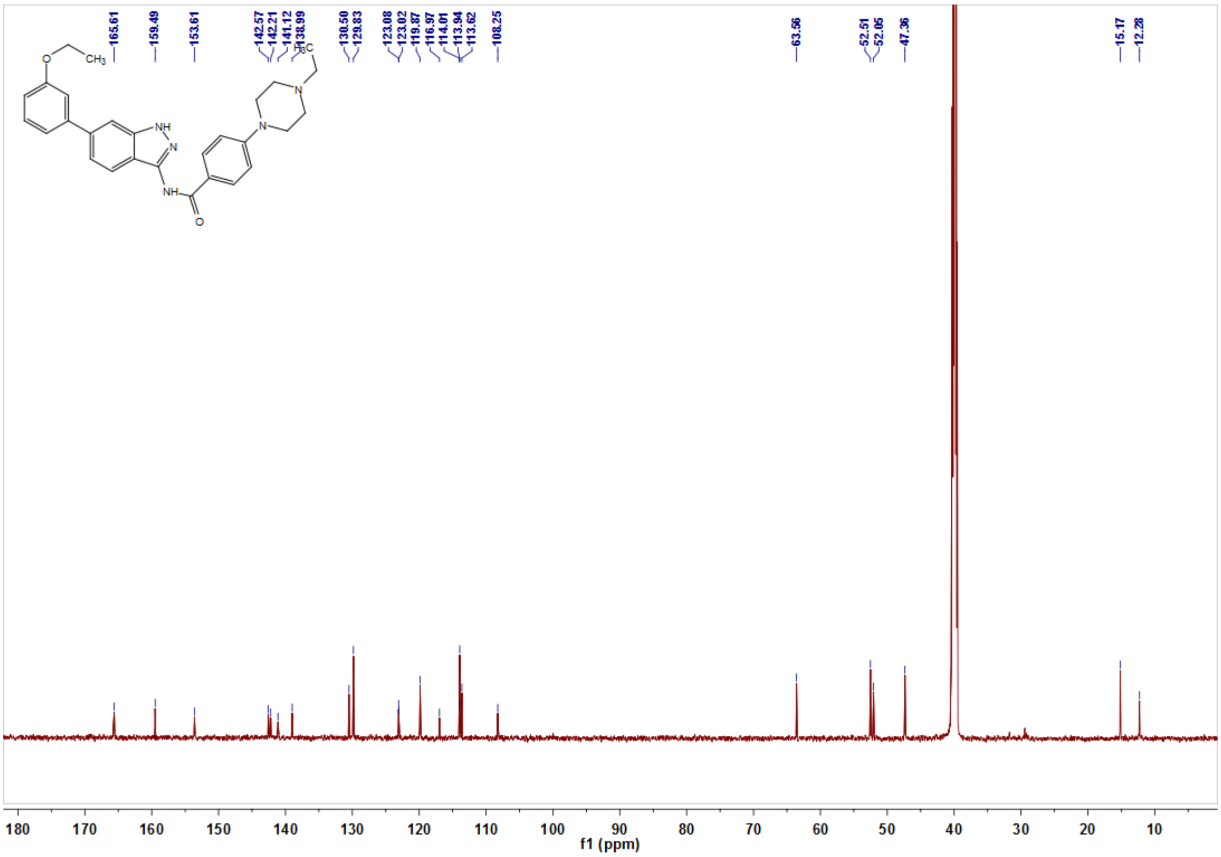

^{1}H NMR and ^{13}C NMR of compound **9f**

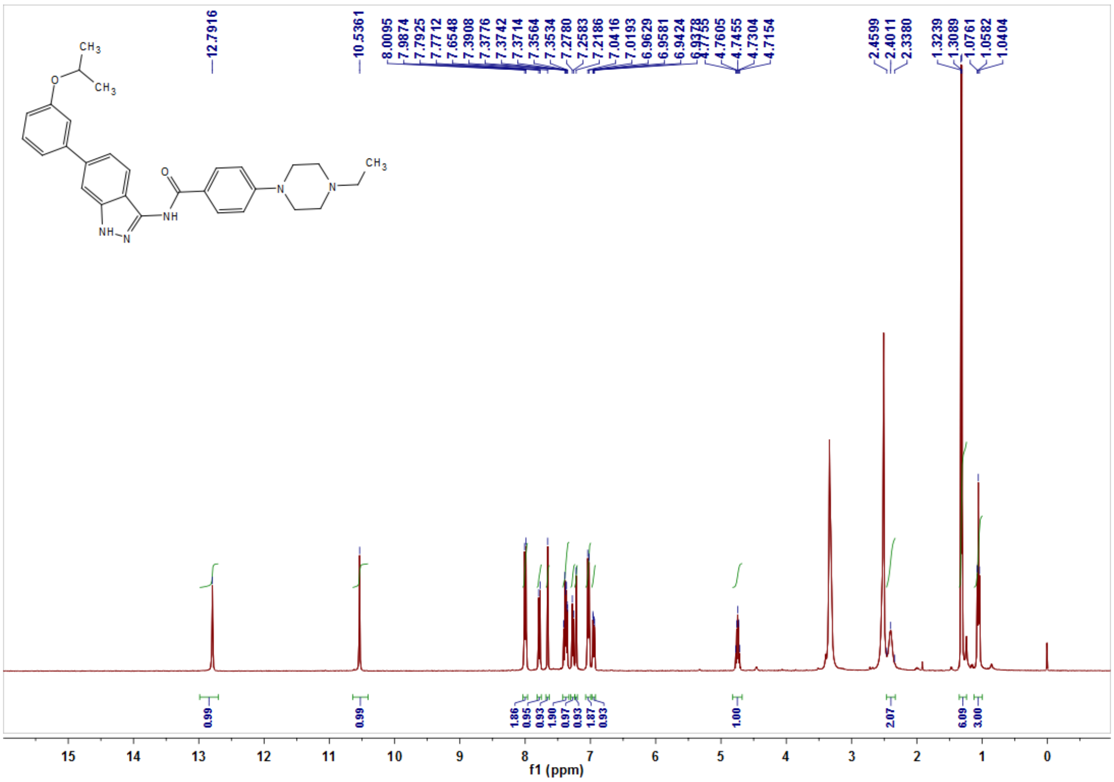

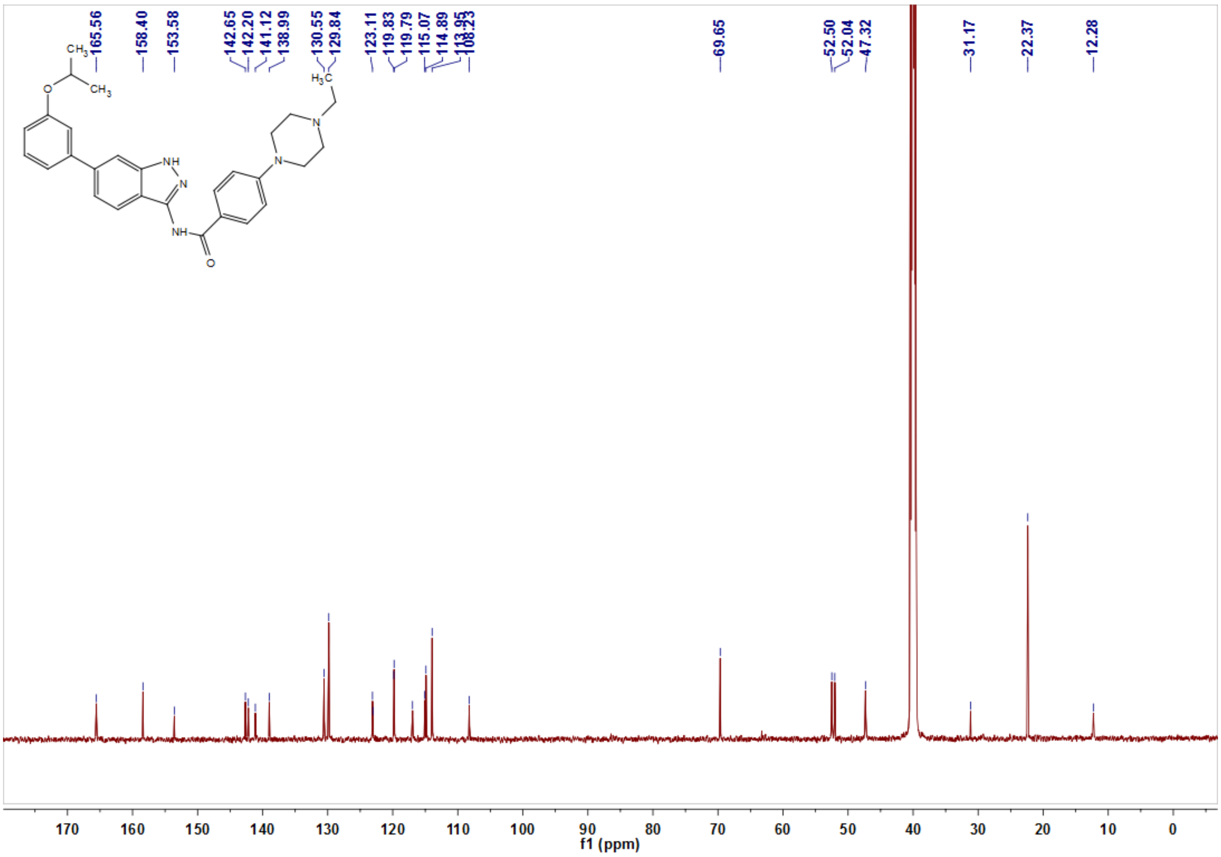

^{1}H NMR and ^{13}C NMR of compound **9g**

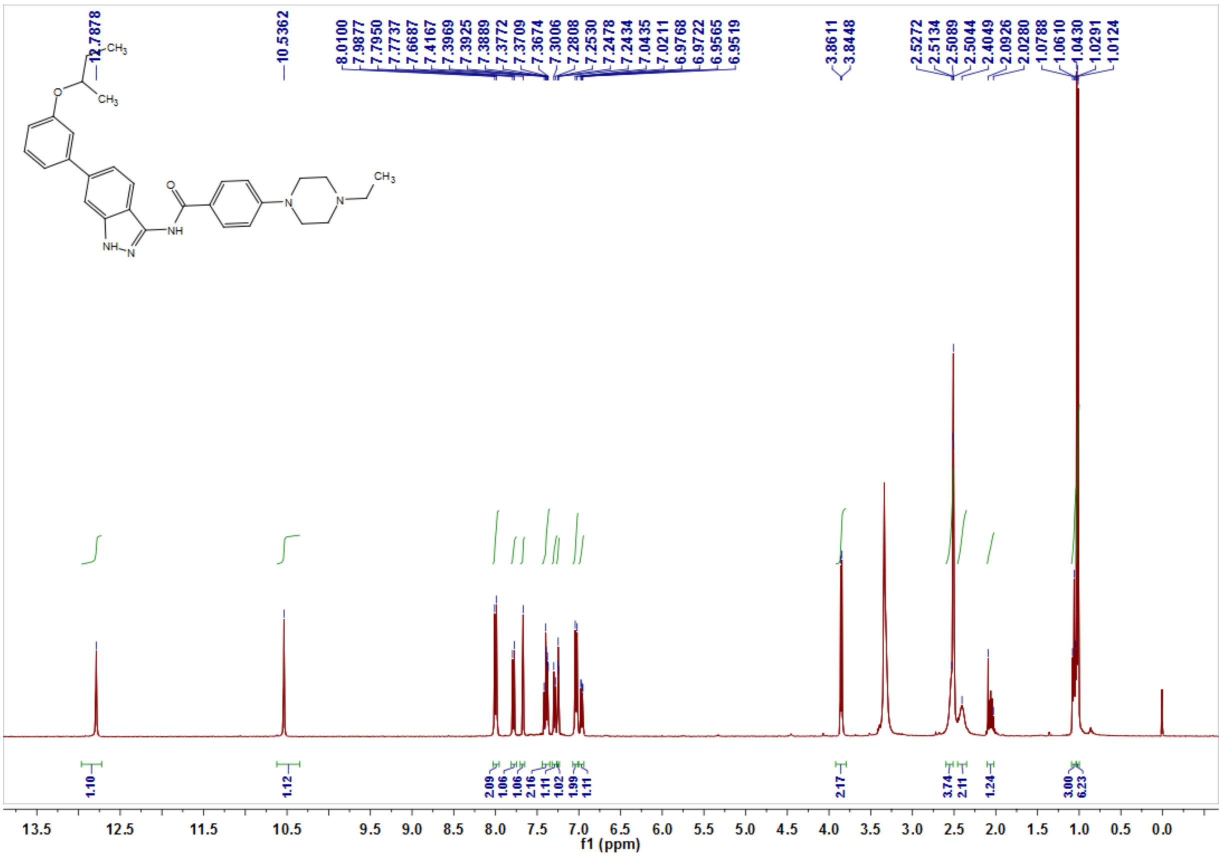

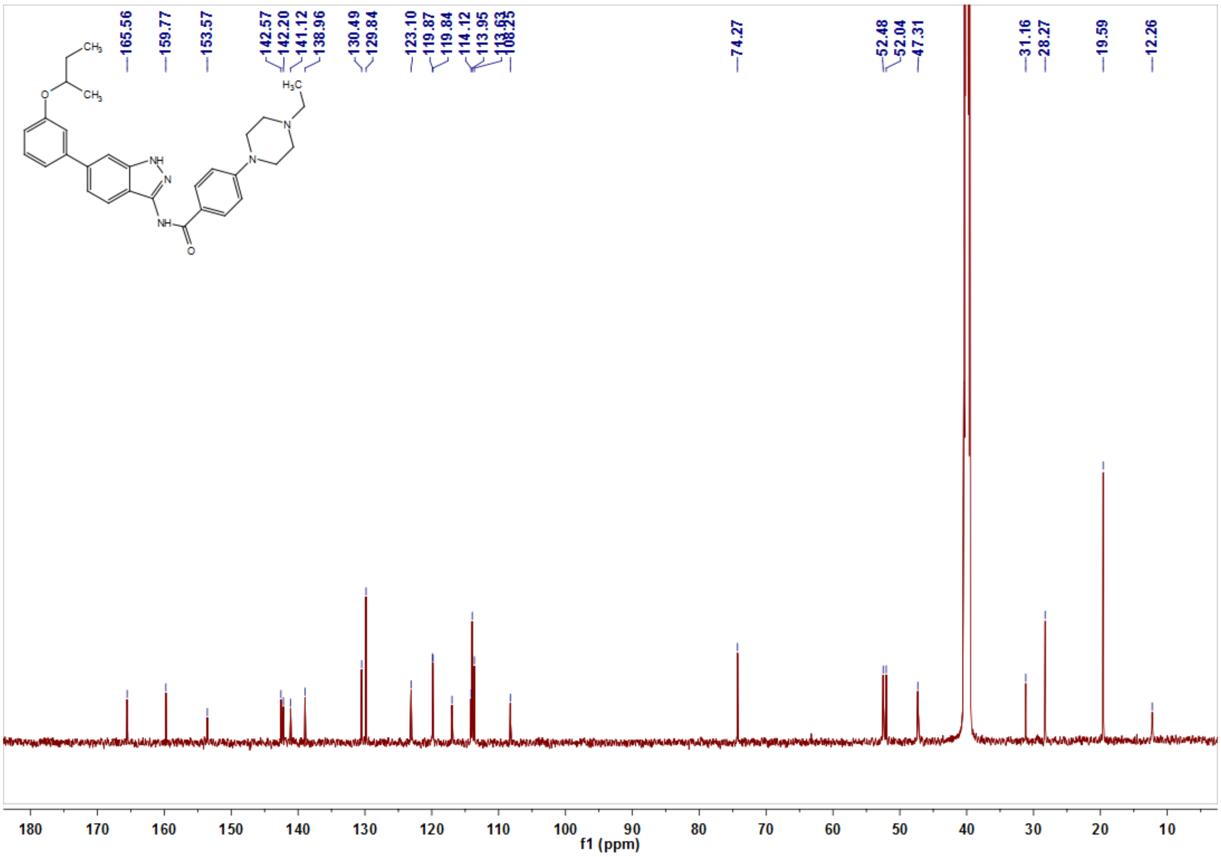

^{1}H NMR and ^{13}C NMR of compound **9h**

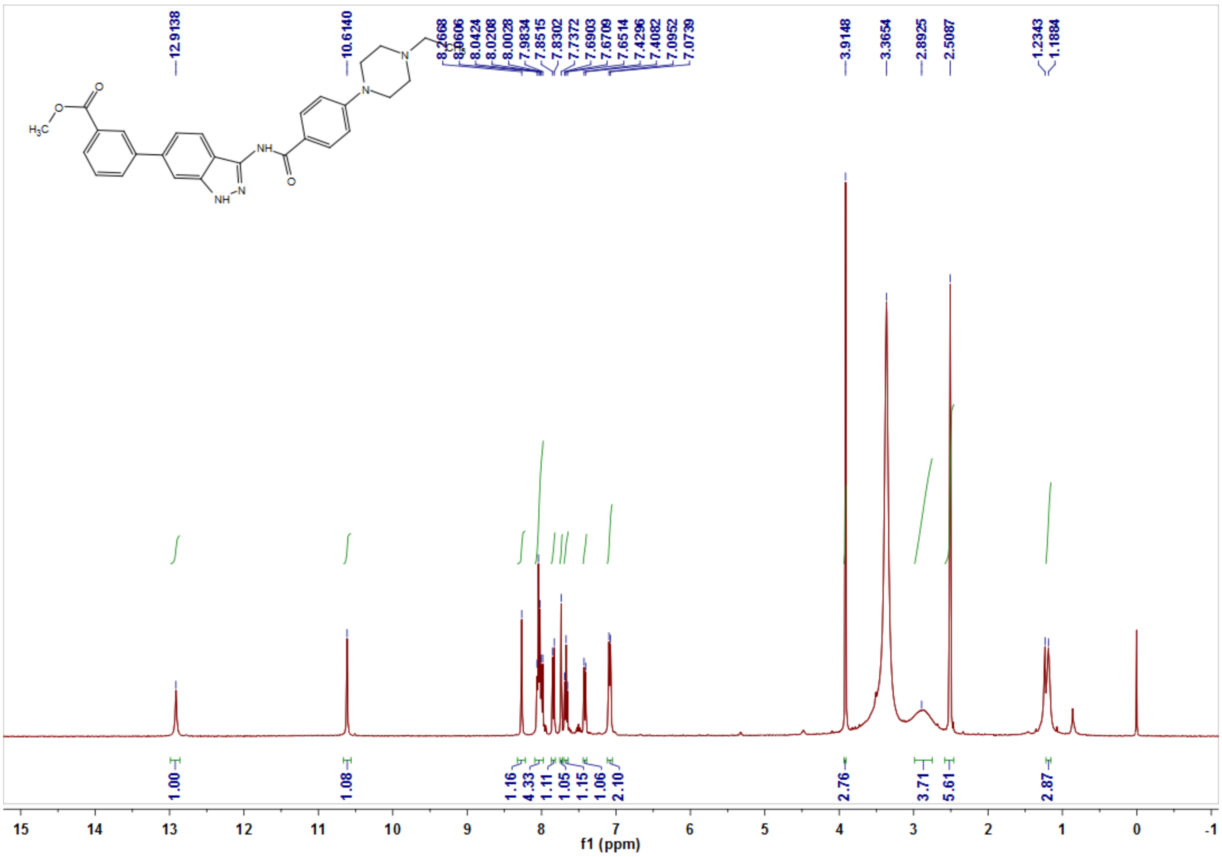

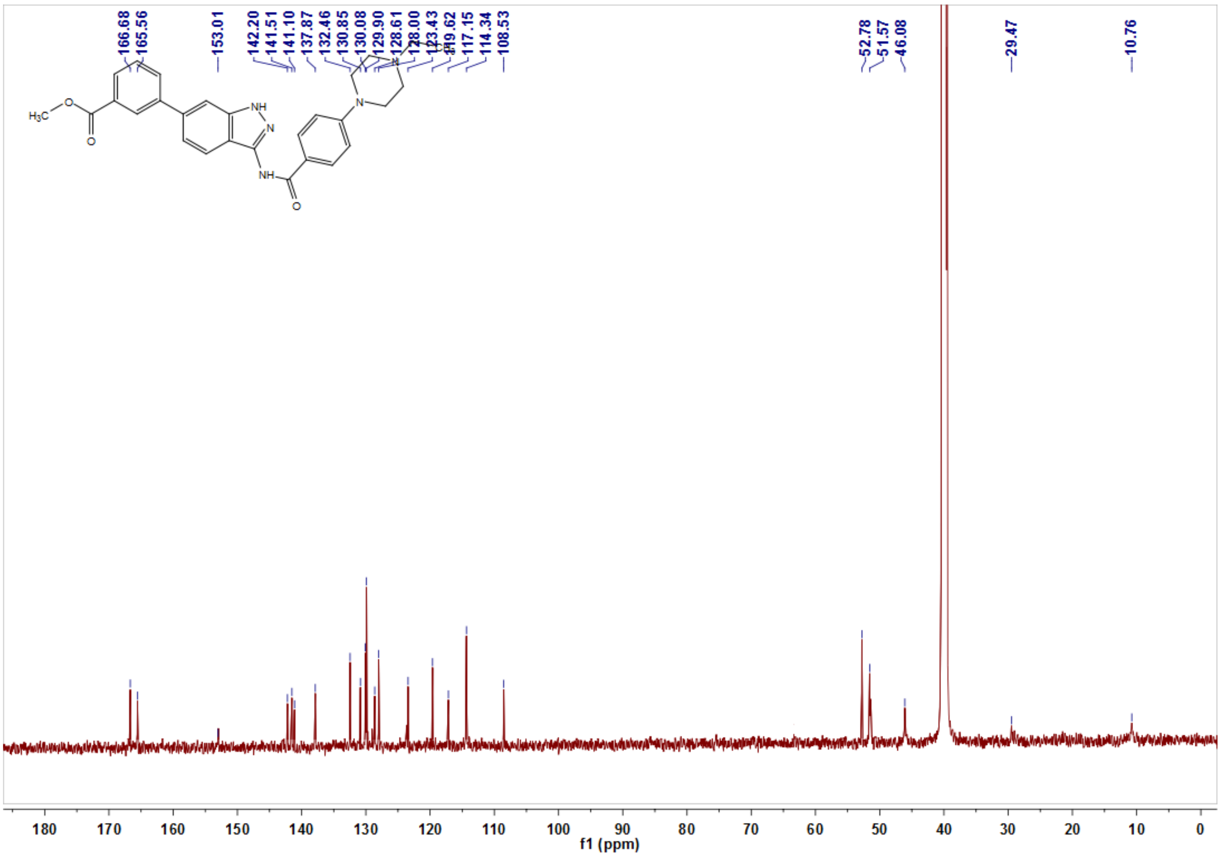

^{1}H NMR and ^{13}C NMR of compound **9i**

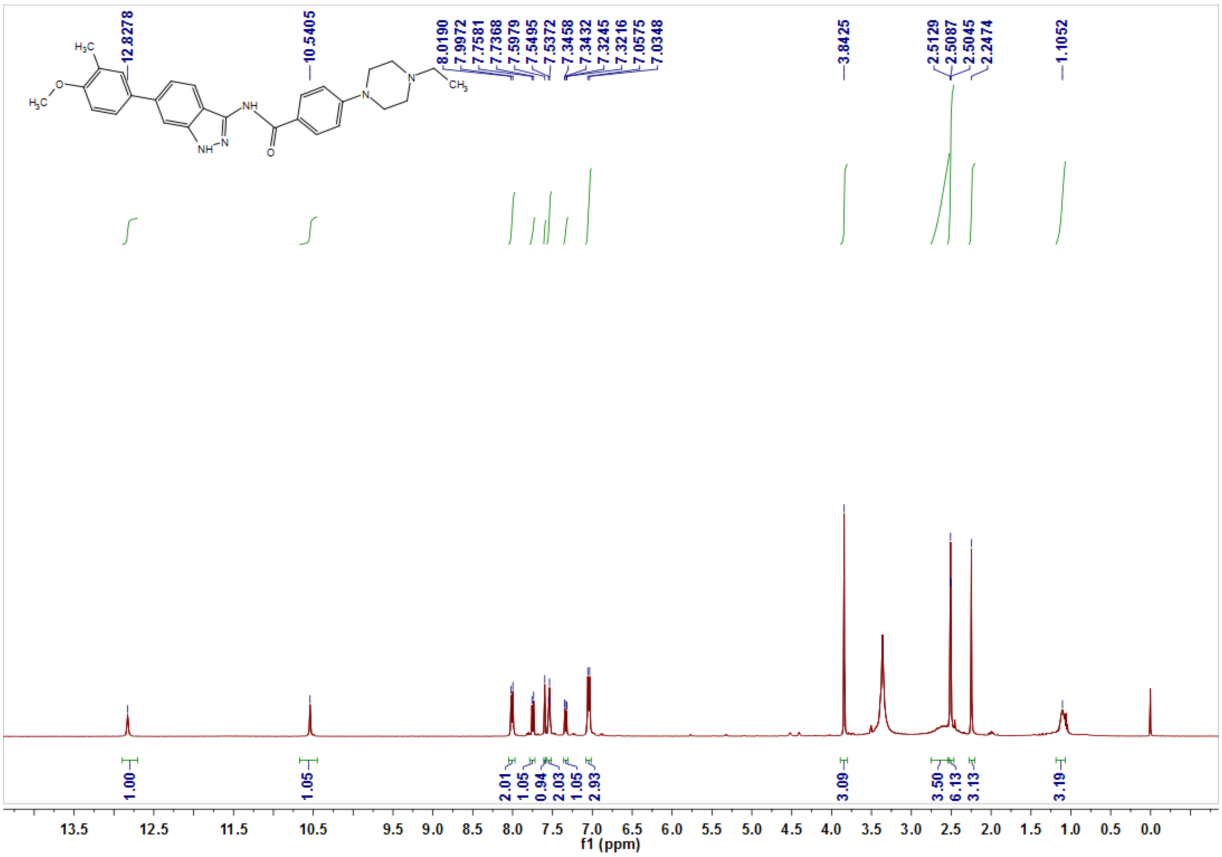

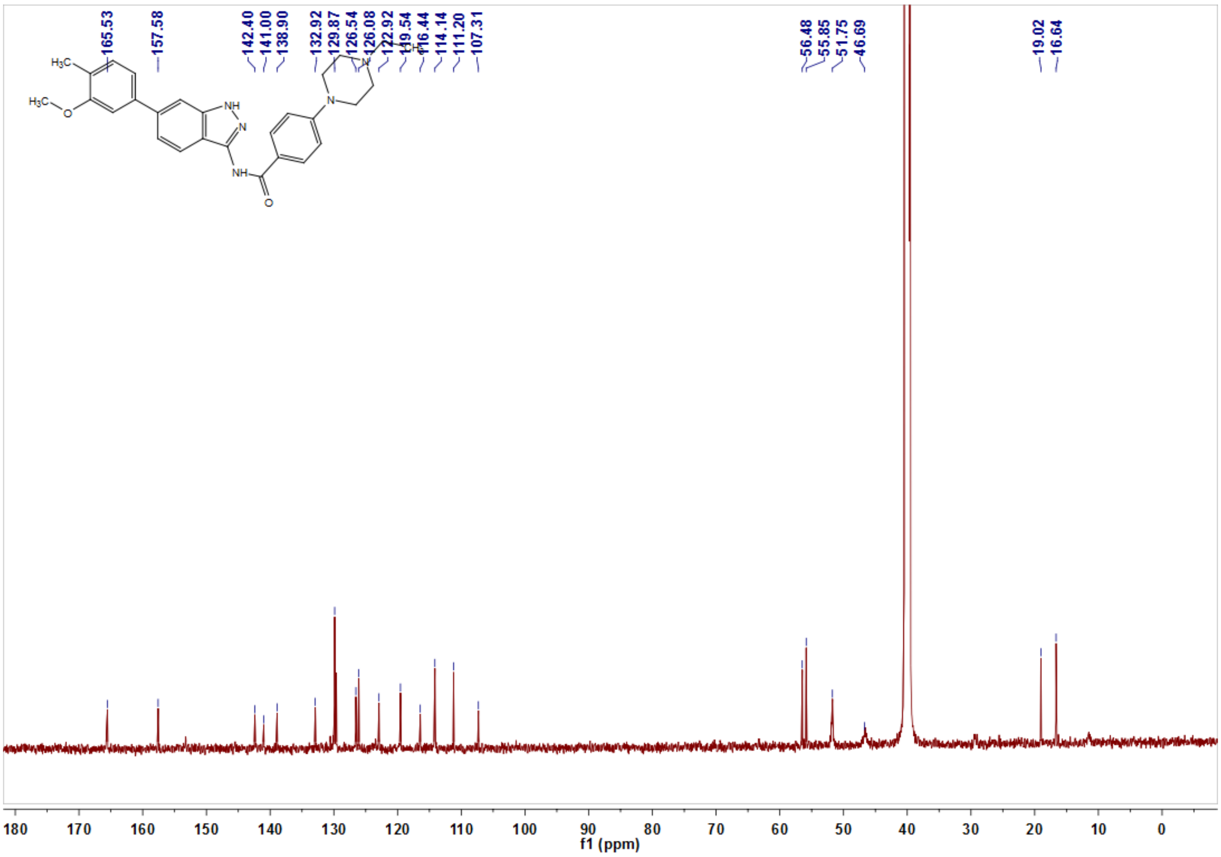

^{1}H NMR and ^{13}C NMR of compound **9j**

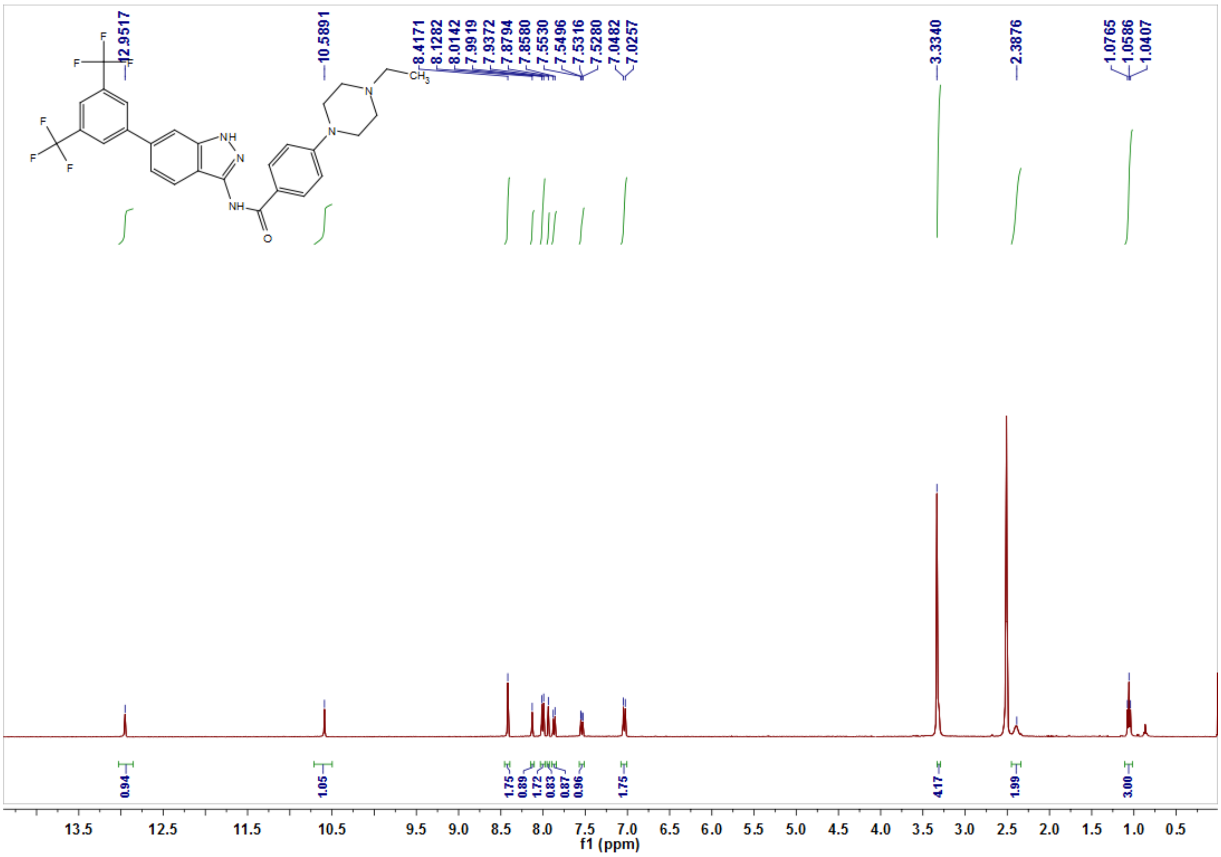

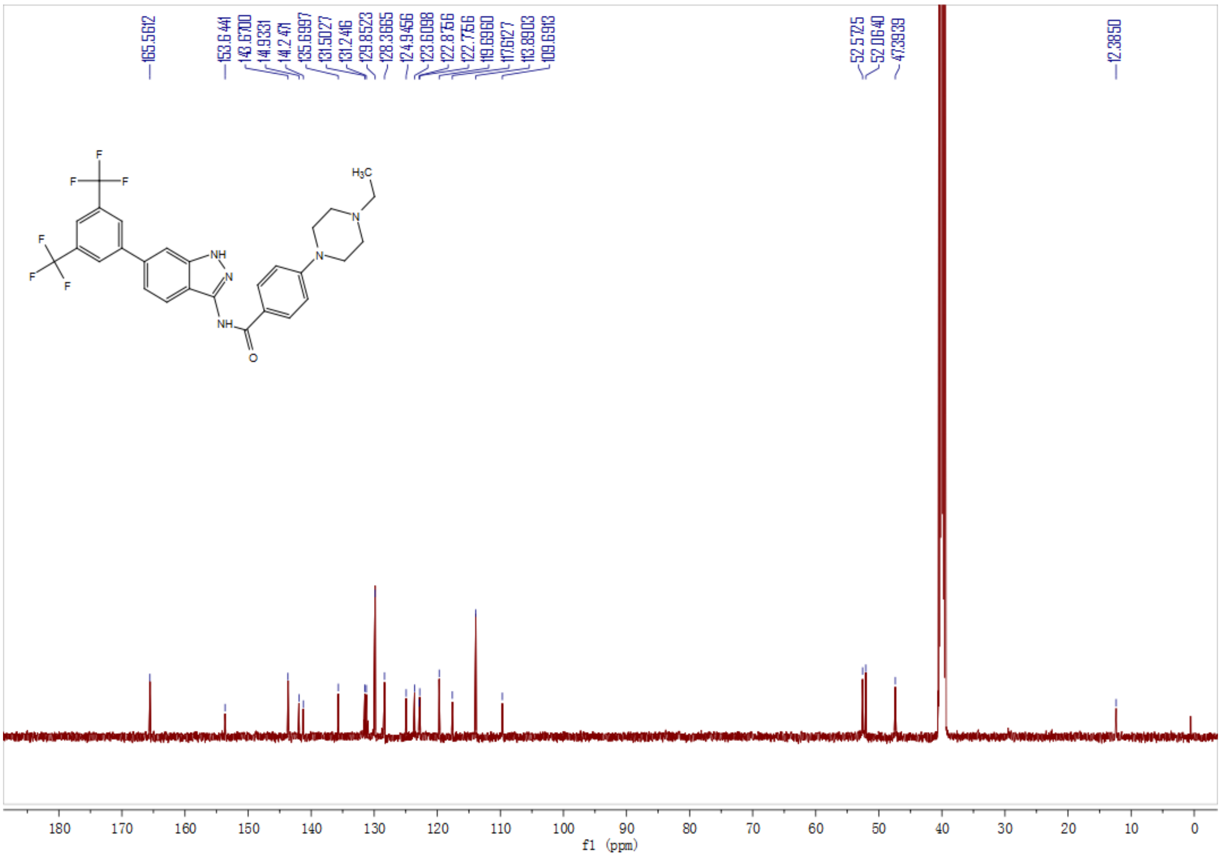

^{1}H NMR and ^{13}C NMR of compound **9k**

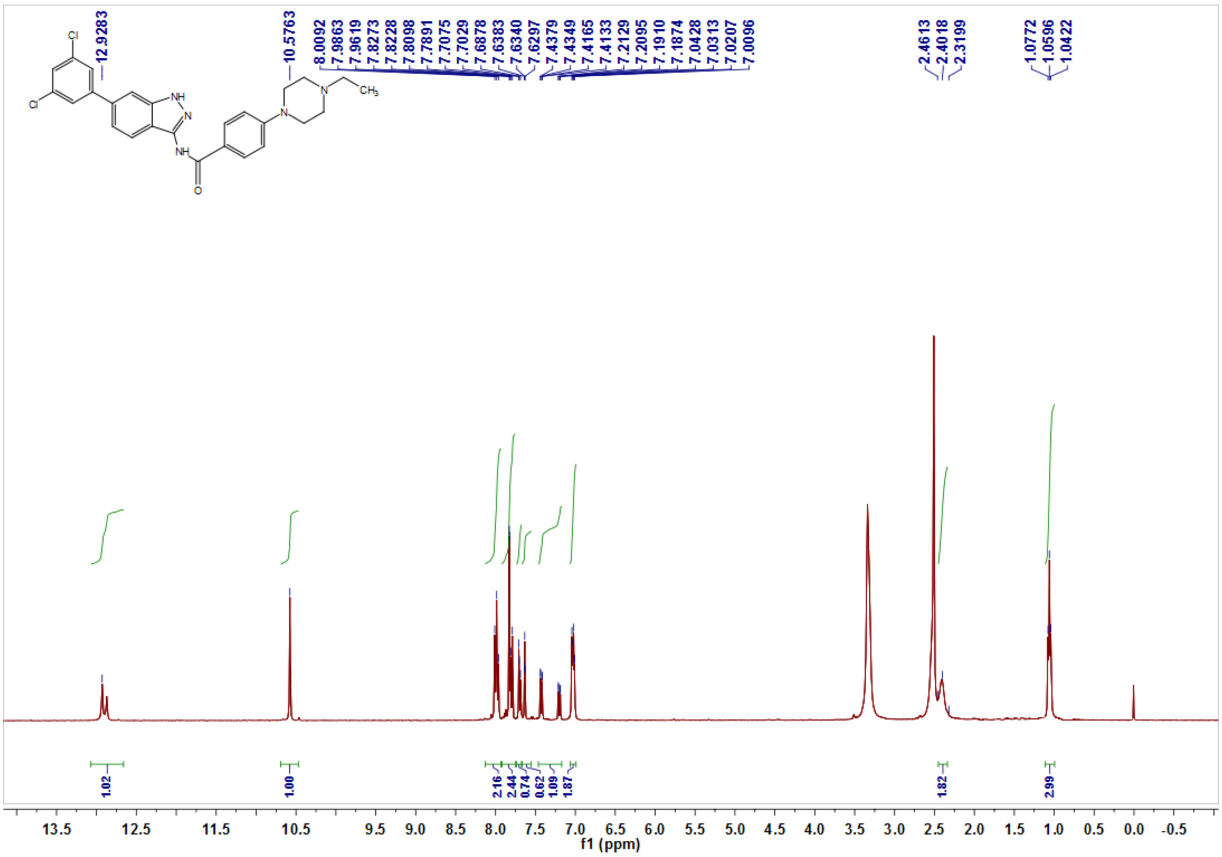

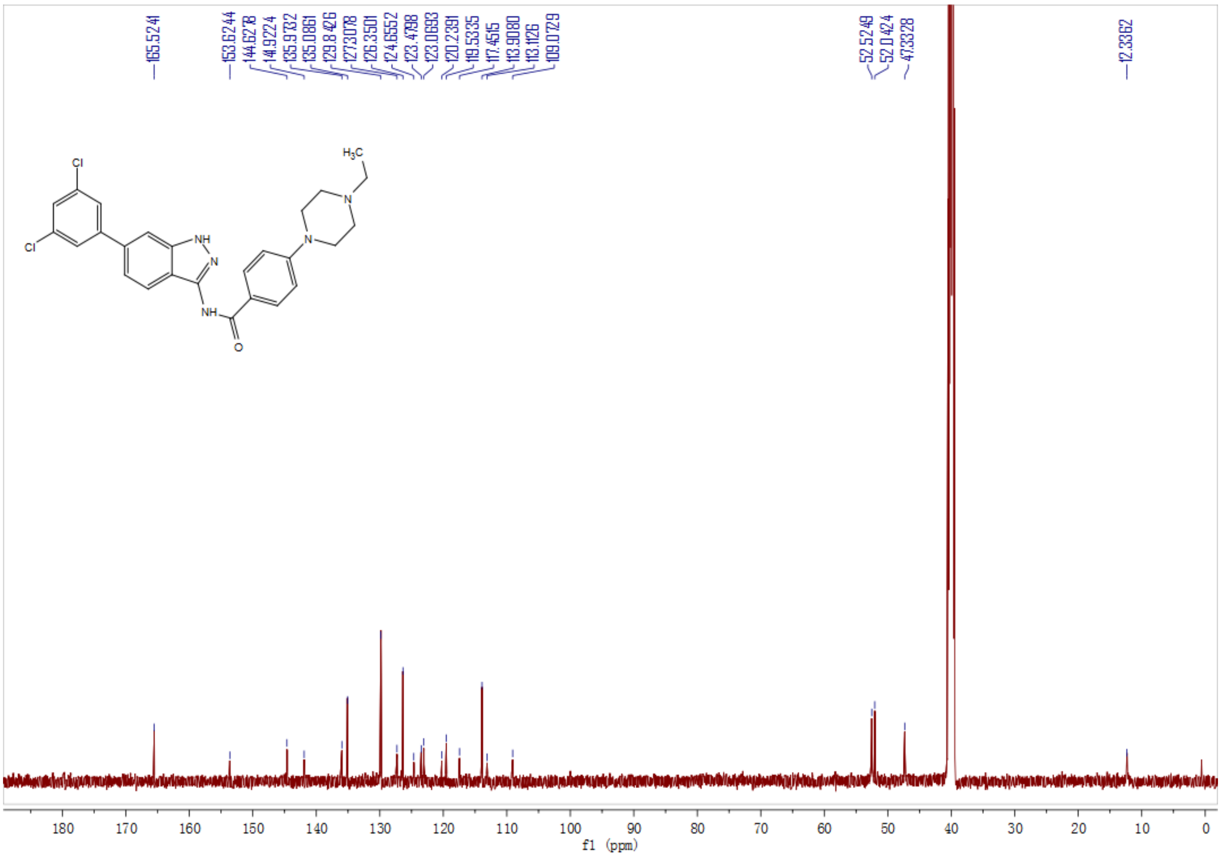

^{1}H NMR and ^{13}C NMR of compound **9l**

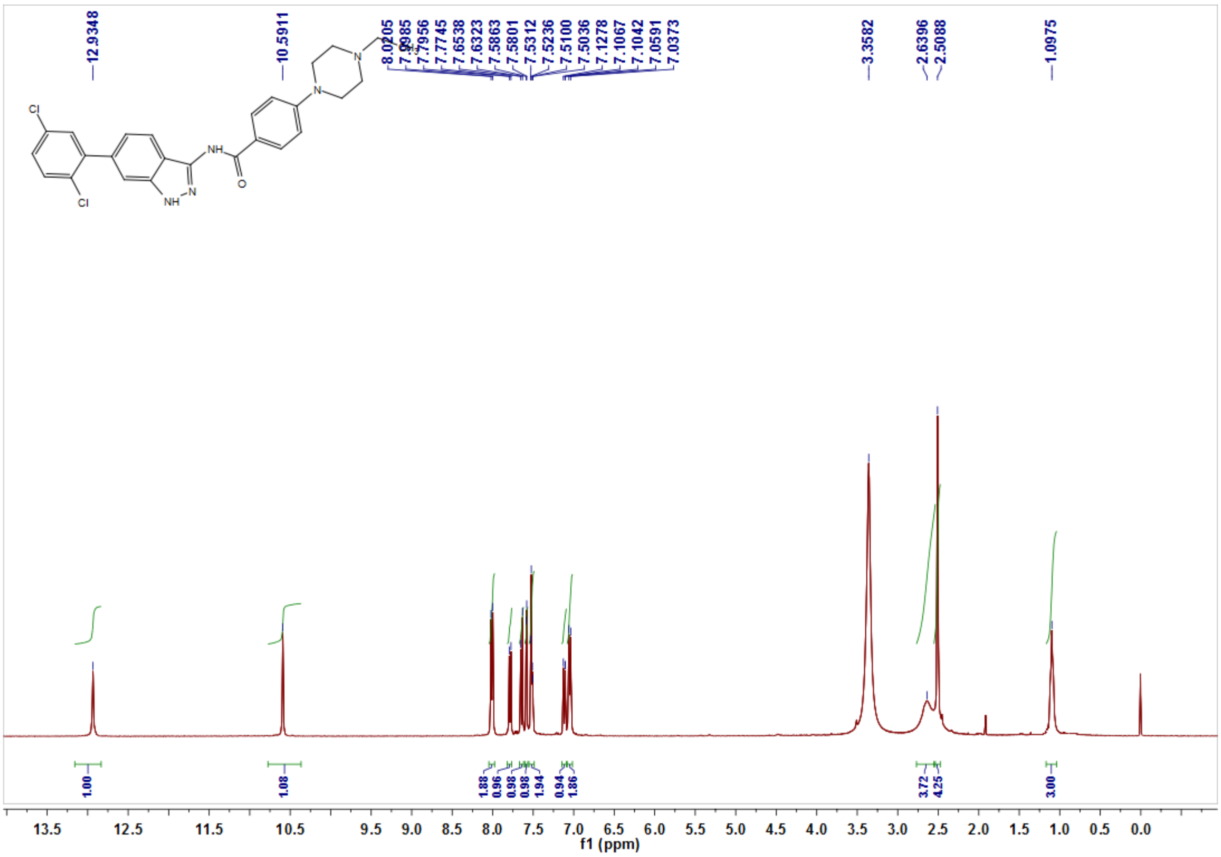

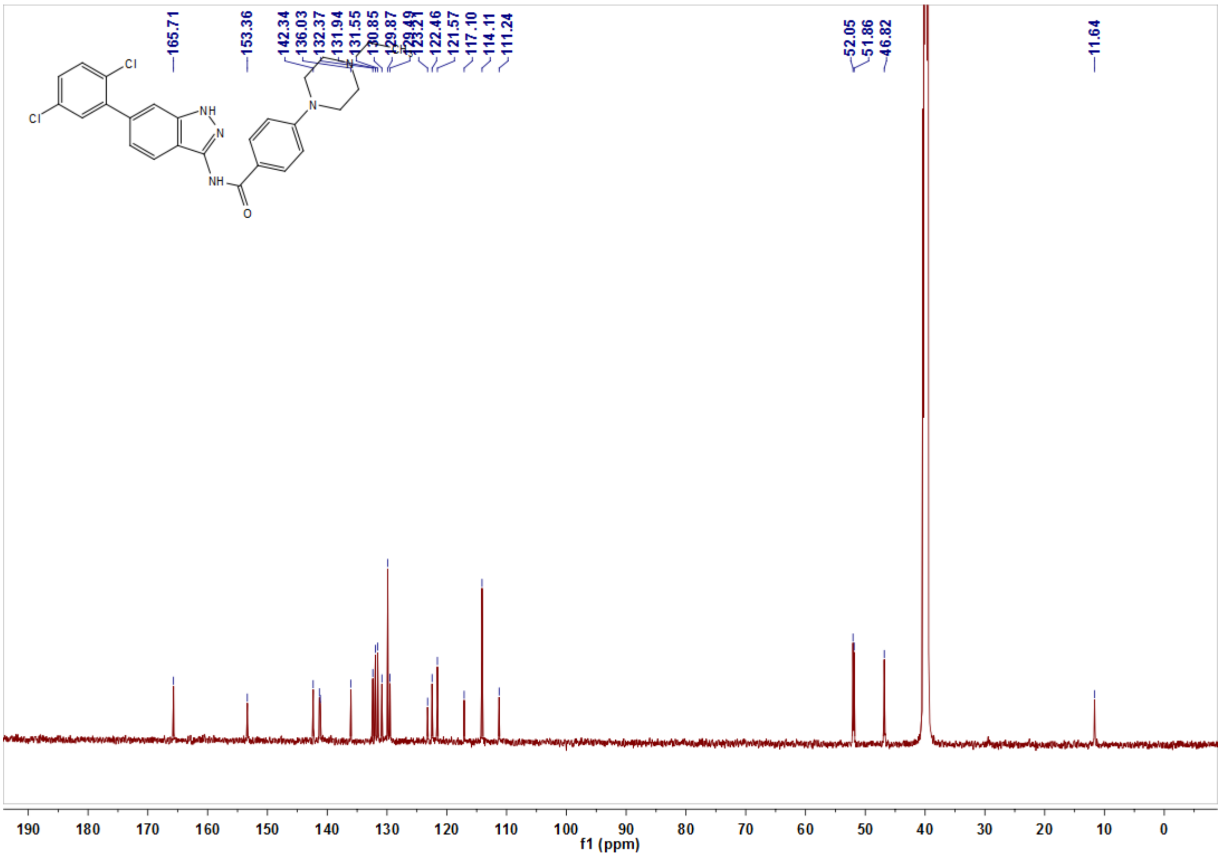

^{1}H NMR and ^{13}C NMR of compound **9m**

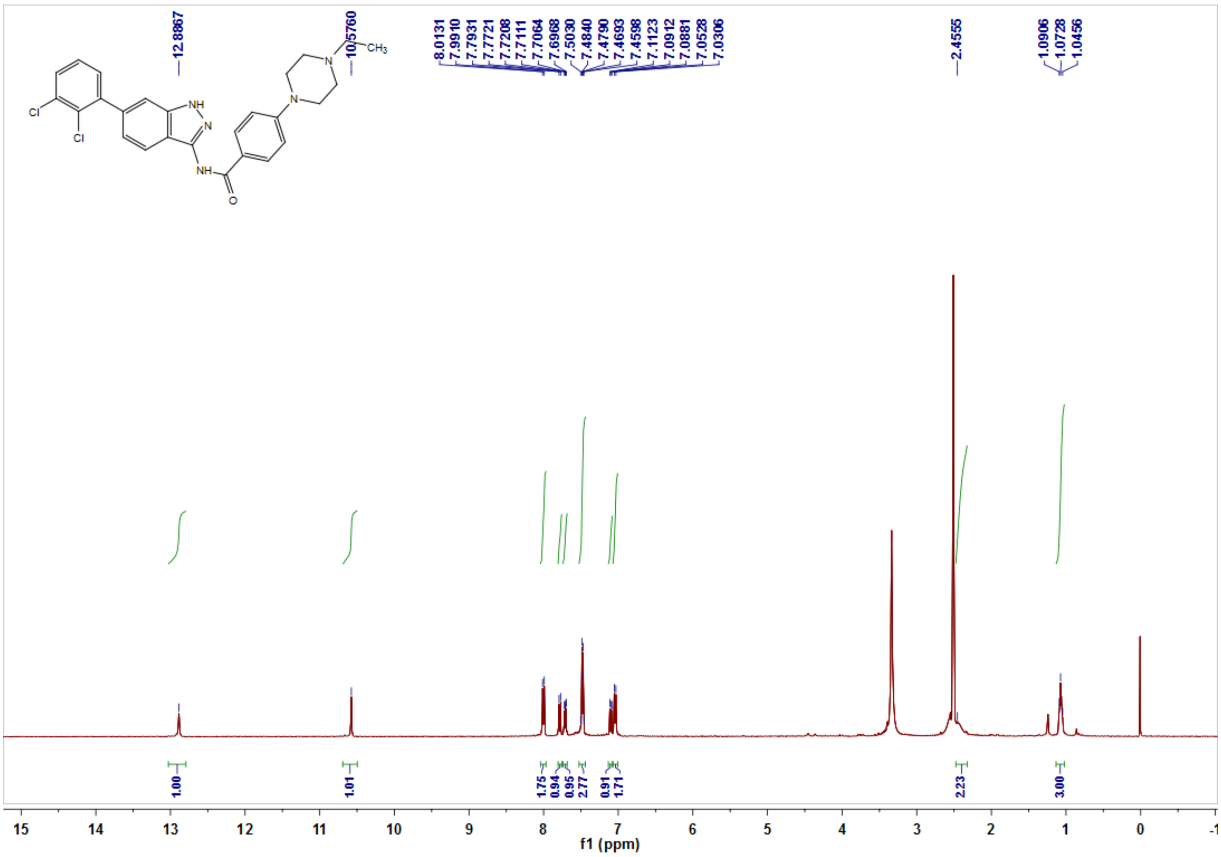

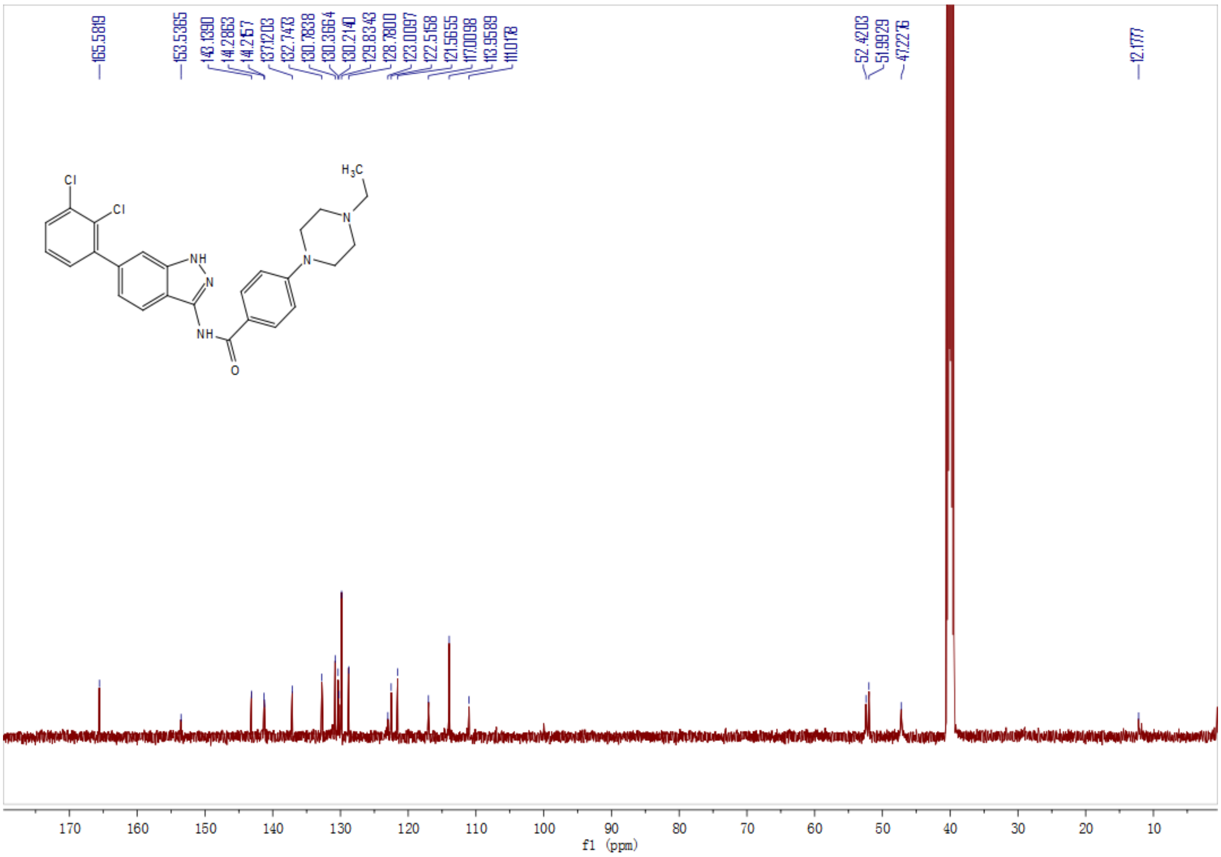

^{1}H NMR and ^{13}C NMR of compound **9n**

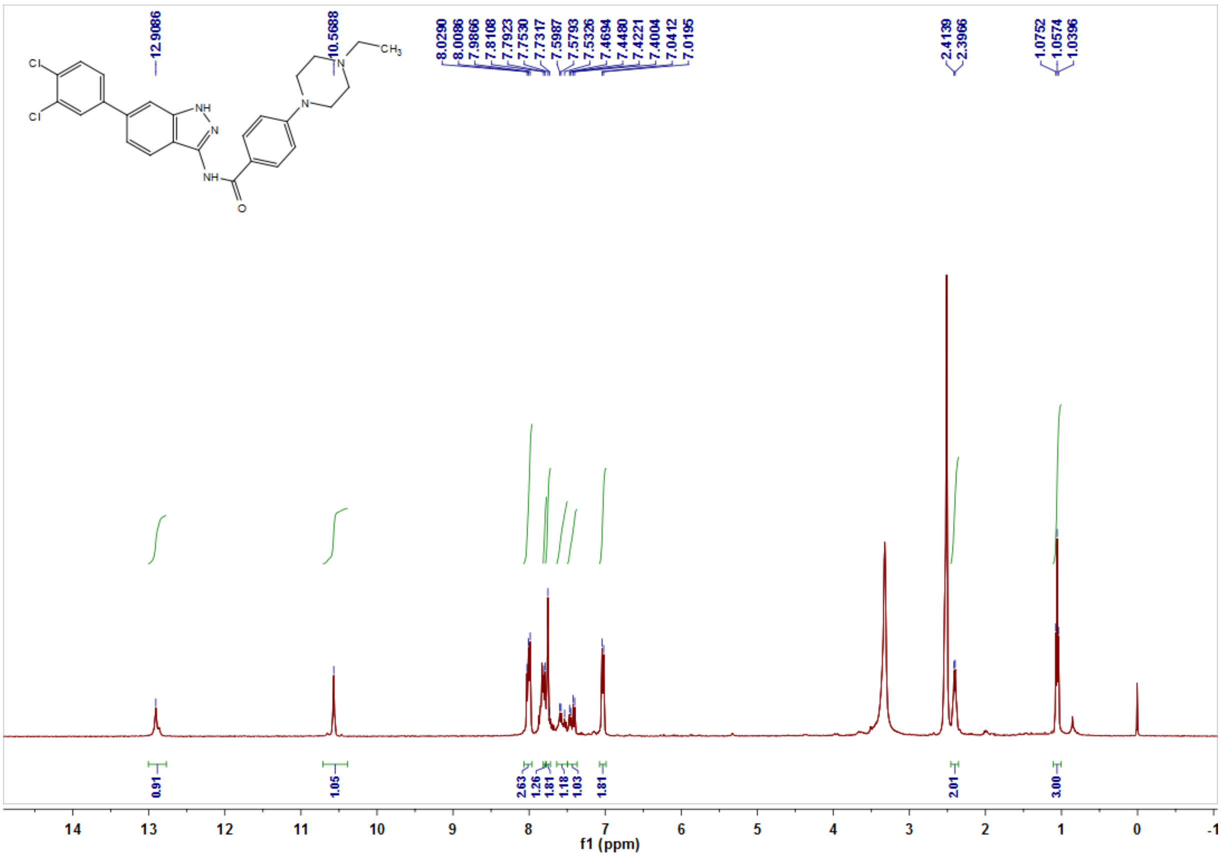

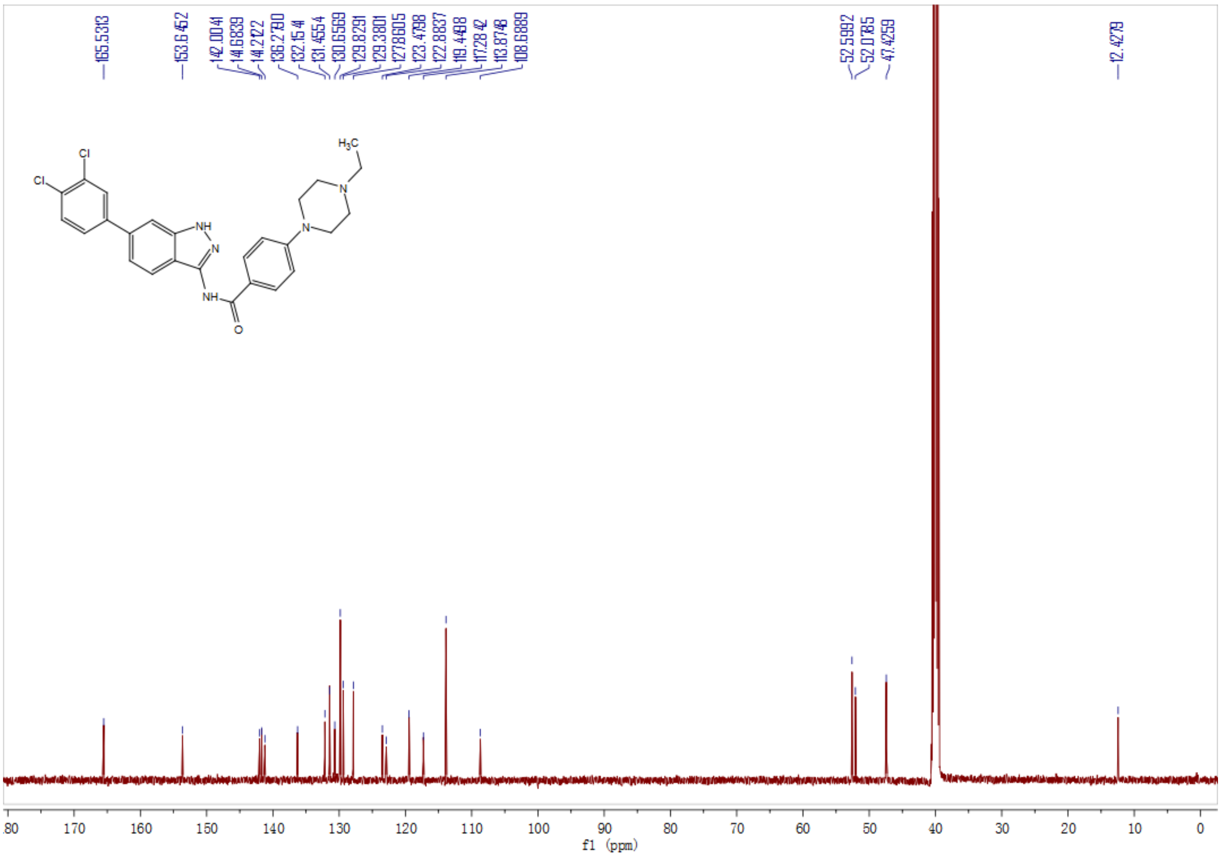

^{1}H NMR and ^{13}C NMR of compound **9o**

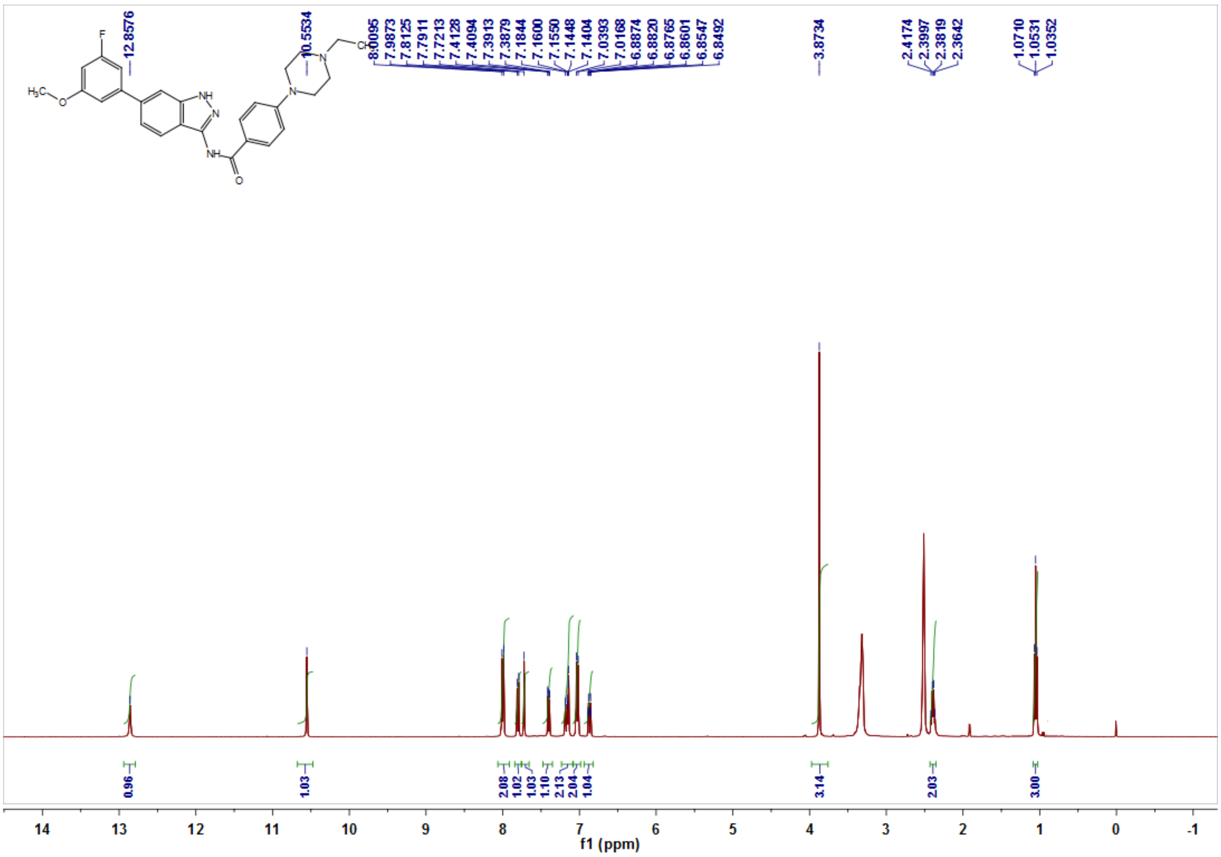

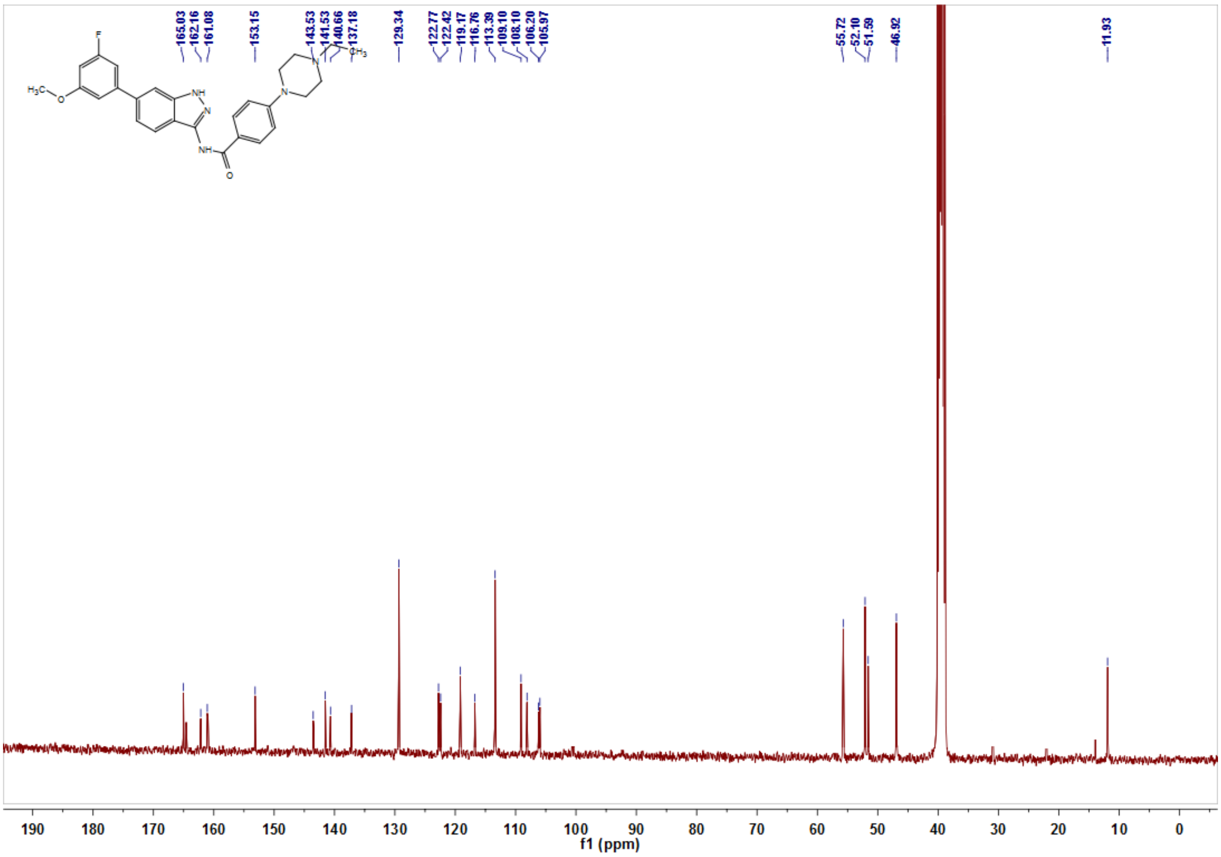

^{1}H NMR and ^{13}C NMR of compound **9p**

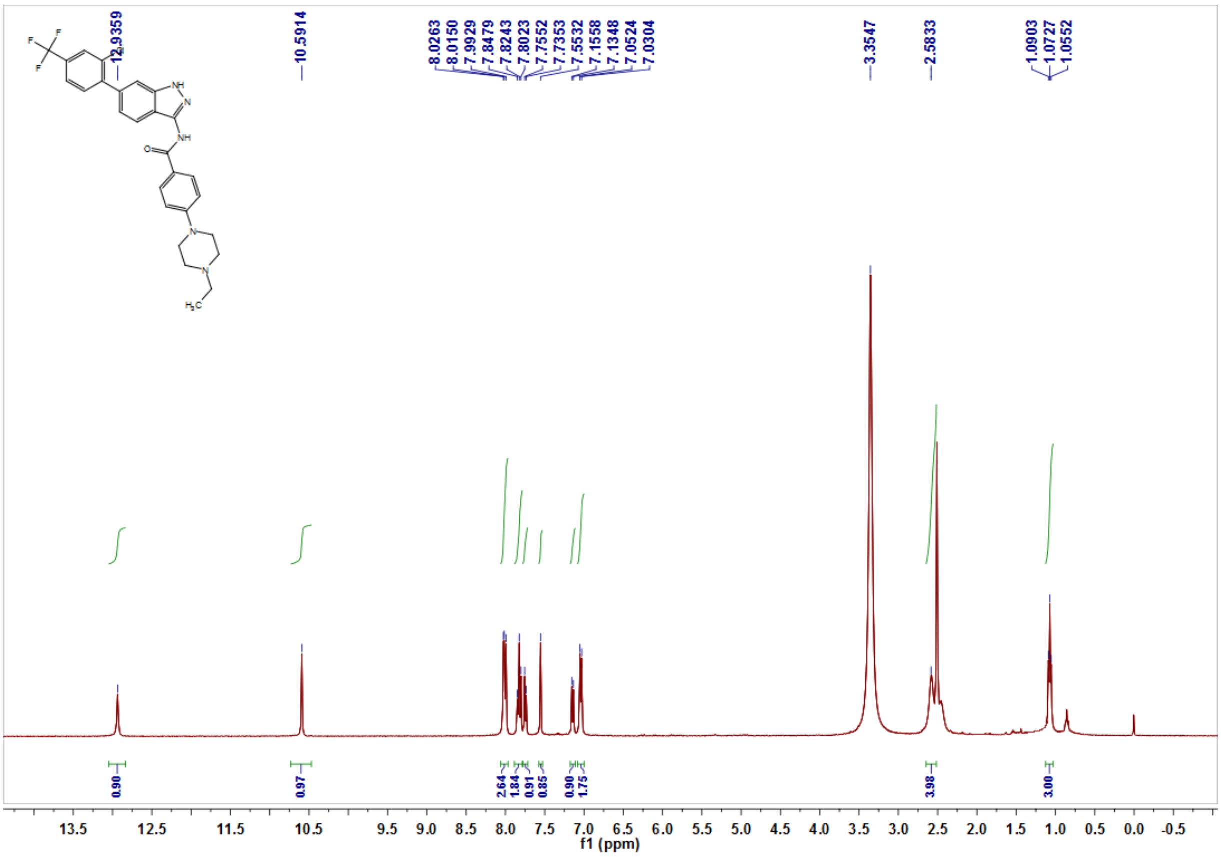

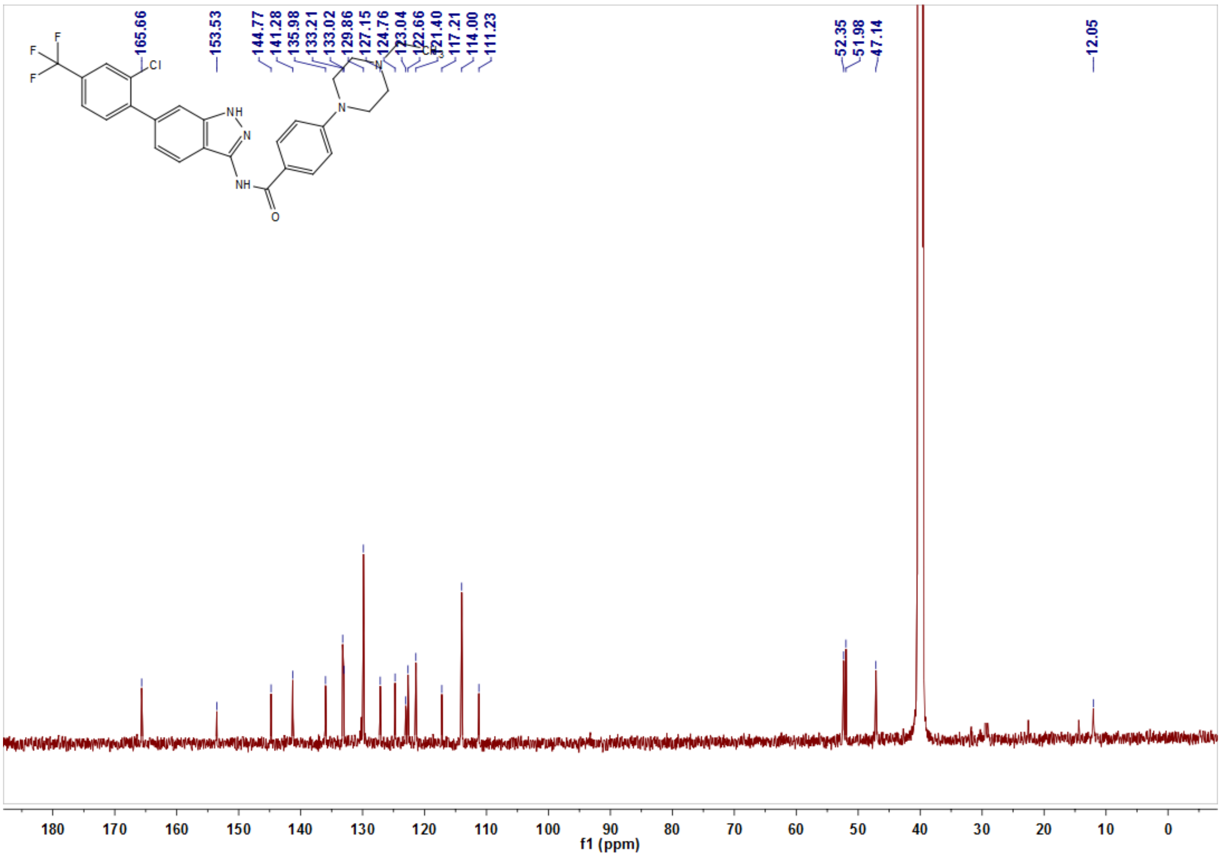

^{1}H NMR and ^{13}C NMR of compound **9q**

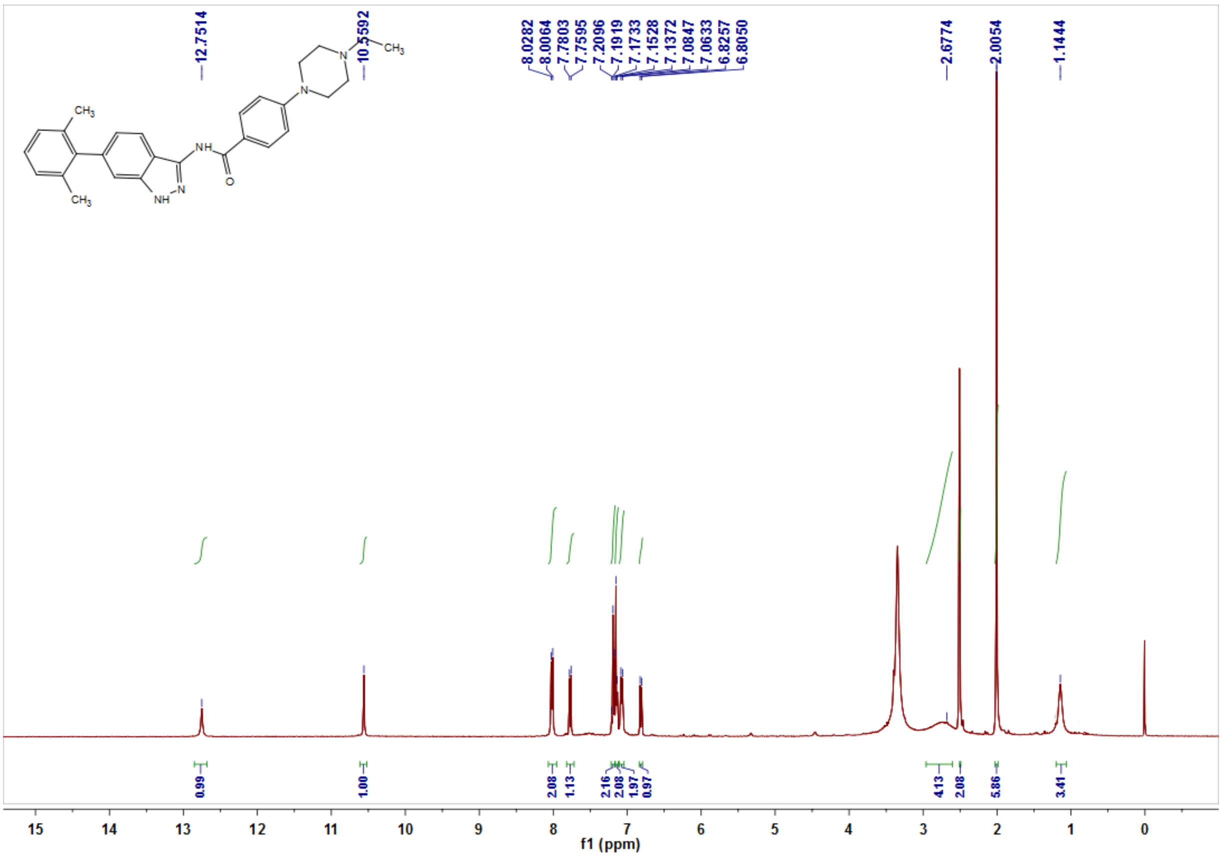

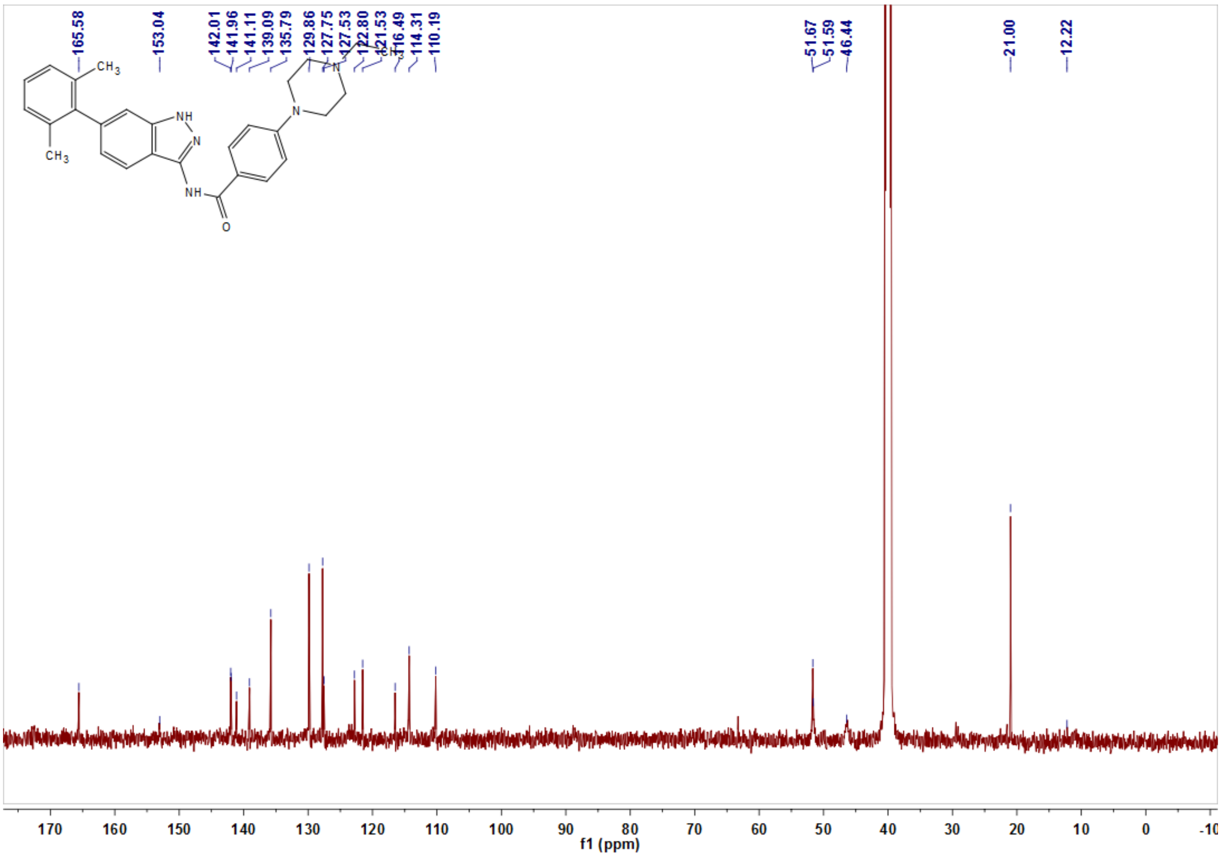

^{1}H NMR and ^{13}C NMR of compound **9r**

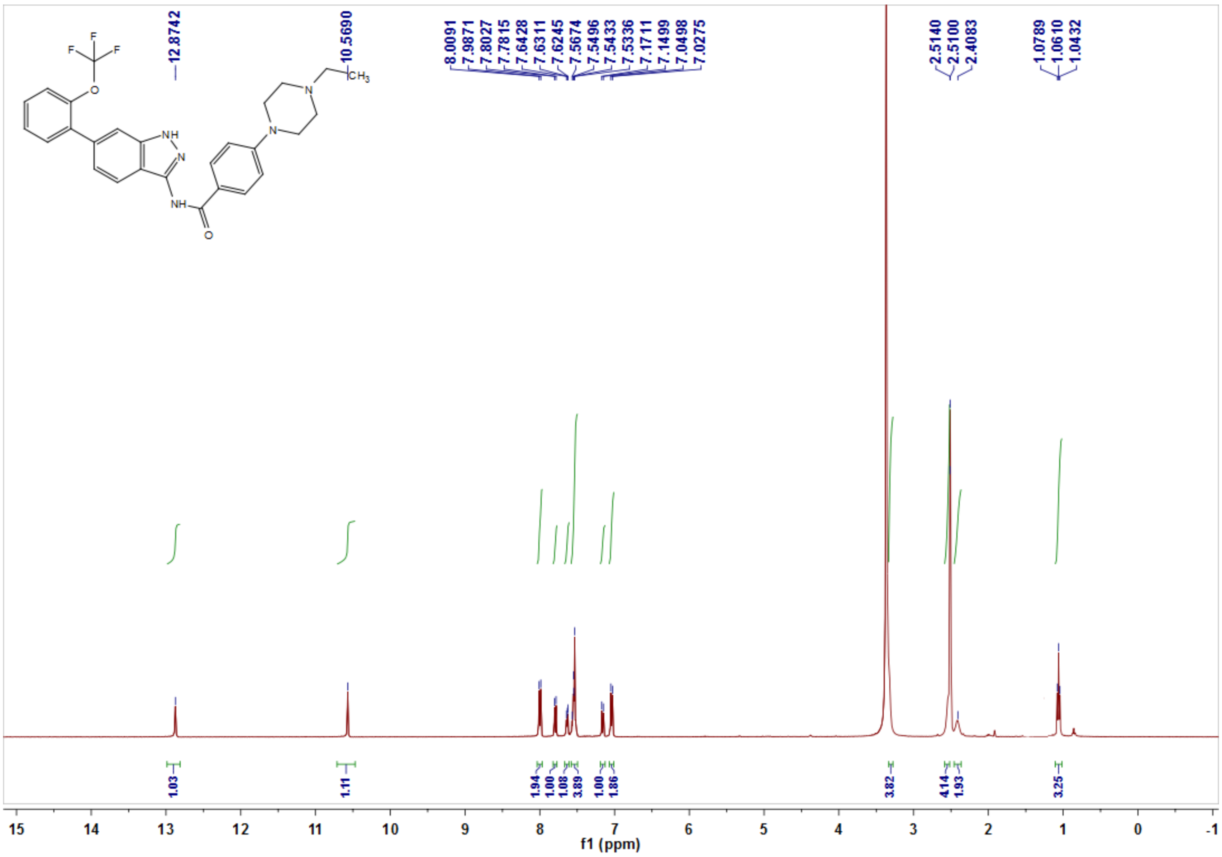

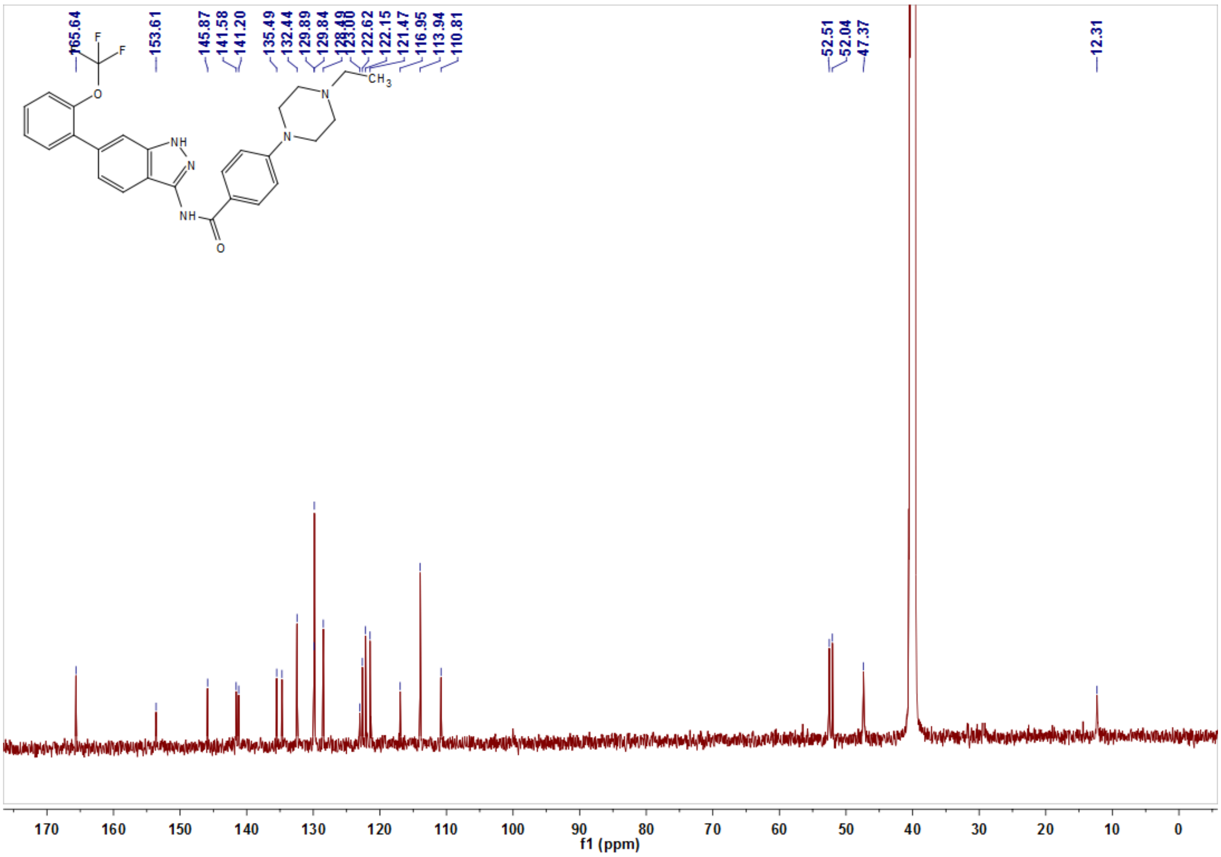

^{1}H NMR and ^{13}C NMR of compound **9s**

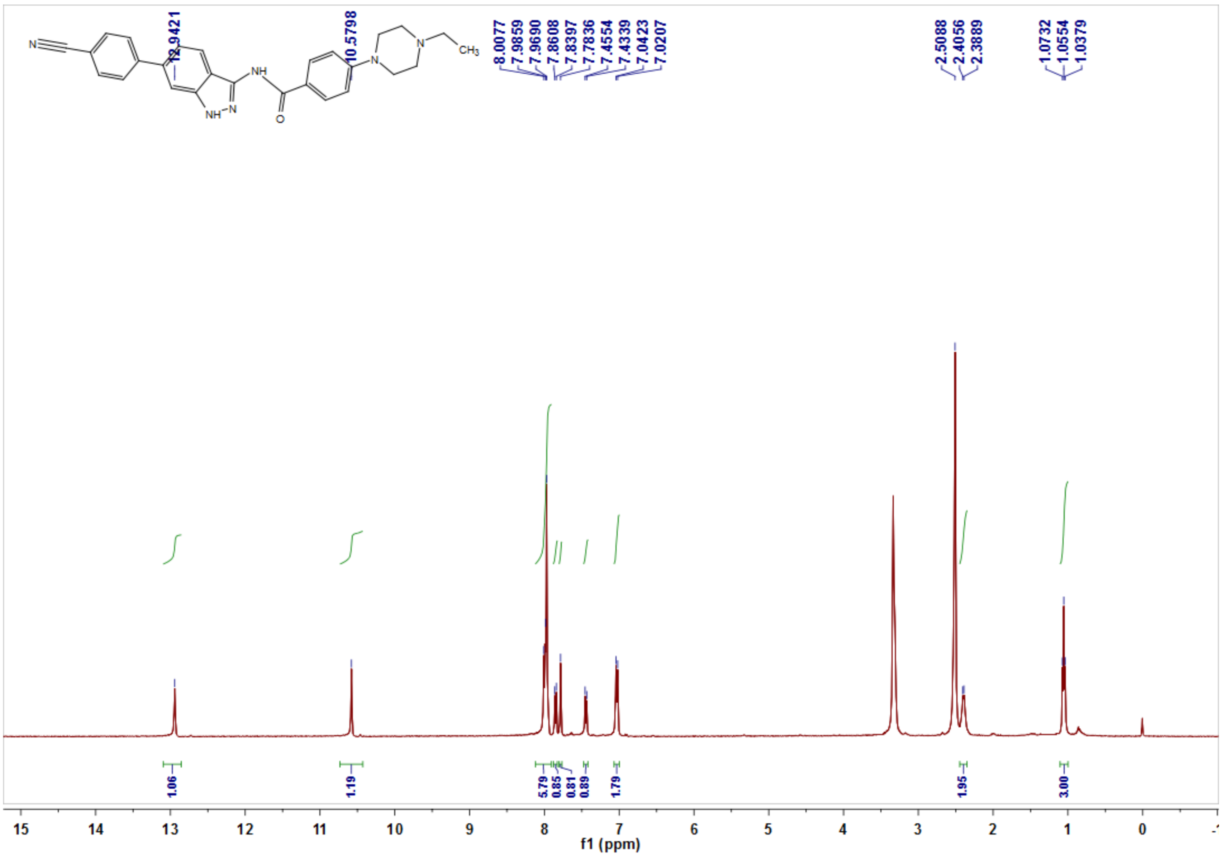

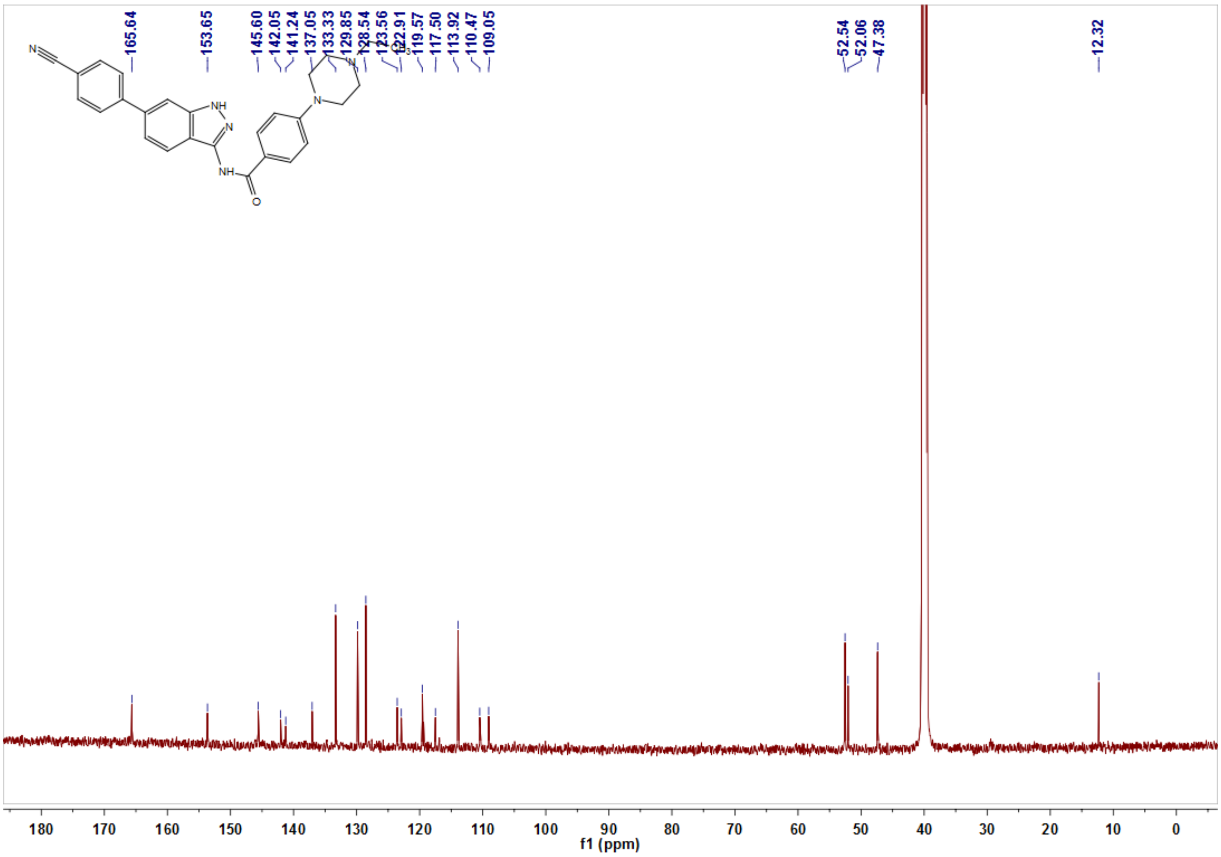

^{1}H NMR and ^{13}C NMR of compound **9t**

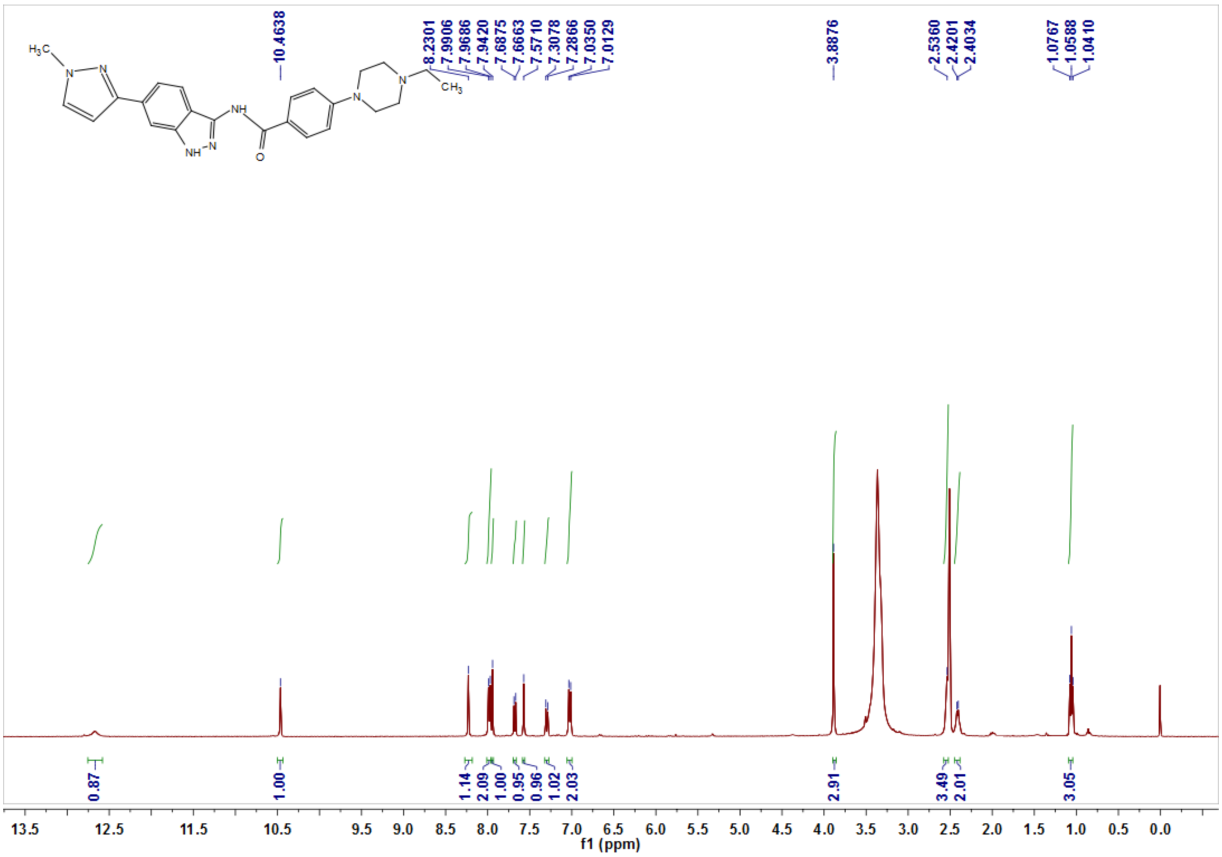

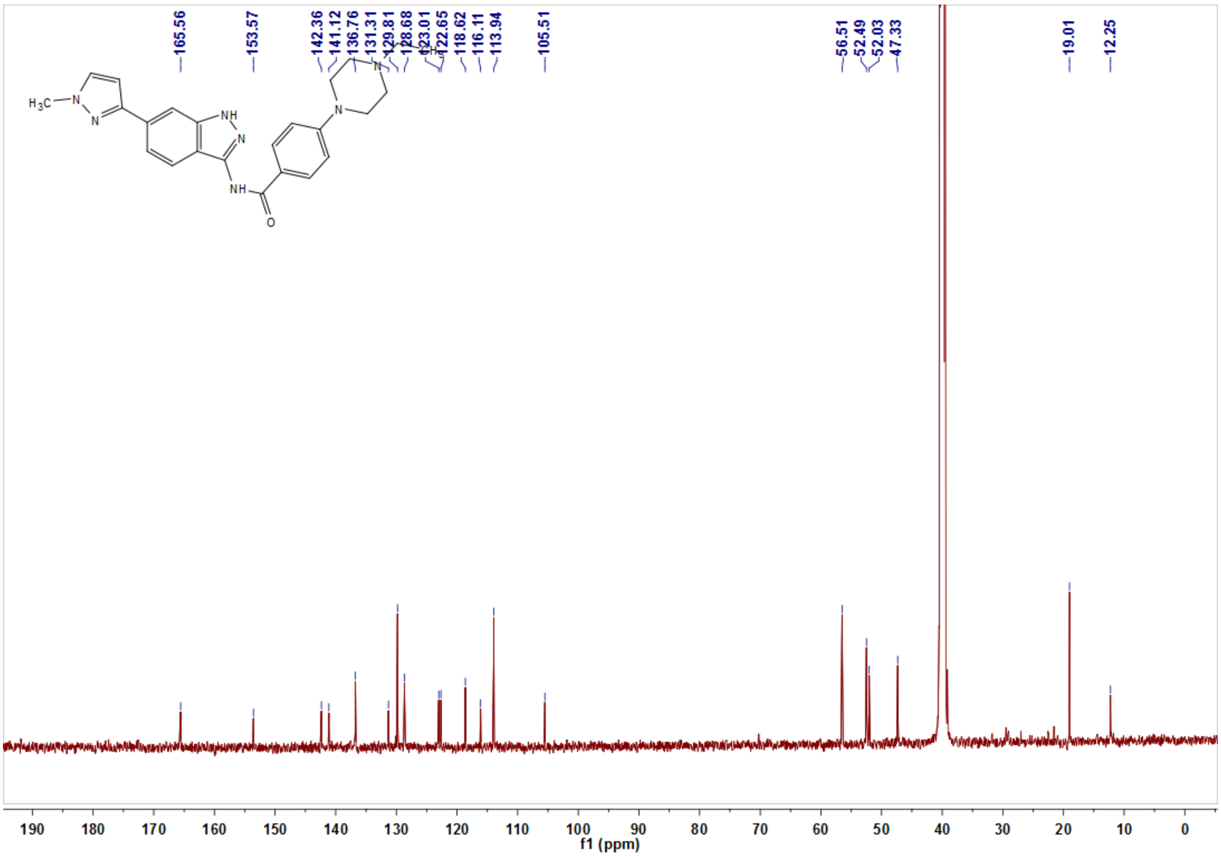

^{1}H NMR and ^{13}C NMR of compound **9u**

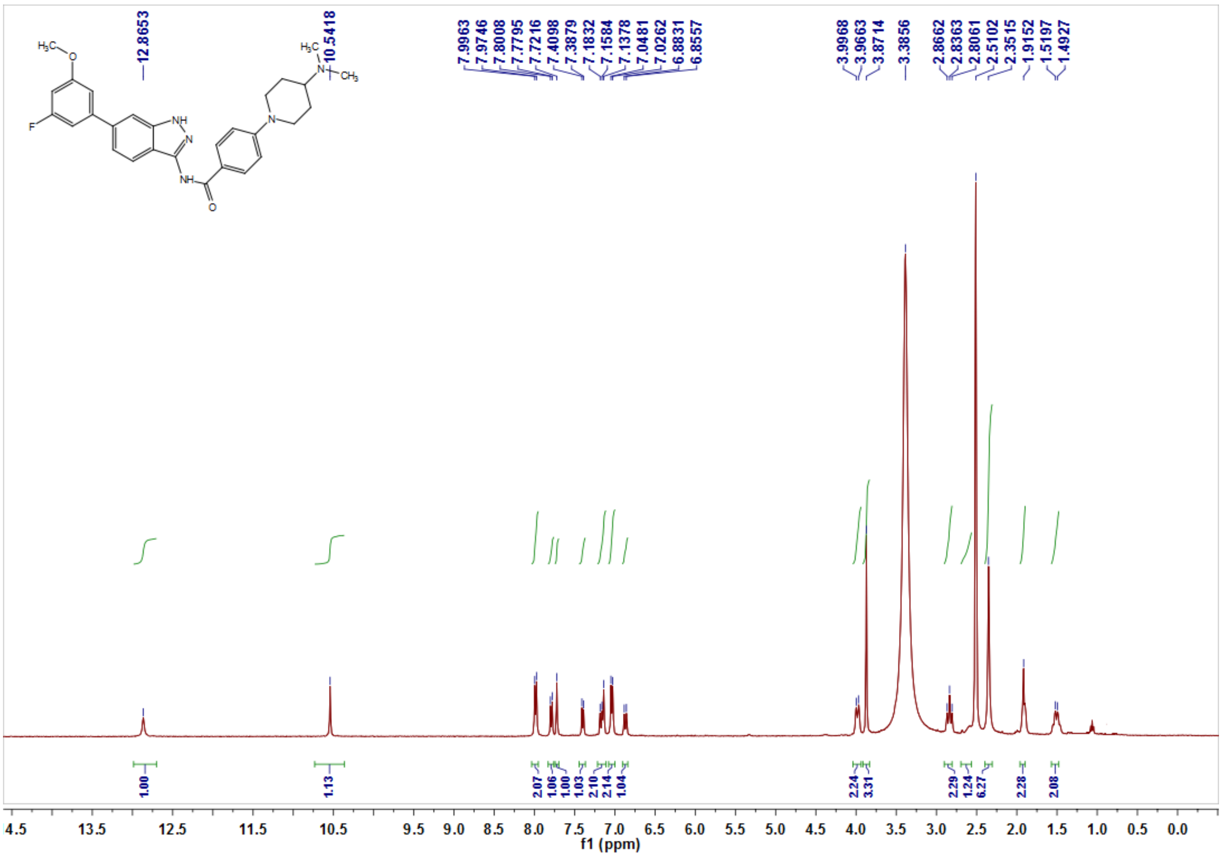

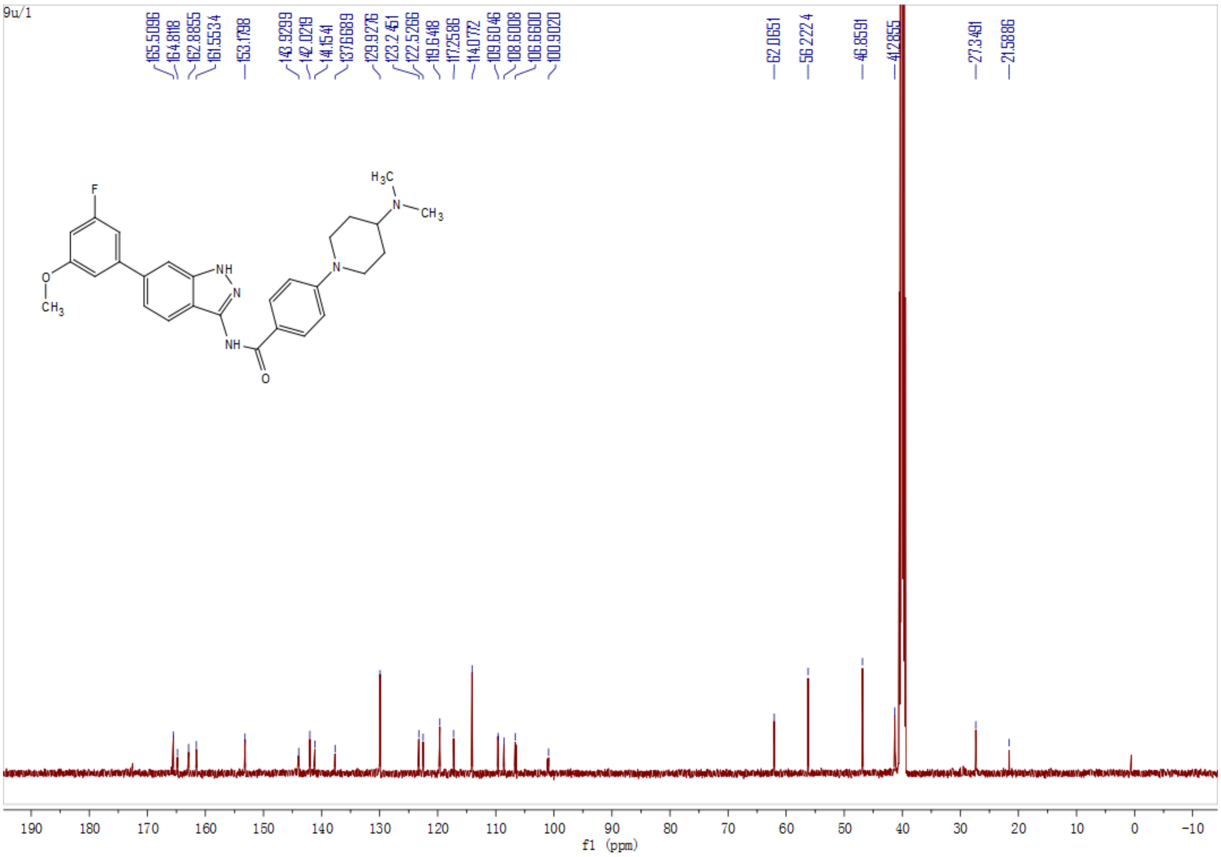

^{1}H NMR and ^{13}C NMR of compound **9v**

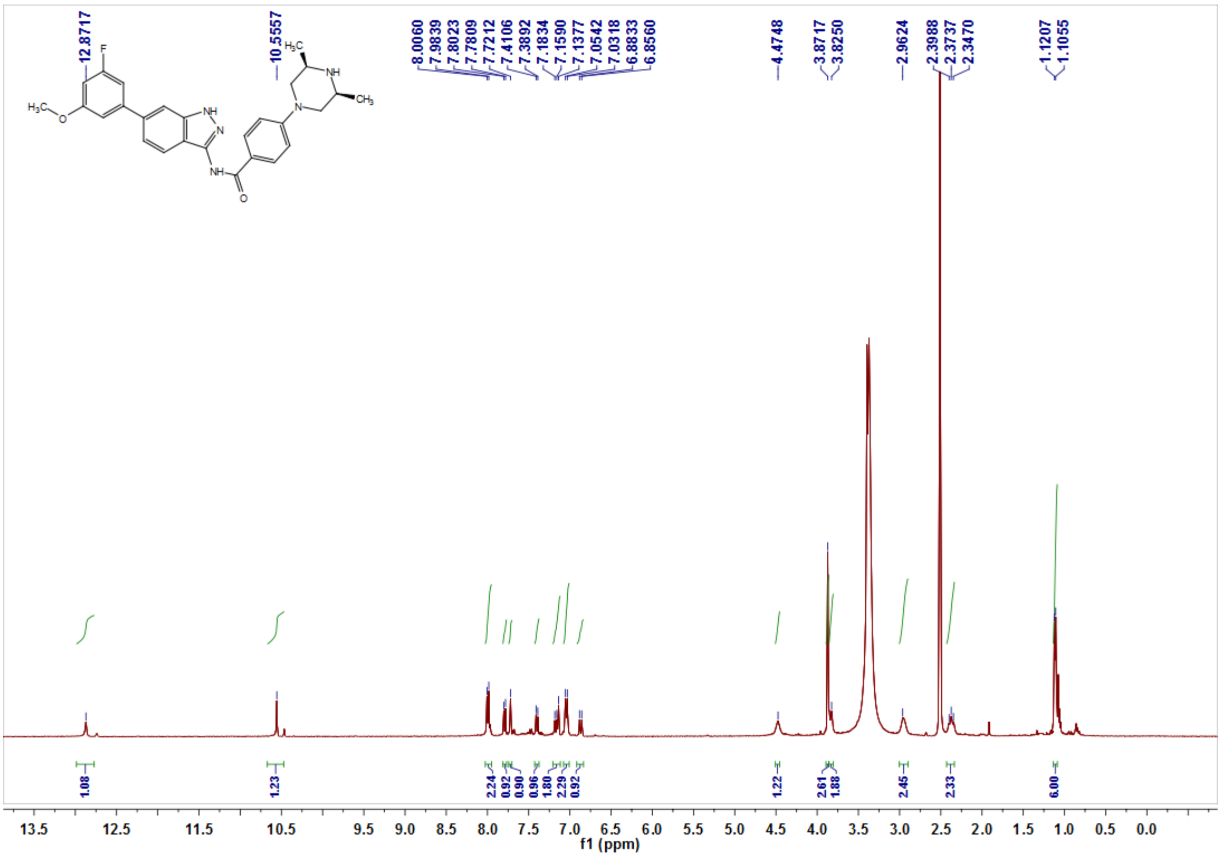

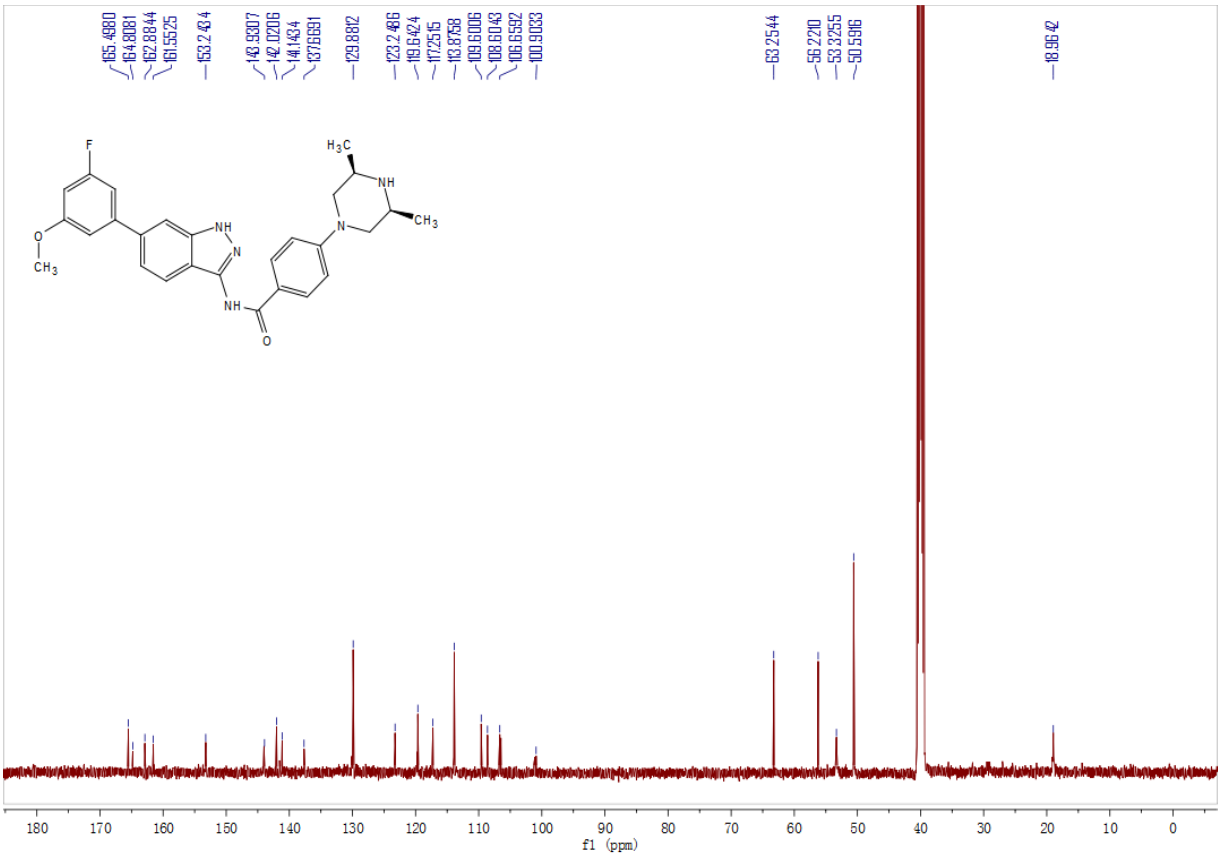

^{1}H NMR and ^{13}C NMR of compound **9w**

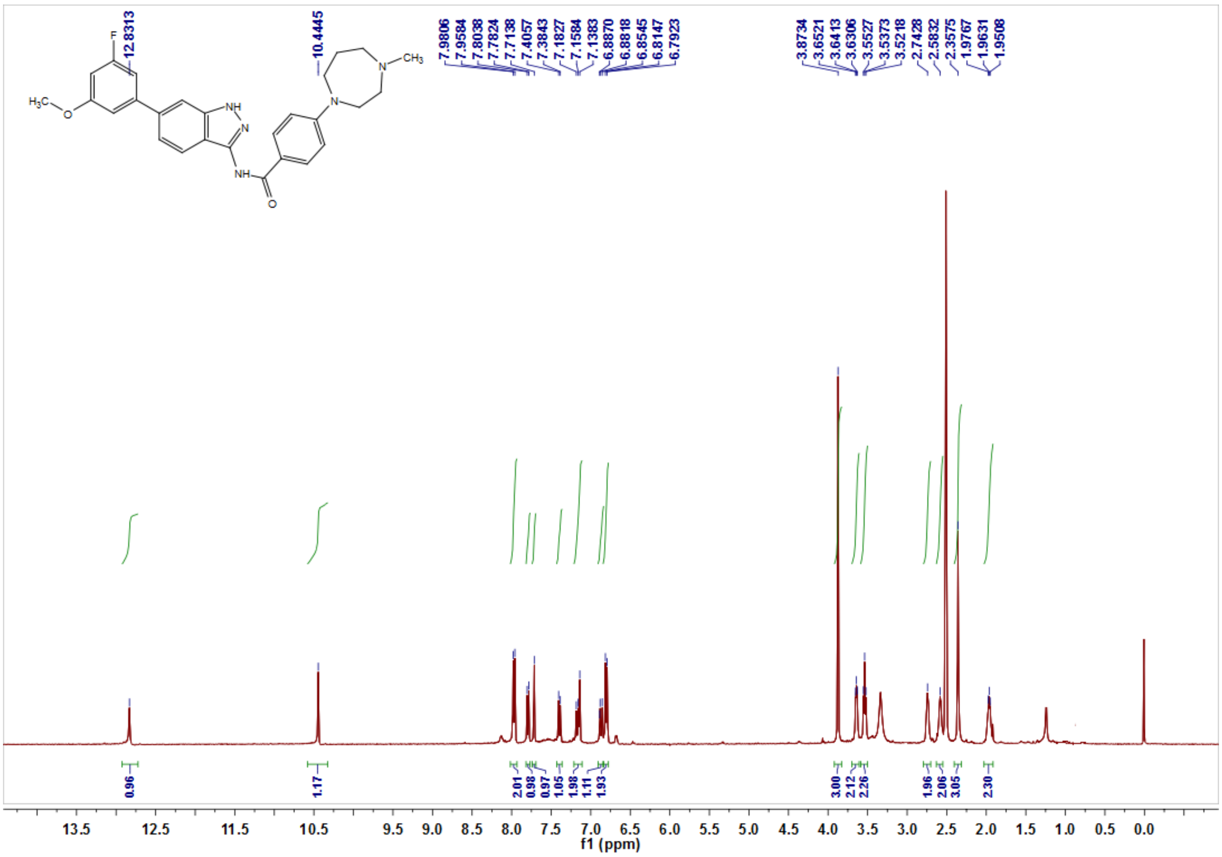

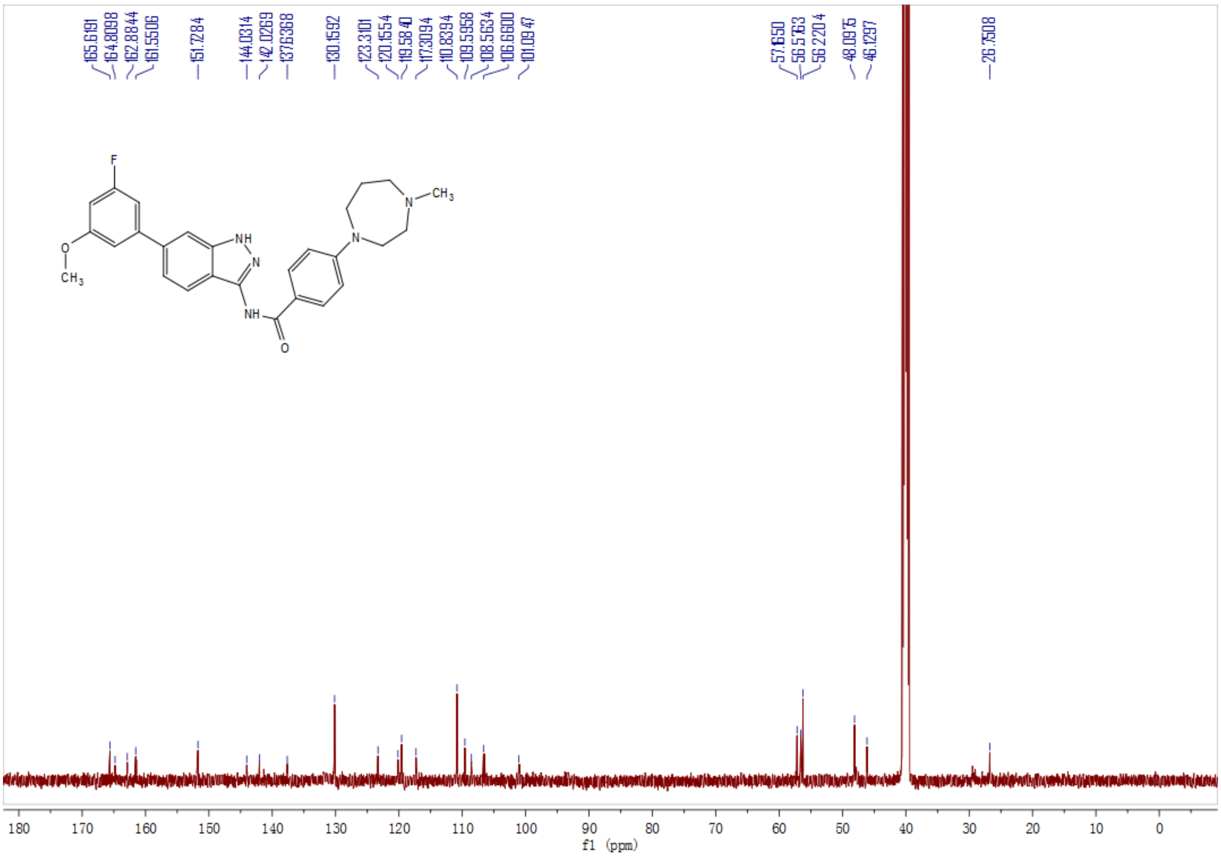

^{1}H NMR and ^{13}C NMR of compound **9x**

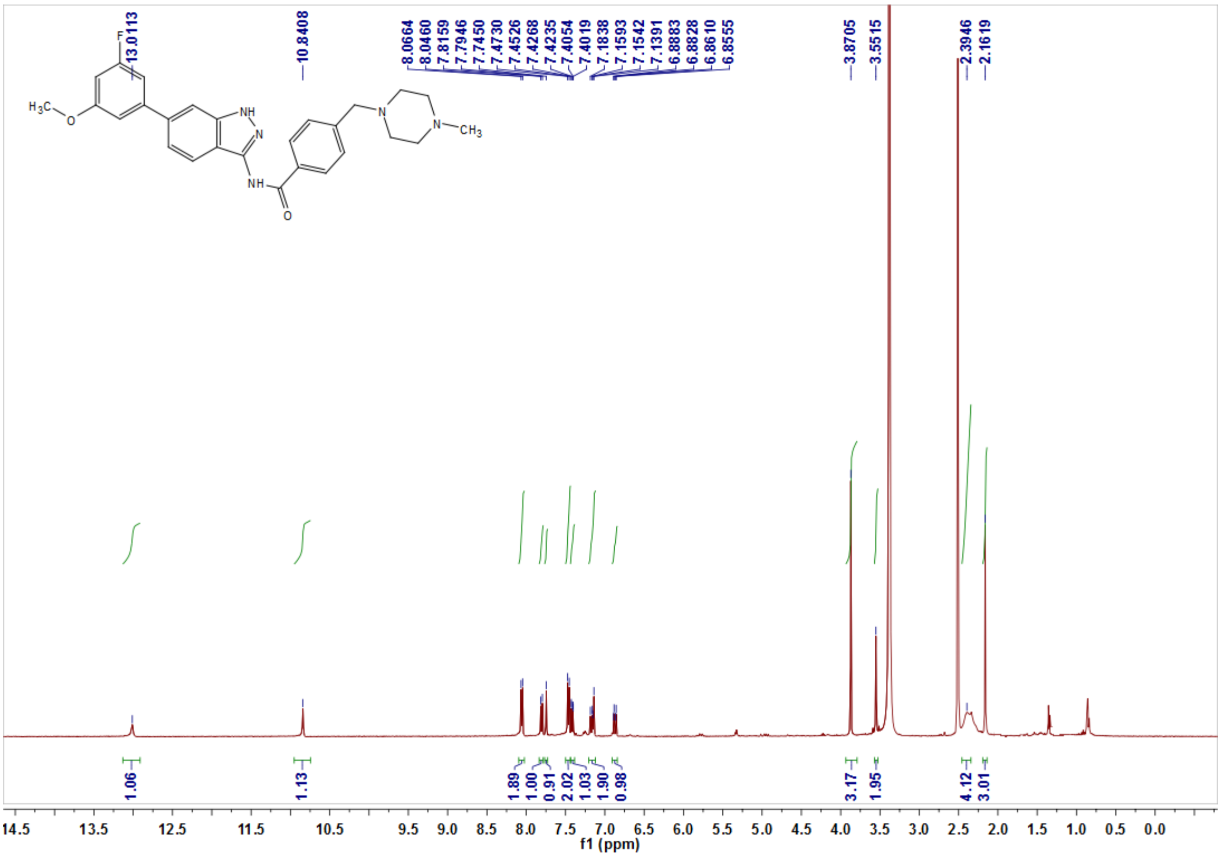

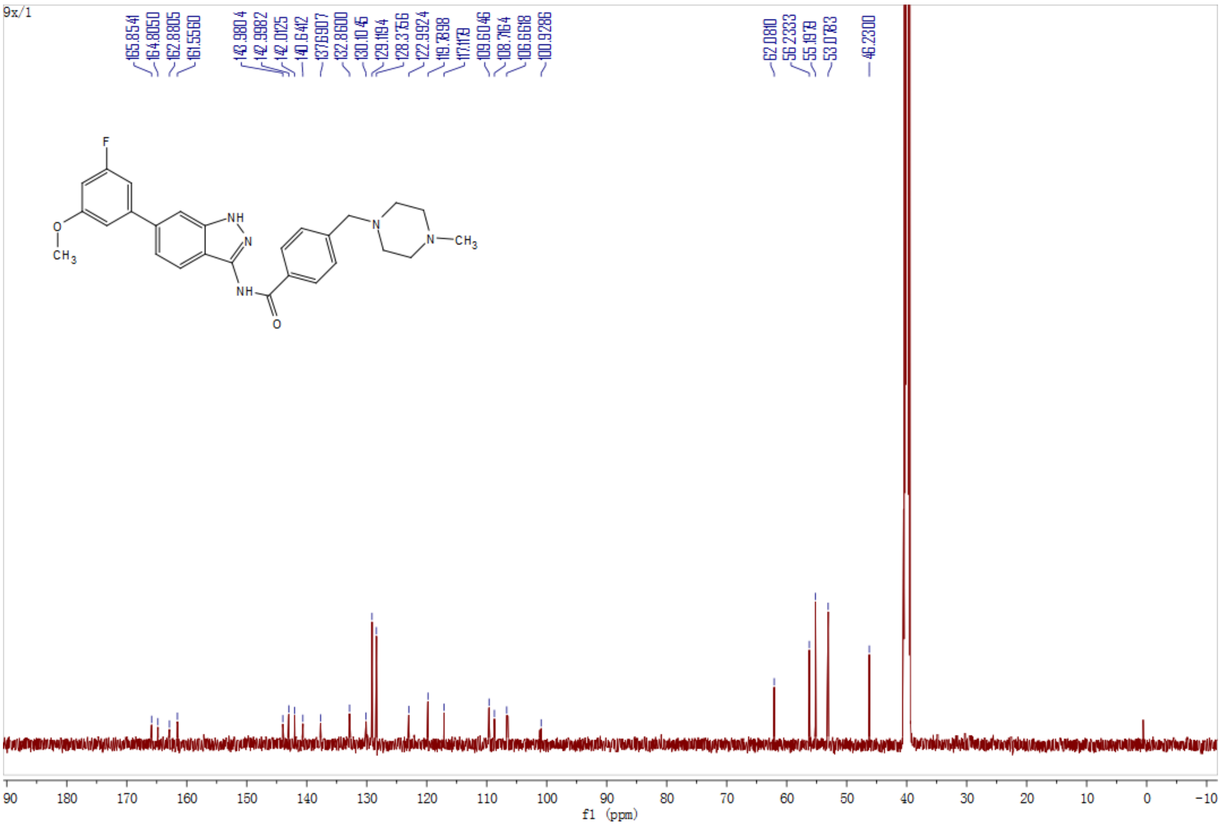

^{1}H NMR and ^{13}C NMR of compound **9y**

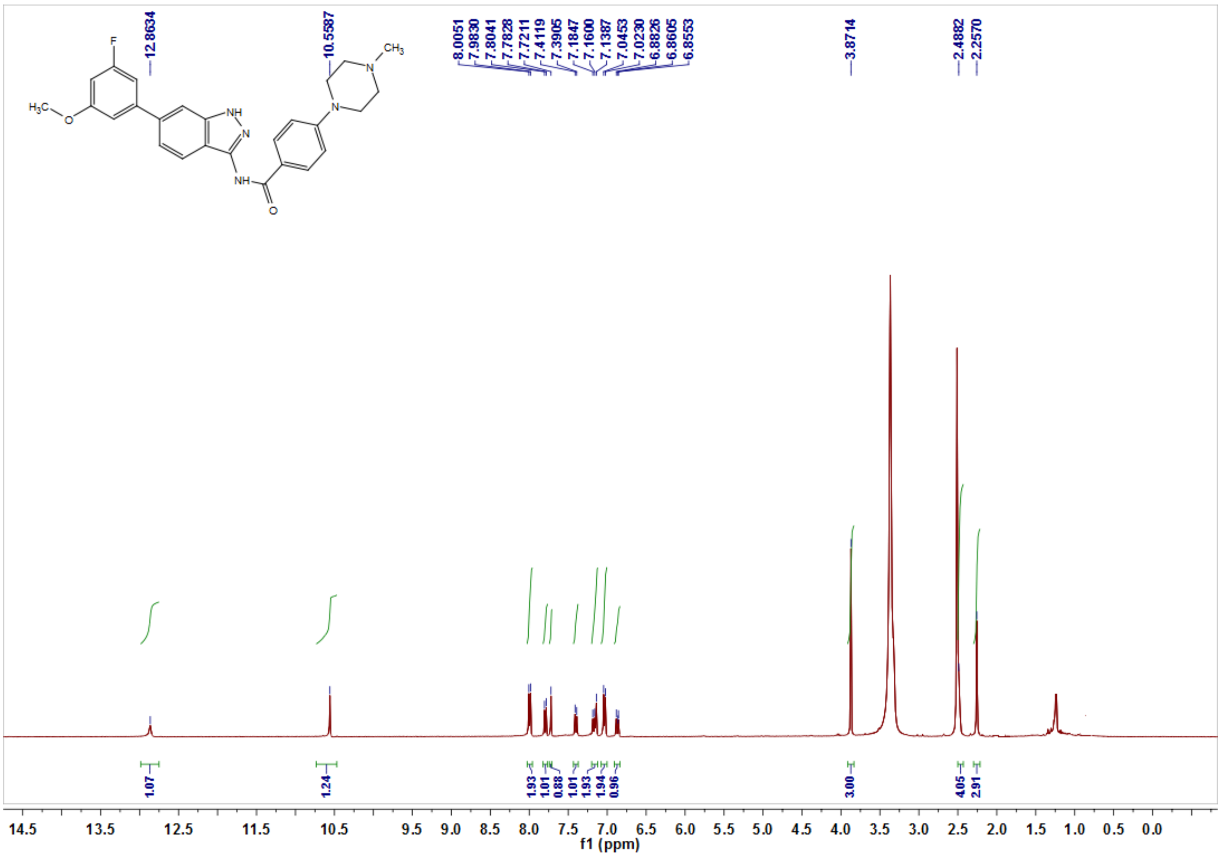

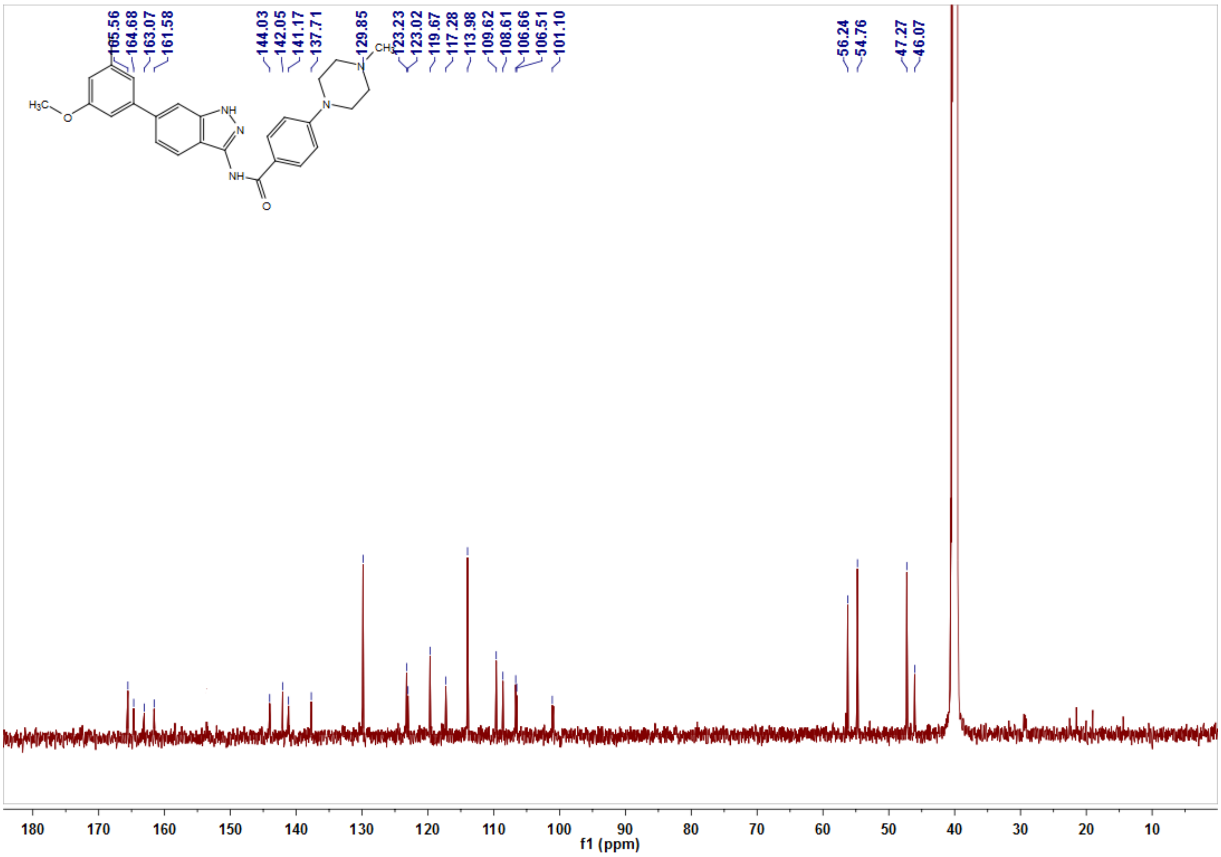

^{1}H NMR and ^{13}C NMR of compound **9z**

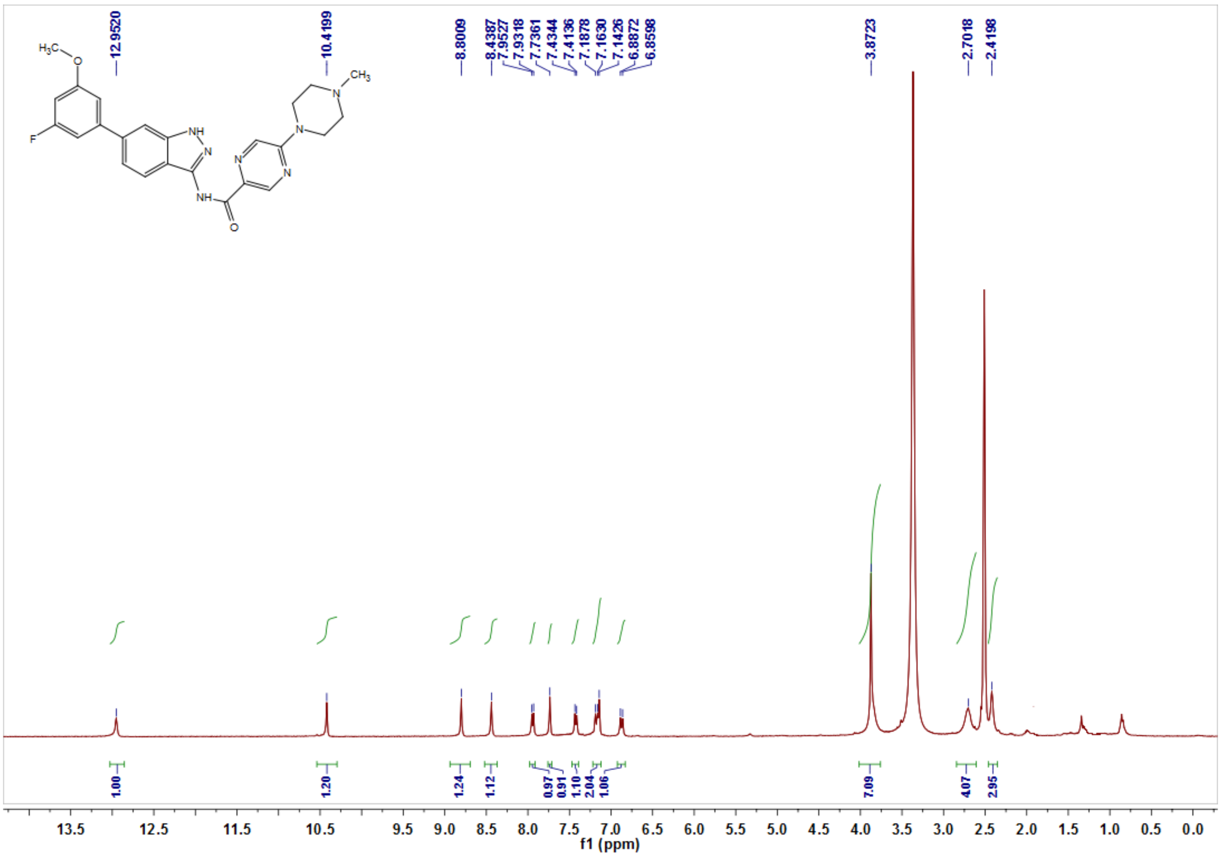

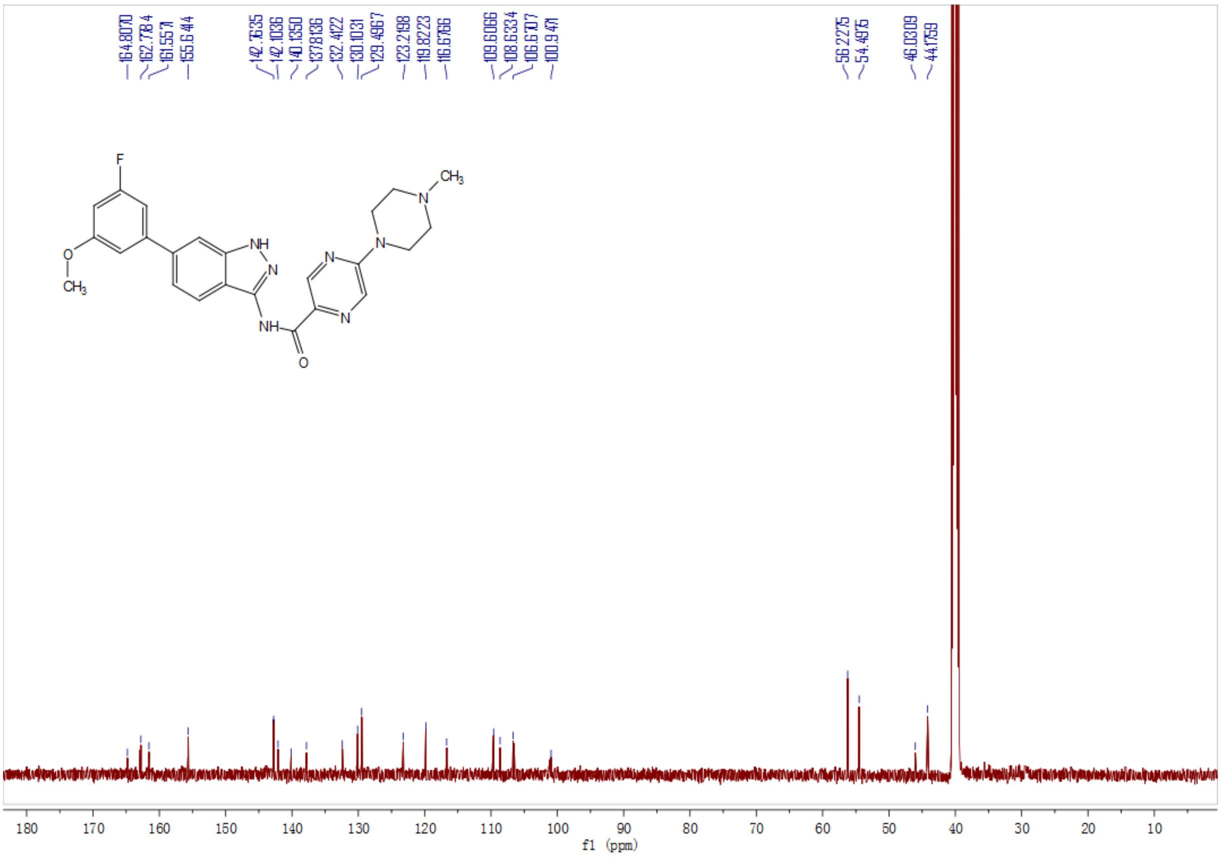

^{1}H NMR of compound **12b**

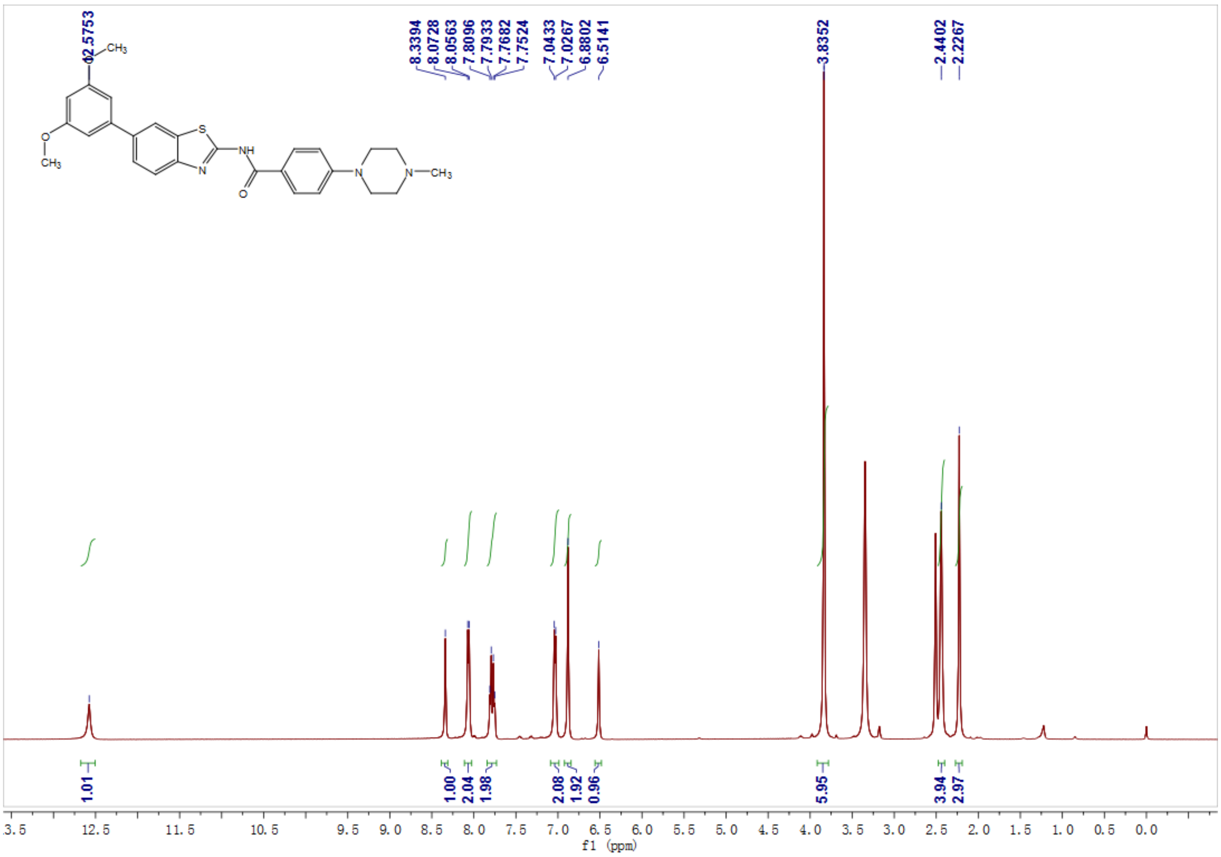

^{13}C NMR of compound **12b**

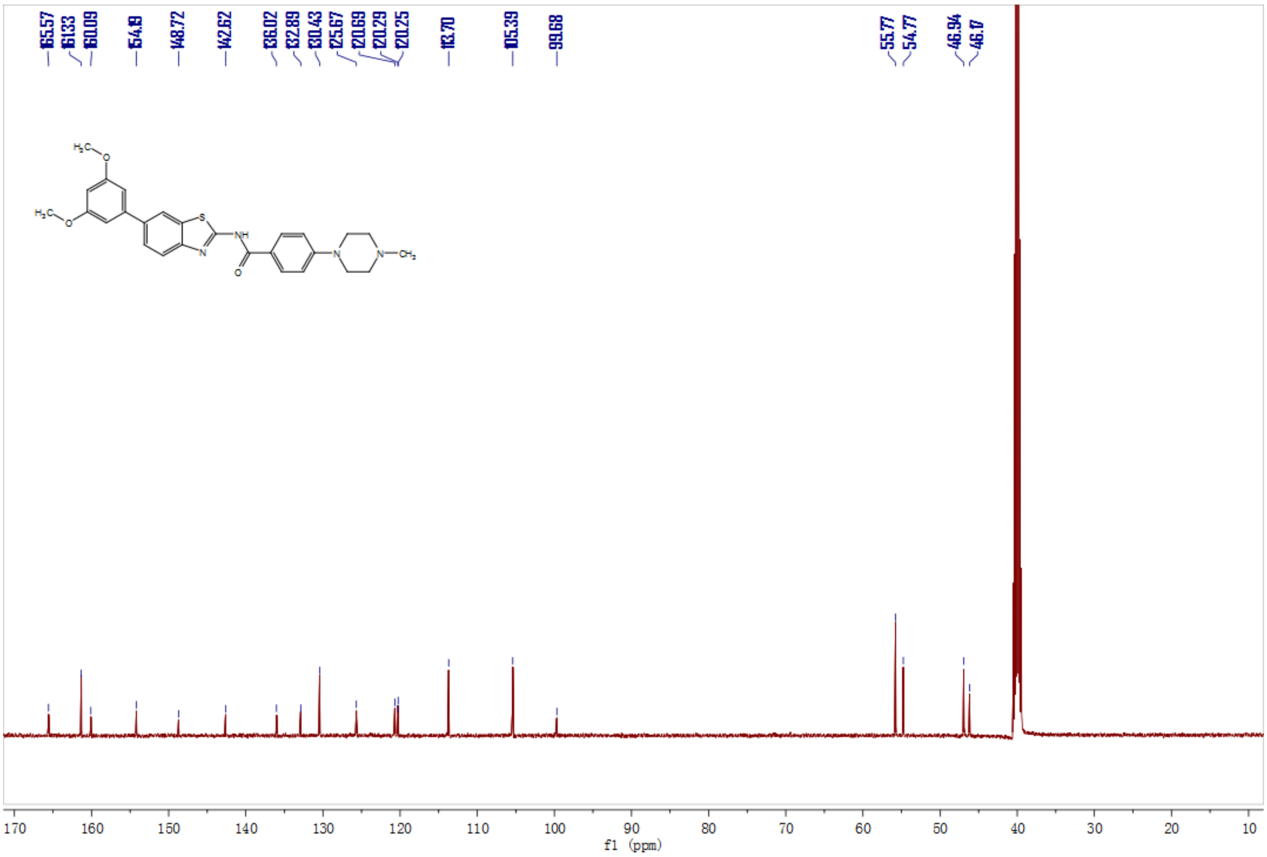

^{1}H NMR of compound **12c**

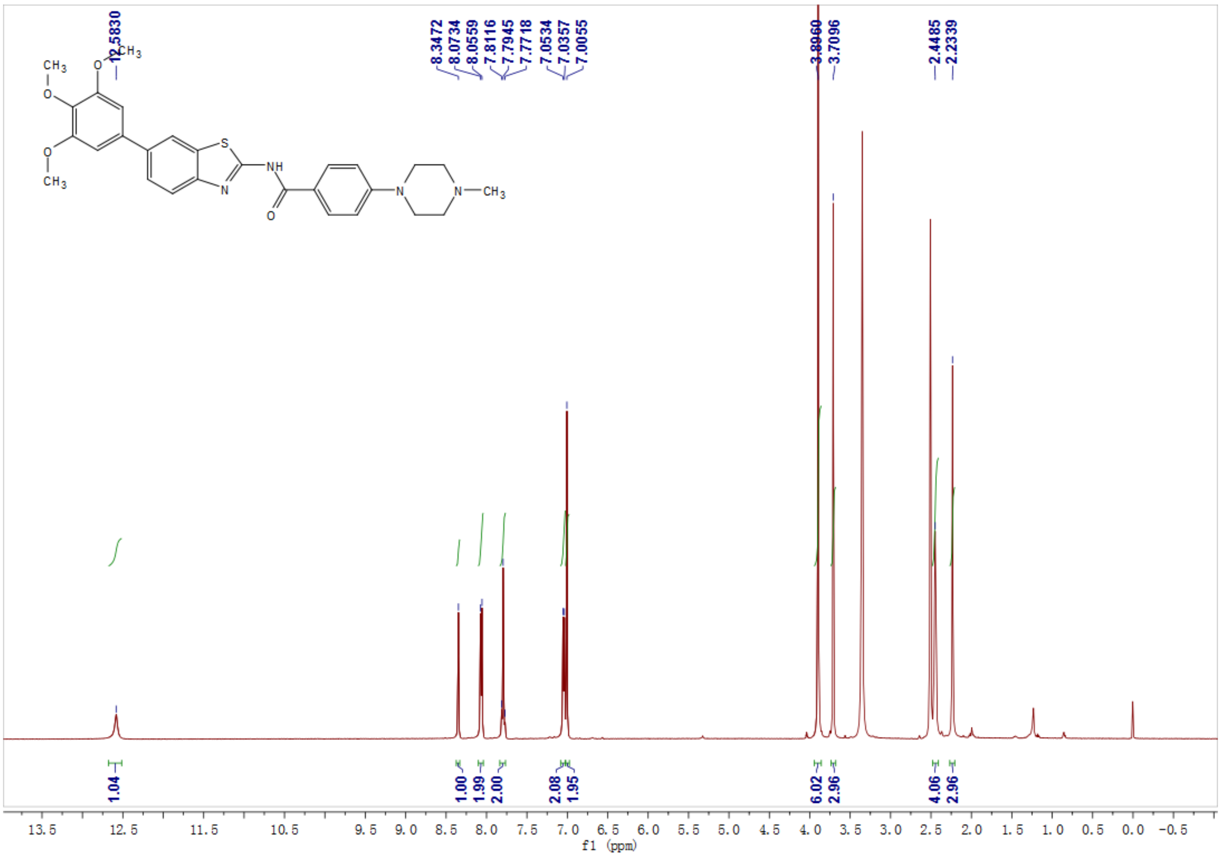

^{13}C NMR of compound **12c**

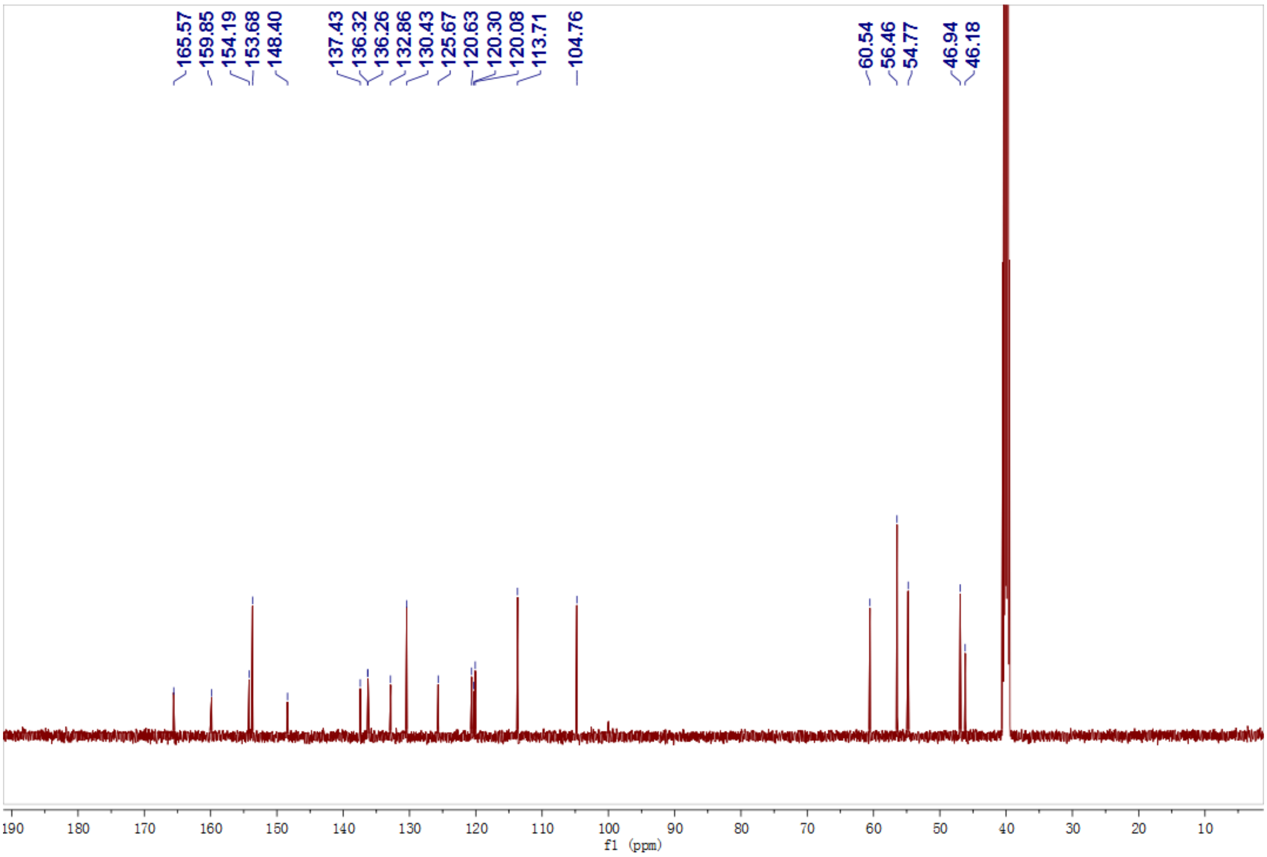

^{1}H NMR of compound **18a**

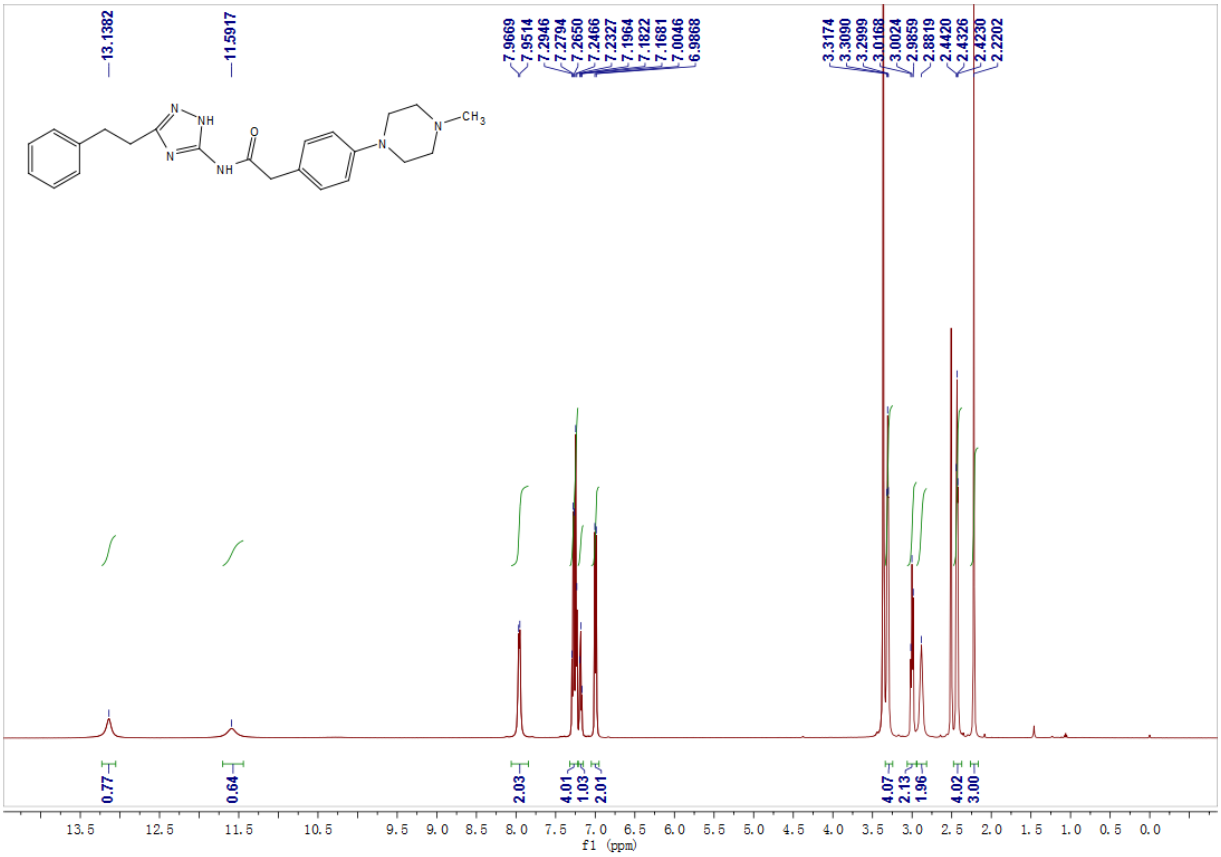

^{13}C NMR of compound **18a**

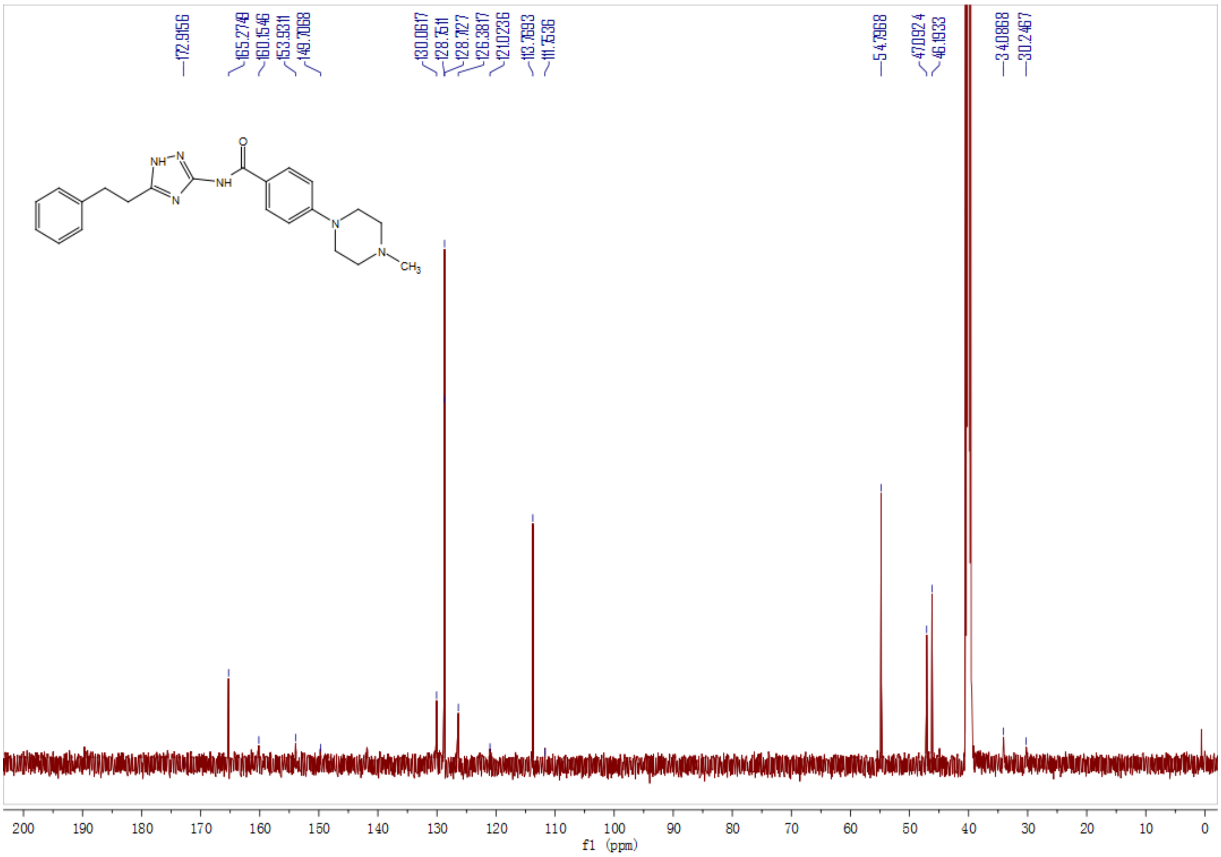

^{1}H NMR of compound **18b**

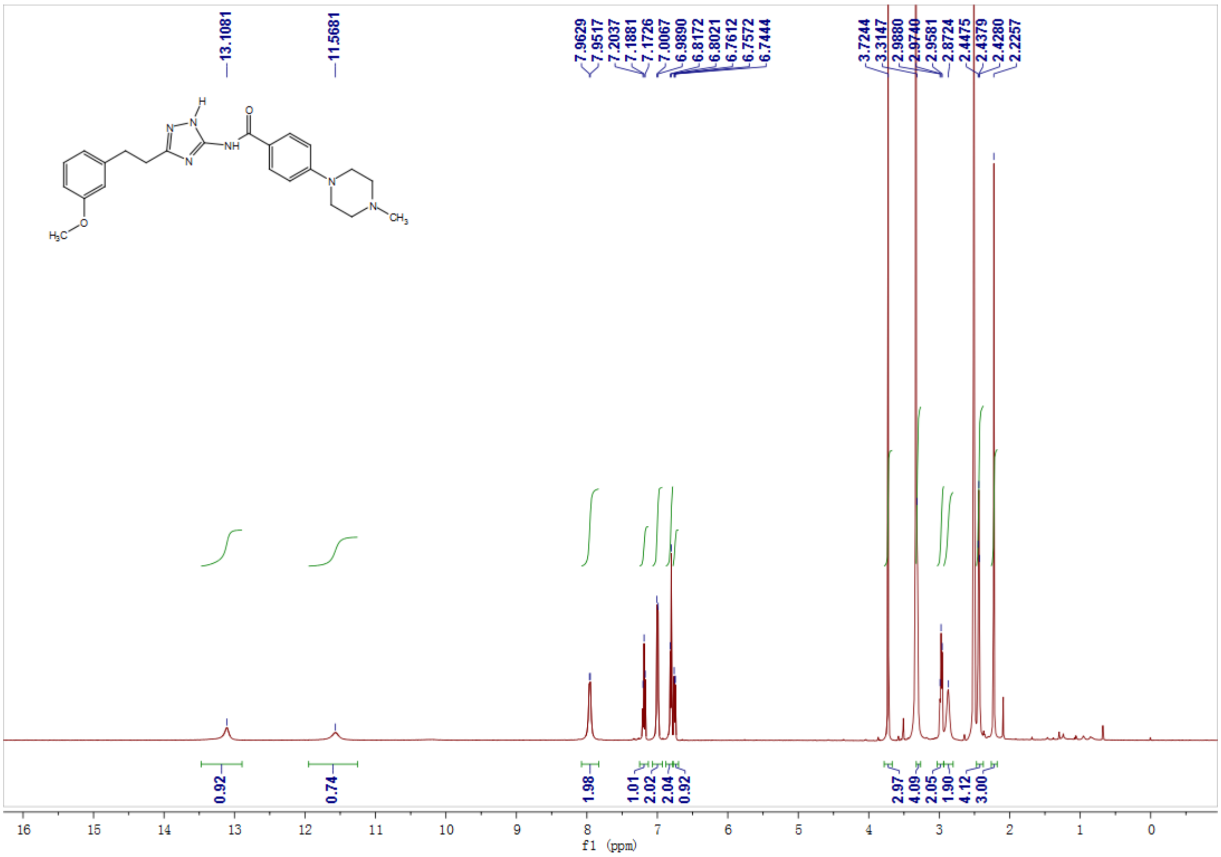

^{13}C NMR of compound **18b**

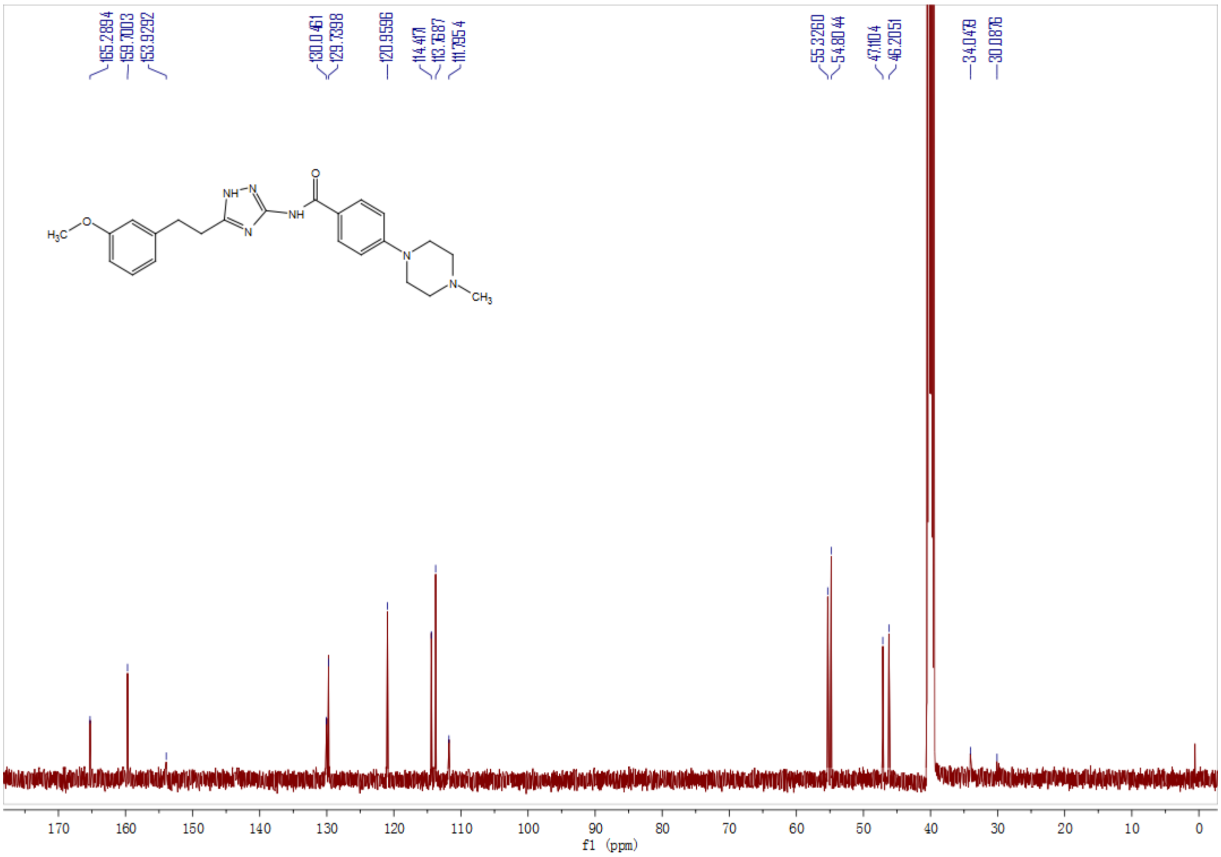

Supplement: Supplemental Material [file IENZ_A_1673745_SM3471.pdf]
